# Human GBP1 binds LPS to initiate assembly of a caspase-4 activating platform on cytosolic bacteria

**DOI:** 10.1038/s41467-020-16889-z

**Published:** 2020-06-24

**Authors:** José Carlos Santos, Dave Boucher, Larisa Kapinos Schneider, Benjamin Demarco, Marisa Dilucca, Kateryna Shkarina, Rosalie Heilig, Kaiwen W. Chen, Roderick Y. H. Lim, Petr Broz

**Affiliations:** 1 Department of Biochemistry, University of Lausanne, Chemin des Boveresses 155, 1066 Epalinges, Switzerland; 2 Biozentrum, University of Basel, Klingelbergstrasse 50/70, 4056 Basel, Switzerland

**Keywords:** Infection, Inflammasome, Innate immunity, Pattern recognition receptors

## Abstract

The human non-canonical inflammasome controls caspase-4 activation and gasdermin-D-dependent pyroptosis in response to cytosolic bacterial lipopolysaccharide (LPS). Since LPS binds and oligomerizes caspase-4, the pathway is thought to proceed without dedicated LPS sensors or an activation platform. Here we report that interferon-induced guanylate-binding proteins (GBPs) are required for non-canonical inflammasome activation by cytosolic *Salmonella* or upon cytosolic delivery of LPS. GBP1 associates with the surface of cytosolic *Salmonella* seconds after bacterial escape from their vacuole, initiating the recruitment of GBP2-4 to assemble a GBP coat. The GBP coat then promotes the recruitment of caspase-4 to the bacterial surface and caspase activation, in absence of bacteriolysis. Mechanistically, GBP1 binds LPS with high affinity through electrostatic interactions. Our findings indicate that in human epithelial cells GBP1 acts as a cytosolic LPS sensor and assembles a platform for caspase-4 recruitment and activation at LPS-containing membranes as the first step of non-canonical inflammasome signaling.

## Abstract (web summary)

Detection of LPS derived from Gram-negative bacteria by innate immune receptors is a critical step in the host response. Here Santos and colleagues show human GBP1 binds to LPS resulting in non-canonical inflammasome activation.

## Introduction

Detection of lipopolysaccharide (LPS) is central to host defense against Gram-negative bacterial infections and to the pathogenesis of sepsis. Extracellular LPS is sensed by Toll-like receptor 4 (TLR4), which induces the production of cytokines via the MyD88 and TRIF signaling pathways^1^. Cytosolic LPS, on the other hand, is detected by the so-called non-canonical inflammasome, which controls the activation of caspase-4/−5 in humans and caspase-11 in mice^2–6^. These caspases cleave the pore-forming cell death effector gasdermin D (GSDMD) to induce pyroptosis and cytokine release. While the activation of other caspases requires their recruitment to multiprotein platforms formed by dedicated sensor and adaptor proteins (e.g., DISC, apoptosome and canonical inflammasome), no comparable platform has yet been reported for caspase-4/−5 or −11. Instead, their activation appears to involve a new mode of pattern recognition in which caspase-4/−11 act both as sensor and executor without the need for additional adaptor proteins or co-factors^5^. This model was proposed based on the observation that caspases-4/−11 binds the highly hydrophobic lipid A moiety of LPS through their CARD (caspase recruitment domain), resulting in their oligomerization and activation^5^. However, since LPS is hydrophobic and normally present within bacterial membranes, it is conceivable that cytosolic LPS sensing could require accessory factors in analogy to LPS-binding protein (LBP) or cluster of differentiation 14 (CD14) that are required for TLR4 signaling. LBP binds to LPS-containing outer membrane of bacteria and promotes the transfer of LPS onto CD14, which then delivers LPS to the MD-2/TLR4 complex^7,8^.

Caspase-11 activation in mouse macrophages transfected with LPS or infected with Gram-negative bacteria requires the expression of interferon (IFN)-inducible GTPases, which include the GBPs (guanylate-binding proteins) or IRGs (immunity-related GTPases)^9–12^. These GTPases are highly upregulated after type-I or type-II IFN priming, and essential for cell-autonomous immunity against a variety of viruses, bacteria and parasites^13^. In macrophages, several GBPs as well as Irgb10 were found to target intracellular Gram-negative bacteria, such as *Salmonella enterica* serovar Typhimurium (referred to as *Salmonella*), *Francisella novicida* and *Escherichia coli*. Since this recruitment correlated with bacterial lysis and the activation of caspase-11, it gave rise to a model in which GBPs recruit Irgb10 towards bacterial membranes, thereby unleashing an Irgb10-dependent membranolytic activity that kills the pathogen and concomitantly liberates LPS for the activation of non-canonical inflammasome^11^. However, since humans lack the IRG family (except for a truncated IRGM copy and IRGC), and GBPs are nevertheless required for LPS-induced caspase-4 activation, the current model needs to be confirmed in human cells^14,15^.

Here we report that IFNγ priming and the induction of GBPs are necessary for caspase-4 activation in human epithelial cells and monocytes/macrophages during infection with the Gram-negative bacterium *Salmonella* or after cytosolic LPS delivery by transfection or electroporation. We show that human GBP1 targets cytosolic *Salmonella* seconds after the bacteria escape from the vacuole and enter into the cytosol, and that GBP1 initiates the hierarchical recruitment of GBP2-4 and the assembly of a GBP coat on cytosolic bacteria. This GBP coat does not induce bacteriolysis, but instead initiates the recruitment and activation of caspase-4 to the surface of cytosolic bacteria. Human GBPs play distinct functional roles in this process: GBP1 together with GBP4 recruit caspase-4, whereas GBP3 is mainly required for caspase-4 activation. Investigating the mechanism by which GBP1 recognizes cytosol-exposed bacteria, we demonstrate that LPS associates with GBP1 in pyroptotic cells and that recombinant GBP1 binds LPS with high affinity. Monomeric GBP1 associates with LPS micelles to form a high-molecular weight complex upon incubation with LPS, and this association occurs via electrostatic interactions involving negative charges on LPS. Consistently, mutagenesis of GBP1 shows that positively charged residues are necessary for LPS binding and recruitment to bacteria. In conclusion, we show that GBP1 acts as a bona-fide cytosolic LPS sensor that detects and targets the LPS-containing membranes of Gram-negative bacteria, where it assembles a platform that promotes caspase-4 recruitment and activation.

## Results

### *Salmonella*-induced caspase-4 activation requires IFNγ priming

**Fig. 1:**
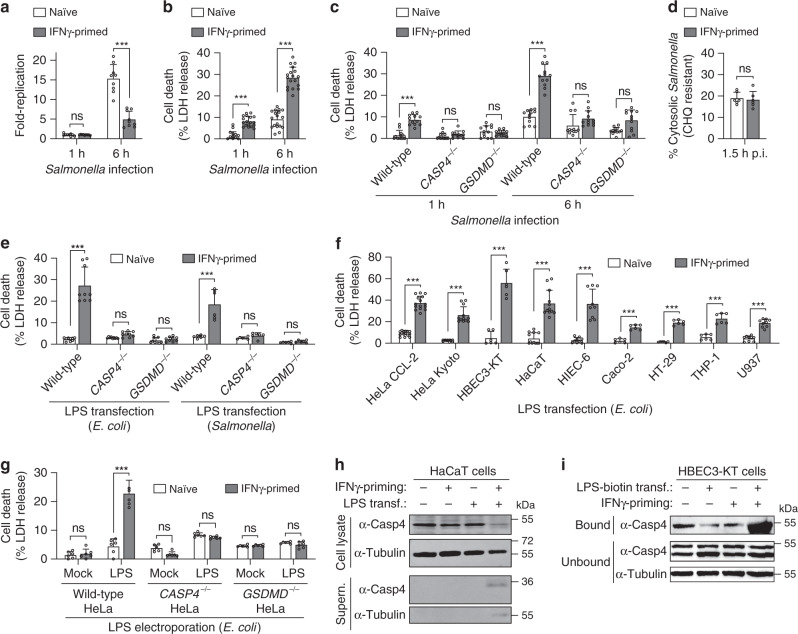
IFNγ priming is required for LPS-induced caspase-4 activation in human epithelial cells. **a**–**c** Intracellular bacterial fold-replication (**a**) and release of LDH (**b**, **c**) in naive or IFNγ-primed wild-type, *CASP4*^–/–^ or *GSDMD*^–/–^ HeLa cells, at 1 or 6-h post-infection (p.i.) with *Salmonella*. Cells in 96-well plates were infected for 30 min, washed and gentamicin was added to kill extracellular bacteria. At the indicated time points supernatant was collected to determine the release of LDH, and then cells were lysed and the number of viable intracellular bacteria was determined by counting colony forming units (CFUs). The bacterial fold-replication was calculated relative to 1 h p.i. **d** Percentage of CHQ-resistant cytosolic *Salmonella* in naive or IFNγ-primed HeLa at 1.5 h p.i. Cells were infected for 30 min as in (**a**) and then treated with gentamicin ± CHQ for an additional 1 h before cells were lysed and bacteria counted by CFUs. The percentage of cytosolic bacteria was calculated as the ratio of (CHQ + gentamicin^resistant^ / gentamicin^resistant^). **e**–**g** Release of LDH from naive or IFNγ-primed cells, 5 h after transfection with LPS (2.5 µg/50,000 cells) or 3–4 h after electroporation with LPS (300 ng/50,0000 cells). **h** Western blot analysis of full length (p43) and cleaved (p32) caspase-4 in the supernatants and cell lysates from naive or IFNγ-primed HaCaT cells, upon transfection with *E. coli* LPS LPS (2.5 µg/50,000 cells). **i** Streptavidin pull-down assay of the binding of biotin-conjugated LPS to endogenous caspase-4 from the lysates of naive or IFNγ-primed HBEC3-KT. Cells in 6-well plates were transfected with LPS-biotin (10 µg) or left untransfected, and biotinylated substrate was pulled down using equal amounts of streptavidin magnetic beads, which were then eluted in equal volumes of SDS-PAGE reducing sample buffer. Streptavidin-bound and -unbound fractions were analyzed by immunoblotting for caspase-4. Graphs show the mean ± SD, and data are pooled from two to six independent experiments performed in triplicate (**a**–**g**) or representative of two (**h**, **i**) independent experiments. *** *P* < 0.001; ns, not significant; two-tailed *t*-test.

To study the human non-canonical inflammasome and its modulation by priming, we infected naive or IFNγ-primed HeLa cells, which lack canonical inflammasome pathways, with the facultative intracellular bacterium *Salmonella*. Since HeLa cells express TLR4 but not MD-2 and are thus not responsive to extracellular bacterial LPS^16^, we primed the cells with IFNγ, a cytokine that also plays a critical role in intestinal immunity against *Salmonella*^17^. *Salmonella* replicated rapidly in naive HeLa but was strongly restricted in IFNγ-primed cells (Fig. 1a), despite similar levels of bacterial invasion (Supplementary Fig. 1a). Strikingly, IFNγ-primed HeLa cells underwent lytic cell death with typical features of pyroptosis, such as plasma membrane swelling and ballooning, and nuclear condensation (Fig. 1b, Supplementary Fig. 1b–e and Supplementary Movies 1, 2), and released mature IL-18 (Supplementary Fig. 1f). Since in epithelial cells a subset of *Salmonella* escape from the *Salmonella*-containing vacuole (SCV) into the cytosol within the first hour after entry^18,19^, we hypothesized that *Salmonella* could activate the non-canonical inflammasome as previously observed in mouse macrophages^20^. To test this, we infected naive or IFNγ-primed wild-type, *CASP4*^–/–^ and *GSDMD*^–/–^ HeLa (Supplementary Fig. 1g). Deletion of *CASP4* or *GSDMD* did not alter bacterial invasion, but abrogated *Salmonella*-induced IFNγ-dependent cell death (Fig. 1c and Supplementary Fig. 1h–k), confirming that *Salmonella* infection of HeLa cells activates the non-canonical inflammasome in an IFNγ-dependent manner. While bacterial replication was increased, IFNγ priming still partially reduced intracellular bacterial replication in *CASP4*^–/–^ and *GSDMD*^–/–^ HeLa (Supplementary Fig. 1l), suggesting that cell death was not the only mechanism by which IFNγ restricts bacterial growth. This finding was confirmed using *Salmonella* expressing P*uhpT*-GFP, a reporter for cytosolic replication (e.g., GFP under the control of the hexose phosphate transporter promoter, which responds to exogenous glucose-6-phosphate^21^ found exclusively in the host cytosol) (Supplementary Fig. 1m–o). Furthermore, using a chloroquine (CHQ)-resistance assay, an antimicrobial agent that only reaches bactericidal levels when concentrated within endocytic compartments^22,23^, we found that IFNγ priming mainly restricted cytosolic *Salmonella* (Supplementary Fig. 1p, q) thus reducing hyper-replication of the cytosolic population of *Salmonella* (Supplementary Fig. 1r, s)^24,25^. Thus, IFNγ controls a major caspase-4- and GSDMD-dependent mechanism that restricts cytosolic *Salmonella* replication by inducing host cell pyroptosis, and a minor mechanism that acts independently of cell death.

We next determined whether IFNγ was necessary to promote access of *Salmonella* or their LPS to the cytosol by quantifying bacterial resistance to CHQ at 1.5 h post-infection (p.i.). When compared with naive cells, IFNγ priming did not induce *Salmonella* escape to the host cytosol (Fig. 1d), indicating that IFNγ controlled the detection of LPS after bacterial entry into the cytosol. To confirm this, we next transfected cells with ultrapure *E. coli* or *Salmonella* LPS. Similarly to *Salmonella* infection, LPS transfection only caused cell death in IFNγ-primed HeLa and was completely abrogated by deletion of *CASP4* or *GSDMD* (Fig. 1e and Supplementary Fig. 2a). IFNγ-priming was also required for pyroptosis and IL-18 release after LPS transfection (Fig. 1f and Supplementary Fig. 2b–d) and after LPS electroporation (Fig. 1g and Supplementary Fig. 2e) in a panel of human cell lines and primary cells, including human small intestinal epithelial cells (HIEC-6). To further substantiate that caspase-4 activation requires IFNγ, we transfected LPS into naive or IFNγ-primed cells and pulled down active caspase-4 using a cell-permeable pan-caspase activity probe, biotin-VAD(Ome)-fmk (bVAD-fmk)^26^. Active caspase-4 was only pulled down when cells where first primed with IFNγ and then transfected with LPS (Supplementary Fig. 2f). In accordance, LPS transfection only induced caspase-4 and GSDMD cleavage in IFNγ-primed cells (Fig. 1h and Supplementary Fig. 2g). Importantly, IFNγ-priming had no impact on the level of caspase-4 expression, since unlike murine caspase-11, caspase-4 was not induced by IFNγ (Fig. 1h, i and Supplementary Fig. 2c, g, h). To assess if IFNγ controlled caspase-4 activation upstream or downstream of LPS binding, we prepared lysates from HeLa or HBEC3-KT cells transfected with biotinylated LPS and pulled down LPS-interacting proteins with streptavidin-coupled beads. In both cell types caspase-4 could only be pulled-down with LPS in IFNγ-primed but not in naive cells (Fig. 1i and Supplementary Fig. 2h). Altogether, these findings suggest that in human epithelial cells one or several IFNγ-induced proteins are required for LPS-induced caspase-4 activation.

### GBP1 is required for non-canonical inflammasome activation

**Fig. 2:**
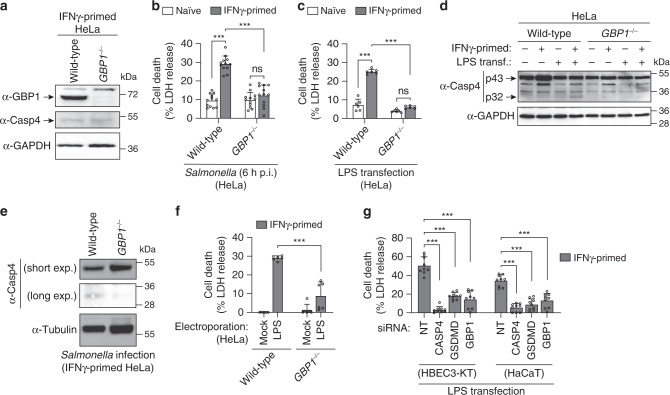
GBP1 is required for *Salmonella*- and LPS-induced caspase-4 activation to induce pyroptosis in epithelial cells. **a** Immunoblots for GBP1, caspase-4 and GAPDH (loading control) in cell lysates from IFNγ-primed wild-type or *GBP1*^–/–^ HeLa. **b**, **c** Release of LDH from naive or IFNγ-primed wild-type or *GBP1*^–/–^ HeLa after *Salmonella* infection (**b**) or after 5 h transfection with *E. coli* LPS (2.5 µg / 50,000 cells) (**c**). **d**, **e** Immunoblots for full length (p43) and cleaved (p32) caspase-4 in combined supernatants and cell lysates from naive or IFNγ-primed wild-type and *GBP1*^–/–^ HeLa, upon transfection with *E. coli* LPS for 5 h (**d**) or *Salmonella* infection (**e**). **f** Release of LDH from IFNγ-primed wild-type or *GBP1*^–/–^ HeLa, 3 h after electroporation with LPS (300 ng/50,000 cells). **g** Release of LDH in IFNγ-primed HBEC3-KT or HaCaT cells treated with non-targeting control siRNA (NT) or with siRNAs targeting *CASP4*, *GSDMD* or *GBP1*, after *E. coli* LPS transfection. Cells were treated with siRNAs for 24 h and transfected with LPS (2.5 µg / 50,000 cells) for 5 h. Graphs show the mean ± SD, and data are pooled from two (**c**, **f**), three (**g**) or four (**b**) independent experiments performed in triplicate, or representative of three independent experiments (**d**, **e**). ***P < 0.001; ns, not significant; two-tailed *t*-test.

Since caspase-11 activation in mouse macrophages requires IFN-induced GTPases, we speculated that human GBPs were necessary for LPS-induced caspase-4 activation. In agreement with previous studies^27^, we found that GBPs expression in HeLa cells was strongly upregulated by IFNγ priming (Supplementary Fig. 3a). RNA interference-mediated silencing of *GBPs* expression revealed a consistent reduction of LDH release in cells lacking GBP1 both after *Salmonella* infection as well as LPS transfection (Supplementary Fig. 3b–g). To confirm the phenotype, we generated *GBP1*^–/–^ HeLa cells by CRISPR-Cas9 genome targeting (Fig. 2a) and found that *GBP1*-deficiency completely abrogated LDH release down to the background levels that were observed in naive cells after *Salmonella* infection or LPS transfection (Fig. 2b, c and Supplementary Fig. 3h, i), without altering bacterial entry (Supplementary Fig. 3j–m). *GBP1*-deficient cells were also unable to cleave and activate caspase-4 upon LPS transfection or *Salmonella* infection (Fig. 2d, e, p32 fragment). Furthermore, GBP1 was strongly required for LDH release when LPS was delivered by electroporation (Fig. 2f). Similarly to the knockout of *CASP4* or *GSDMD*, *GBP1*-deficiency or knock-down of individual GBPs in HeLa only resulted in a partial loss of IFNγ-dependent restriction of cytosolic *Salmonella* replication (compare Supplementary Figs. 1l-s and 3n-p). Finally, GBP1 knock-down in HBEC3-KT and HaCaT cells also reduced LDH release upon LPS transfection (Fig. 2g and Supplementary Fig. 3q), demonstrating that GBP1 is important to regulate LPS-induced cell death in several human epithelial cell lines.

### GBP1 targets intracellular *Salmonella* to recruit GBP2-4

**Fig. 3:**
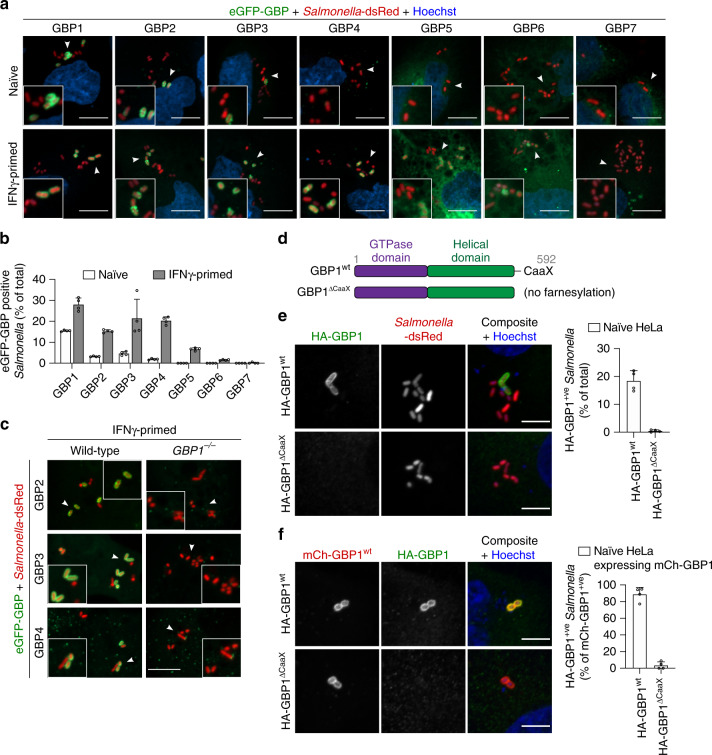
GBP1 targets *Salmonella* and controls recruitment of GBP2-4. **a** Fluorescence confocal microscopy of naive or IFNγ-primed HeLa expressing N-terminal tagged eGFP-GBP1-7 (green) and infected with *Salmonella*-dsRed (red) for 1 h. DNA was stained with Hoechst (blue). Representative confocal images are shown and scale bars correspond to 10 µm. **b** Percentage of intracellular *Salmonella* positive for eGFP-GBP1-7 in naive or IFNγ-primed HeLa, at 1 h p.i. At least 200–300 bacteria were counted per coverslip. **c** Fluorescence confocal microscopy of IFNγ-primed wild-type or *GBP1*^–/–^ HeLa expressing eGFP-GBP2-4 (green) and infected with *Salmonella*-dsRed (red) for 1 h. Representative confocal images are shown, and scale bar corresponds to 10 µm. **d** Schematic representation of wild-type GBP1 and a ΔCaaX mutant. **e**, **f** Fluorescence confocal microscopy of naive *GBP1*^–/–^ HeLa expressing HA-GBP1^wt^ or HA-GBP1^ΔCaaX^ (**e**) or co-expressing mCherry-GBP1^wt^ and HA-GBP1^wt^ or HA-GBP1^ΔCaaX^ (**f**) and infected with *Salmonella* for 1 h. HA-tagged GBP1 was visualized by immunostaining with an anti-HA antibody. Representative confocal images are shown and scale bars correspond to 5 µm. The percentage of HA-GBP1 positive bacteria was quantified by counting around 100 bacteria per coverslip. Graphs show the mean ± SD, and data are pooled from two (**b**, **e**, **f**) independent experiments performed in duplicate or representative of two (**a**, **c**, **e**, **f**) independent experiments.

Having demonstrated a role for GBPs in caspase-4 activation, we next expressed fluorescently tagged GBPs individually in naive or primed cells to determine if they target intracellular *Salmonella*. We found that in IFNγ-primed HeLa, GBP1, −2, −3 and −4 coated around 20–30% of intracellular *Salmonella* at 1 h p.i., whereas only very few bacteria were positive for GBP5, −6 or −7 (Fig. 3a, b and Supplementary Fig. 4a). Recruitment of tagged GBP2, −3 and −4 was strongly dependent on IFNγ priming, since these GBPs only poorly associated with *Salmonella* when expressed in naive cells. By contrast, eGFP-GBP1 associated with *Salmonella* even when expressed in naive cells, albeit at lower levels than in primed cells.

GBPs are known to homo- and hetero-oligomerize, forming a coat when recruited to intracellular pathogens^28^. We hypothesized that GBP1 had the ability to target *Salmonella* independently of other GBPs, whereas GBP2-4 required GBP1 for recruitment^27^. Indeed, recruitment of GBP2, −3 and −4 to *Salmonella* was completely abrogated in IFNγ-primed *GBP1*^–/–^ HeLa cells (Fig. 3c). Furthermore, GBP1 co-expression in naive cells was sufficient to induce recruitment of tagged GBP2-4 to intracellular *Salmonella* (Supplementary Fig. 4b, c) to levels similar to those observed in IFNγ-primed cells (Fig. 3b). GBP1 targeting of intracellular *Salmonella* was also observed in primary human small intestinal epithelial cells (Supplementary Fig. 4d). Finally, time-lapse confocal microscopy of infected cells co-expressing GBP1 and either GBP2, −3 or −4, revealed that GBP1 and GBP2 were recruited simultaneously to the bacteria (Supplementary Fig. 4e and Supplementary Movie 3), whereas GBP3 and GBP4 were only recruited to bacteria minutes after GBP1 recruitment (Supplementary Fig. 4f, g and Supplementary Movies 4, 5). These data show that GBP1 is the first to target intracellular *Salmonella* and orchestrates the hierarchical recruitment of additional human GBP family members, namely GBP2-4. To gain further mechanistic insights on how GBP1 accumulates on intracellular *Salmonella* we analyzed different GBP1 mutants (Fig. 3d and Supplementary Fig. 5a, b)^29^. GTP hydrolysis, a triple-arginine polybasic motif (584-586) and the C-terminal CaaX box-dependent farnesylation were all required for proper GBP1 recruitment/accumulation around intracellular *Salmonella* (Fig. 3e and Supplementary Fig. 5c). Interestingly, HA-GBP1^ΔCaaX^ was still not recruited to *Salmonella* even when co-expressed with mCherry-GBP1^wt^ (Fig. 3f), suggesting that GBP1 oligomers around bacteria are only formed if monomers are farnesylated and capable of properly inserting into membranes.

### GBPs target cytosolic *Salmonella* seconds after SCV rupture

**Fig. 4:**
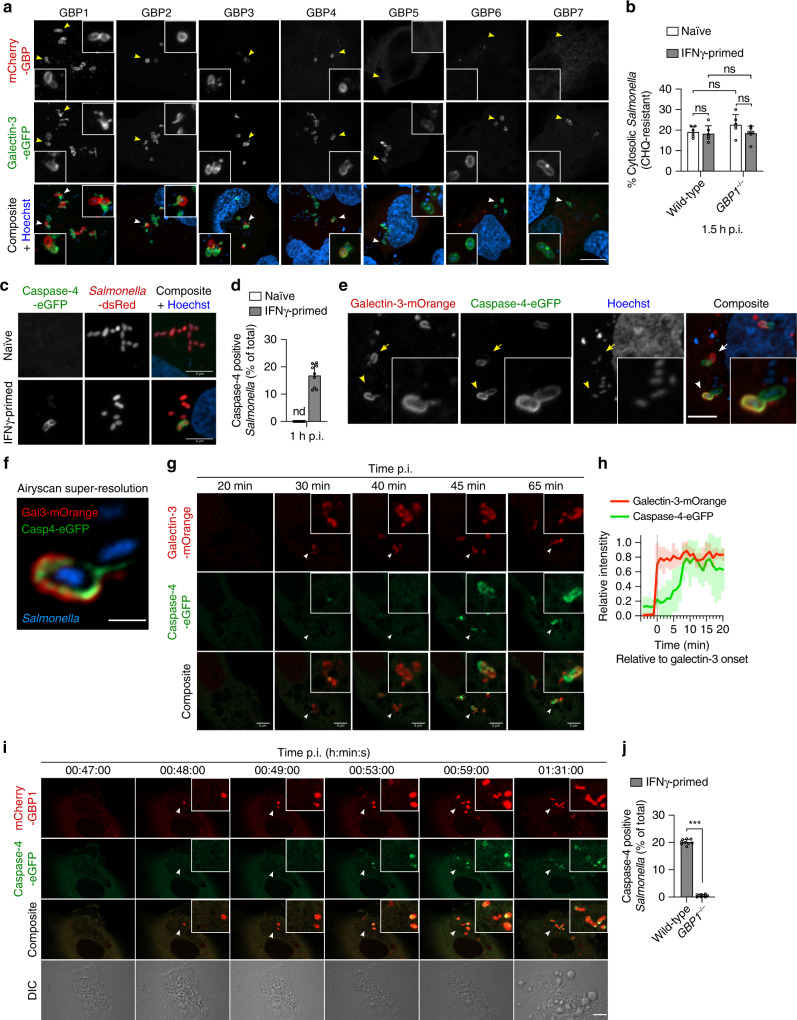
GBP1 targets cytosolic *Salmonella* and is required for caspase-4 recruitment to the bacterial surface. **a** Fluorescence confocal microscopy of IFNγ-primed wild-type HeLa co-expressing galectin-3-eGFP (green) and mCherry-GBP1-7 (red) and infected with *Salmonella* for 1 h. **b** Percentage of CHQ-resistant cytosolic *Salmonella* in naive or IFNγ-primed wild-type or *GBP1*^–/–^ HeLa at 1.5 h p.i. Cells in triplicate wells were infected for 30 min and then treated with gentamicin ± CHQ for an additional 1 h before lysing the cells and determining CFUs. The percentage of cytosolic bacteria was calculated as the ratio of (CHQ + gentamicin^resistant^/gentamicin^resistant^). **c** Fluorescence confocal microscopy of naive or IFNγ-primed HeLa expressing caspase-4-eGFP (green) and infected with *Salmonella*-dsRed for 1 h. **d**. Percentage of caspase-4-eGFP positive *Salmonella* at 1 h p.i., quantified by counting 100–200 bacteria per coverslip. nd, not detected. **e**, **f** Fluorescence confocal microscopy of IFNγ-primed HeLa co-expressing galectin-3-mOrange (red) and caspase-4-eGFP (green) and infected with *Salmonella* for 1 h. **g** Time-lapse fluorescence confocal microscopy of IFNγ-primed HeLa expressing caspase-4-eGFP (green) and galectin-3-mOrange (red) and infected with *Salmonella*. **h**. Mean normalized fluorescence intensities of galectin-3-mOrange and caspase-4-eGFP over time. Fluorescence intensities were quantified in a region of interest as exemplified in the figure, containing an event of caspase-4 and galectin-3 recruitment to an individual bacterium. The relative intensity signals were aligned using the time point of onset of galectin-3 recruitment as zero and the mean and SD of six different events were plotted. **i** Time-lapse fluorescence confocal microscopy of IFNγ-primed HeLa co-expressing caspase-4-eGFP (green) and mCherry-GBP1 (red) and infected with *Salmonella*. Images were acquired every 60 s. DIC, differential interference contrast. **j** Percentage of caspase-4-eGFP positive *Salmonella* at 1 h p.i., in IFNγ-primed wild-type and *GBP1*^–/–^ HeLa. 100–200 bacteria were counted per coverslip. Representative confocal images are shown and scale bars correspond to 1 µm (**f**), 5 µm (**c**, **e**, **g**) or 10 µm (**a**, **i**). Graphs show the mean ± SD, and data are pooled from two (**b**) or three (**d**, **j**) independent experiments performed in triplicate or representative from at least three independent experiments (**a**, **c**, **e**–**i**). ***P < 0.001; ns, not significant, two-tailed *t*-test.

Since mouse GBPs were previously associated with vacuolar rupture^10^, we next asked if human GBPs associate with the SCV or cytosolic *Salmonella* by using galectin-3, a protein that binds to β-galactosides found on the inner leaflet of vacuolar membranes, as a marker for ruptured vacuoles^30^. GBP1-4 were indeed only found in the vicinity of galectin-3-positive bacteria (Fig. 4a), but closer analysis revealed that GBPs targeted the cytosol-exposed part of the bacteria and not the galectin-3-positive ruptured vacuoles (Fig. 4a, insets). Consistently, GBPs did not co-localize with LAMP1 (Supplementary Fig. 6a), a known marker of SCV membranes^31^. Furthermore, we did not observe a reduction in the percentage of cytosolic (Fig. 4b) or galectin-3-positive *Salmonella* (Supplementary Fig. 6b) in *GBP1*^–/–^ cells, indicating that human GBP1-4 did not promote the escape of *Salmonella* from the SCV in HeLa cells, but associate with bacteria upon cytosolic entry. GBP1 also associated with *Shigella flexneri* after its escape from the endocytic vacuole but not with cytosolic *Listeria monocytogenes* (Supplementary Fig. 6c)^27,29^.

We next used time-lapse confocal microscopy to follow the kinetics of SCV membrane rupture, GBP recruitment and pyroptosis. In *Salmonella*-infected cells, SCV rupture and galectin-3 recruitment was followed by a rapid and massive recruitment of GBP1, often occurring less than 30 seconds upon detectable galectin-3 appearance (Supplementary Fig. 6d and Supplementary Movie 6). GBP1 recruitment often started at one region of the bacterium, presumably the part exposed to the cytosol, then formed a coat around bacteria. GBP1 recruitment to cytosolic *Salmonella* was followed by pyroptotic cell death (Supplementary Fig. 6e and Supplementary Movie 7), and consistently, the majority of pyroptotic cells featured GBP1-positive *Salmonella* (Supplementary Fig. 6f).

In mouse macrophages, GBP targeting of bacteria mediates recruitment of Irgb10, which correlates with the lysis of targeted bacteria and caspase-11 activation^11^. To determine if human GBPs lysed cytosolic *Salmonella* in HeLa cells, as a mechanism for LPS release and caspase-4 activation, we monitored GBP recruitment in *GSDMD*^–/–^ cells infected with *Salmonella*-dsRed. Live-cell imaging revealed that while the bacteria were rapidly targeted by GBP1, they continued to divide in the host cell cytosol, showing no signs of lysis or viability loss (Supplementary Fig. 7a–d and Supplementary Movies 8–11). Therefore, GBP1 targets the surface of *Salmonella* within seconds upon rupture of the SCV membrane and bacterial escape to the host cytosol, and is followed by rapid induction of caspase-4-dependent pyroptosis independently of bacteriolysis.

### GBPs control caspase-4 recruitment to cytosolic *Salmonella*

Since GBP recruitment did not lyse bacteria, we presumed that GBPs control LPS-dependent caspase-4 activation by another mechanism. When probing for the intracellular localization of caspase-4 we found that caspase-4-eGFP was recruited on *Salmonella*, often covering the entire bacterium (Fig. 4c). Caspase-4 recruitment onto *Salmonella* absolutely required IFNγ priming as it was not detectable in naive cells (Fig. 4c, d and Supplementary Fig. 8a) despite high expression levels of the caspase. Time-lapse confocal microscopy of caspase-4-eGFP-expressing IFNγ-primed HeLa infected with *Salmonella*-dsRed showed that caspase-4, although initially diffused in the cytosol, was recruited to intracellular *Salmonella* within minutes, and that caspase-4 recruitment to *Salmonella* was rapidly followed by pyroptotic cell death in the majority of cells (Supplementary Fig. 8b, c and Supplementary Movie 12). Consistently, the few cells that did not recruit caspase-4 to bacteria did not initiate pyroptosis (Supplementary Fig. 8d and Supplementary Movie 13).

Recruitment of caspase-4 to *Salmonella* correlated with SCV lysis because caspase-4-positive bacteria were found in the vicinity of galectin-3-positive ruptured vacuoles (Fig. 4e and Supplementary Fig. 8e). Similarly to GBP1-4, caspase-4 did not co-localize with LAMP1-positive SCVs (Supplementary Fig. 8f), but targeted the cytosol-exposed part of the bacterium and not the lysed vacuole (Fig. 4e, arrows and arrowheads, and Supplementary Fig. 8e, inset images). Super-resolution microscopy further confirmed that caspase-4 accumulated on the bacterial surface but not on ruptured SCVs (Fig. 4f and Supplementary Fig. 8g). Finally, time-lapse confocal microscopy of infected cells showed that caspase-4 was recruited to *Salmonella* 5–10 min after the first appearance of a galectin-3 signal (Fig. 4g, h and Supplementary Movie 14), altogether demonstrating that caspase-4 targets cytosolic *Salmonella* after SCV rupture.

Since GBP1 also targeted cytosolic *Salmonella* and was required for caspase-4 activation, we hypothesized that GBPs might either control the recruitment of caspase-4 to cytosolic bacteria and/or the activation of caspase-4 at the bacterial surface. Time-lapse microscopy of HeLa cells co-expressing mCherry-GBP1 and caspase-4-eGFP showed that GBP1 recruitment preceded caspase-4 recruitment to the same bacterium by several minutes, and this was followed by pyroptosis (Fig. 4i and Supplementary Movie 15). This is consistent with the faster recruitment of GBP1 upon SCV rupture compared with the slower recruitment of caspase-4 (Supplementary Fig. 6d and Fig. 4g, h). Remarkably, we also observed a complete reduction in caspase-4 recruitment to *Salmonella* in *GBP1*^–/–^ HeLa (Fig. 4j), while GBP1 was still recruited to bacteria in *CASP4*^–/–^ cells (Supplementary Fig. 9). Together with the observation that *GBP1*-deficient cells were not able to activate caspase-4 (Fig. 2), the data suggest that GBP1, either directly or by controlling GBP2-4 recruitment, initiates the recruitment of caspase-4 to the bacterial surface.

### GBP1/3/4 are sufficient for LPS-induced caspase-4 activation

**Fig. 5:**
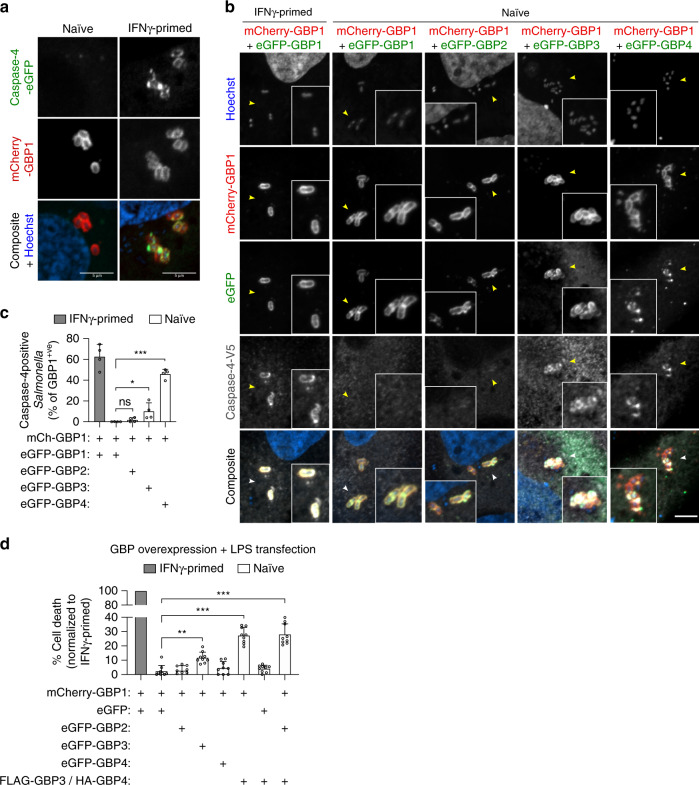
GBP1/4 are sufficient to recruit caspase-4 to *Salmonella* and together with GBP3 activate the non-canonical inflammasome in naive human epithelial cells. **a** Fluorescence confocal microscopy of naive and IFNγ-primed HeLa co-expressing caspase-4-eGFP (green) and mCherry-GBP1 (red) and infected with *Salmonella* for 1 h. DNA was stained with Hoechst (blue). Representative confocal images are shown and scale bar corresponds to 5 µm. **b** Fluorescence confocal microscopy of IFNγ-primed or naive HeLa cells co-expressing mCherry-GBP1 (red), Dox-inducible eGFP-GBP1, −2, −3 or −4 (green) and caspase-4-V5 (gray), and infected with *Salmonella* for 1 h. DNA was stained with Hoechst (blue). eGFP-GBPs were expressed by inducing cells with 1 µg/mL Dox for 16 h. Caspase-4-V5 was visualized by immunostaining with an anti-V5 antibody. Representative confocal images are shown and scale bar corresponds to 10 µm. **c** Percentage of caspase-4-V5 positive *Salmonella* at 1 h p.i., quantified out of the mCherry-GBP1-positive bacteria. At least 50 GBP1-positive bacteria were counted per coverslip. **d** Percentage of cell death in HeLa cells co-expressing constitutive mCherry-GBP1 and Dox-inducible eGFP or eGFP-GBP1, −2, −3 or −4. FLAG-GBP3 and HA-GBP4 were constitutively expressed together using a bicistronic plasmid. Cells were transfected with the indicated plasmids for 24 h. eGFP-GBPs were induced for 16 h with 1 µg/mL Dox, whereas eGFP was induced for 3 h. Cells were then transfected with *E. coli*-derived LPS (2.5 µg/50,000 cells) for 6 h and cell death values were normalized considering IFNγ-primed HeLa as 100% and naive cells co-expressing mCherry-GBP1 and eGFP as 0%. Graphs show the mean ± SD, and data are pooled from two independent experiments performed in duplicate (**c**), pooled from three independent experiments performed in triplicate (**d**) or are representative from two (**b**) or three (**a**) independent experiments. **P < 0.01; ***P < 0.001; one-way ANOVA.

We next addressed the individual functions of GBP1-4 in caspase-4 recruitment and activation. *GBP1*^–/–^ HeLa neither recruit GBP2-4 nor caspase-4 to cytosolic bacteria (Figs. 3c and 4j), thus making it impossible to determine whether GBP1 controls caspase recruitment directly or via other GBPs. We therefore co-expressed caspase-4-eGFP and mCherry-GBP1 in naive or IFNγ-primed cells, and determined caspase targeting to cytosolic *Salmonella*. While GBP1 targeted cytosolic *Salmonella* regardless of IFNγ priming, caspase-4 was only recruited to bacteria in primed cells (Fig. 5a). Thus, other GBPs and/or an unknown IFNγ-induced factor are necessary for GBP1-dependent caspase-4 recruitment. We thus co-expressed caspase-4 and mCherry-GBP1 with either doxycycline (Dox)-inducible eGFP-tagged GBP2, GBP3 or GBP4 in naive HeLa cells and visualized caspase recruitment by confocal microscopy (Supplementary Fig. 10a and Fig. 5b). GBP1 alone, or GBP1 together with GBP2 did not restore caspase-4 recruitment to cytosolic *Salmonella* in naive cells. On the other hand, co-expression of GBP1 with GBP4 and to a lesser degree with GBP3 was sufficient to induce recruitment of caspase-4 to cytosolic bacteria (Fig. 5b, c). The same was observed when using different vectors to co-express the proteins (Supplementary Fig. 10b), which excluded vector-biased caspase-4 recruitment and indicated that GBP1 controls recruitment of caspase-4 mainly via GBP4 and partially via GBP3.

We next asked if expression of single or multiple GBPs in naive cells also restores caspase-4-dependent pyroptosis. Individual expression of GBP1-7 was not sufficient to induce LPS-induced pyroptosis in naive cells (Supplementary Fig. 10c, d). By contrast, LPS transfection induced significantly elevated levels of LDH release in naive cells co-expressing either GBP1/3/4 or GBP1/2/3/4 (Fig. 5d and Supplementary Fig. 10e, f). Interestingly, co-expression of only GBP1/3 already restored some LPS transfection-induced pyroptosis, whereas co-expression of GBP1/4 had no effect even though it was sufficient to promote caspase-4 recruitment on *Salmonella* (Fig. 5b, c and Supplementary Fig. 10b). Thus, while GBP1 drives caspase-4 recruitment to cytosolic *Salmonella* mainly by GBP4, GBP3 is nevertheless necessary to yield caspase-4 activation (Fig. 5b, d). In conclusion, these experiments indicate that a complex formed by GBP1, −3 and −4 promotes caspase-4 recruitment and activation following LPS detection without the requirement for other IFNγ-induced genes.

### GBP1 directly binds LPS

**Fig. 6:**
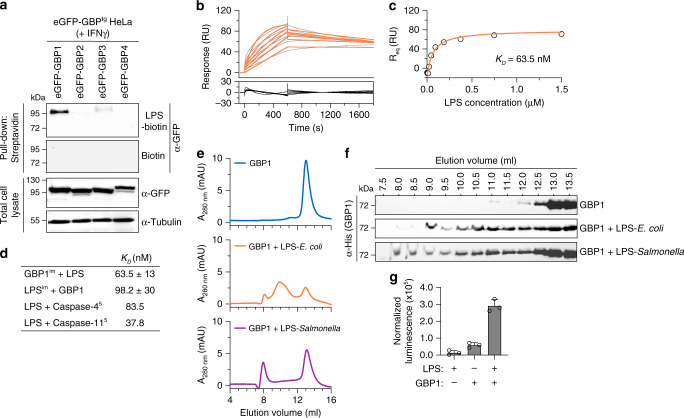
LPS binds to GBP1 to induce formation of a high-molecular weight protein complex. **a** Streptavidin pull-down assay for eGFP-GBP1-4 using biotin or biotin-conjugated LPS. HeLa cells stably expressing Dox-inducible eGFP-GBP1, −2, −3, or −4 were primed with IFNγ and 1 µg/mL Dox was added for 16 h. 1 million cells were lysed and incubated with 2 µg LPS-biotin or biotin, and the biotinylated substrates were pulled down using equal amounts of streptavidin magnetic beads, which were then eluted in equal volumes of SDS-PAGE reducing sample buffer. Streptavidin-bound and -unbound fractions were analyzed by western blot using an antibody against GFP. **b** SPR sensorgram of *E. coli* LPS (O111:B4) binding to human GBP1 immobilized on a CM5 chip surface. Sensorgram was obtained by using different LPS concentrations (47, 94, 188, 375, 750, and 1500 nM). Gray lines correspond to SPR data and orange lines to model fits using a two-state-reaction model. **c** Saturation curve of the titration of LPS on GBP1 immobilized on a CM5 chip. **d** Calculated dissociation constants (*K*_*D*_) for LPS binding to immobilized GBP1 (GBP1^im^) or GBP1 binding to immobilized *E. coli* LPS (LPS^im^). Dissociation constants for LPS-caspase-4 and LPS-caspase-11 were previously published by Shi et al.^5^. **e**, **f** Size exclusion chromatograms of recombinant, LPS-free His-tagged GBP1 incubated with various LPS derivatives. Following purification, GBP1 (1 µM) was incubated on ice with LPS (2 µM) for 5 h before being subjected to size-exclusion analysis on a Superdex 200 10/30 GL column. Protein size was estimated using molecular weight standards. Curves were corrected by subtracting LPS-specific absorbance at 280 nm. Individual fractions were run on a 12% acrylamide gel and immunoblotted against His_6_ to confirm the presence of GBP1 in elution peaks (**f**). **g** GTPase activity analysis of recombinant GBP1. GBP1 (500 nM) was incubated with GTP (5 µM) with or without ultrapure LPS (5 µM) for 30 min before the reaction was stopped. Luminescence was normalized to a buffer-only control. Graphs show the mean ± SD, and data are representative from three (**a**–**d**, **g**) or five (**e**, **f**) independent experiments performed with at least three independently expressed and purified batches of recombinant His-GBP1.

Since GBPs showed a recruitment to cytosolic Gram-negative bacteria, OMVs or even transfected LPS (Fig. 3, Supplementary Fig. 6c and ref. ^12^), we tested if GBPs can directly bind LPS. Similarly to caspase-4 (Fig. 1i and Supplementary Fig. 2g), biotin-LPS was able to pull-down eGFP-GBP1 and to a lesser degree eGFP-GBP3, but not tagged GBP2 or GBP4 (Fig. 6a) from HeLa lysates. To assess if this interaction was direct, we next purified LPS-free recombinant His-GBP1 from CleanColi® BL21 (DE3) bacteria that produce Lipid IV_A_ (which does not activate caspase-4/−11) instead of LPS followed by a lipid removal protocol^32^ and tested GBP1-LPS binding by surface plasmon resonance (SPR). SPR showed a direct binding of LPS to immobilized GBP1 with a *K*_*D*_ of ~60 nM, which is comparable to the published *K*_*D*_ of the LPS-caspase-4 and LPS-caspase-11 interaction (Fig. 6b–d). Kinetic analysis also showed that GBP1-LPS binding best fitted with a two-state-reaction model that describes a situation where initial binding is followed by a conformational change that stabilizes the complex. The complementary experiment with immobilized LPS also yielded similar a *K*_*D*_ (Supplementary Fig. 11a, b), however the response was relatively weak since only a small amount of LPS absorbed on the chip surface. Moreover, microscale thermophoresis (MST) of GBP1 and FITC-LPS confirmed the interaction yielding a comparable *K*_*D*_ value (Supplementary Fig. 11c).

We next investigated the consequences of the LPS-GBP1 interaction, since LPS was suggested to induce caspase-11/−4 oligomerization^5^. LPS-free recombinant GBP1 ran as a single monomeric peak on size-exclusion chromatography (SEC), close to its predicted size of 68.5 kDa (Fig. 6e, f, Supplementary Fig. 11d and Table 1), while *E. coli* and *Salmonella* Typhimurium LPS formed micelles eluting at around 1000 kDa (void volume). When GBP1 was incubated with LPS, the elution profile changed, resulting in a shift of the majority of GBP1 to higher molecular weight peaks from the range of 400 kDa to over 1000 kDa (Fig. 6e, f, Supplementary Fig. 11d and Table 1). The most prominent peak was found to be at 1000 kDa, indicating that GBP1 bound to LPS micelles, but smaller complexes were detected as well. Interestingly, even LPS from *Rhodobacter sphaeroides* induced a shift of GBP1 to higher molecular weight peaks (Supplementary Fig. 11d, e), despite acting as an antagonist of caspase-11 and not being able to induce oligomerization of the caspase^5^. Conversely, incubation of GBP1 with lipoteichoic acid (LTA) or peptidoglycan (Supplementary Fig. 11f, g) did not result in a similar shift. LBP and ovalbumin were used as positive and negative LPS-binding controls, respectively (Supplementary Fig. 12a)^33^. We next tested what part of LPS is required for the binding of GBP1 to LPS micelles. LPS from *E. coli* Ra, Rc, Rd, Re mutant strains, which lack the O-antigen and outer core, respectively, was still bound by GBP1 as it resulted in a shift to high-molecular weight peaks (Supplementary Fig. 12b, c). Consistently, *Salmonella* Δ*waaL* or Δ*waaG* mutants (lacking the O-antigen or outer core, respectively) still recruited GBP1 and caspase-4 in infected cells (Supplementary Fig. 12d–f). Since the LPS-GBP1 interaction was highly sensitive to detergents, the binding to Lipid A could not be tested since this requires solubilization of Lipid A with Tween-20 or other detergents^5^. Since GBP1 displays an oligomerization-dependent activation of GTP hydrolysis^34^, we finally assessed the impact of LPS on GBP1 GTPase activity (Fig. 6g). As reported previously, GBP1 had some intrinsic ability to hydrolyze GTP on its own, but its GTPase activity was significantly increased when incubated with LPS, supporting the fact that GBP1 interacts with LPS, and suggesting that LPS binding might promote an oligomeric state. In conclusion these findings indicated that GBP1 was able to bind directly to LPS, and that the LPS Lipid A and inner core region were sufficient for LPS-GBP1 interaction.

**Table 1:** Observed molecular weights of GBP1 peaks after incubation with various LPS.

Sample	Theoretical size (kDa)	Observed size (kDa)
GBP1	67.01	78.7
GBP1 + LPS-*E. coli*		1028; 401.8; 78.7
GBP1 + LPS-*Salmonella*		1140.5, 78.7
GBP1 + LPS-*R. sph*		1318.6; 364

### GBP1-LPS interaction involves electrostatic interactions

**Fig. 7:**
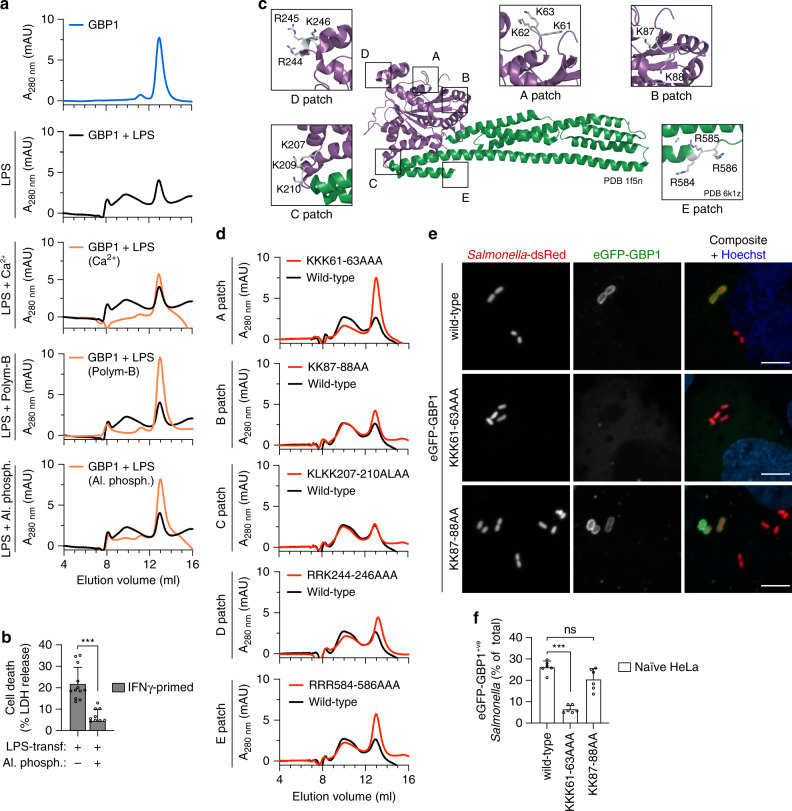
GBP1 is recruited to the bacterial surface and binds LPS through electrostatic interactions. **a** Size exclusion chromatograms of recombinant His-tagged GBP1 incubated with *E. coli* LPS, or with *E. coli* LPS pre-treated with CaCl_2_ (5 mM), Polymyxin B (10 µg/mL) or with alkaline phosphatase. Curves were corrected by subtracting the respective LPS-specific absorbance at 280 nm. Black curves representing control condition were overlayed. **b** Release of LDH from IFNγ-primed HeLa 5 h after transfection with *E. coli* LPS, or after transfection with LPS previously treated with alkaline phosphatase. **c** 3D structure of human GBP1 (PDB 1f5n), highlighting five different negatively charged patches (A to E). Residues comprising patch E are only visible in PDB 6k1z. For each patch, the indicated residues were all mutated to alanines and analyzed for GBP1-LPS interaction by size exclusion chromatography. Purple indicates GTPase domain, green indicates helical domain. **d** Size exclusion chromatograms of different His-tagged GBP1 mutants incubated with *E. coli* LPS. Curves were corrected by subtracting LPS-specific absorbance at 280 nm. **e** Fluorescence confocal microscopy of naive HeLa expressing eGFP-GBP1^wt^, eGFP-GBP1^KKK61-63AAA^ or eGFP-GBP1^KK87-88AA^ and infected with *Salmonella*-dsRed for 1 h. DNA was stained with Hoechst (blue). Representative confocal images are shown and scale bar corresponds to 5 µm. **f** Percentage of eGFP-GBP1 positive *Salmonella* at 1 h p.i., as quantified by counting between 100–200 bacteria per coverslip. Graphs show the mean ± SD, and data are pooled from three independent experiments performed in duplicate (**f**), four independent experiments performed in triplicate (**b**), or are representative from three (**a**, **d**, **e**) independent experiments. ***P < 0.001; ns, not significant, two-tailed *t*-test.

Each LPS molecule from *E. coli* has 6–8 negatively charged groups, from phosphates and acid groups in the Lipid A and inner core (Supplementary Fig. 12b)^35^. Given that human GBP1 lacks the hydrophobic pockets that comprise the LPS binding sites in CD14 and MD-2, we hypothesized that GBP1 binds to LPS by electrostatic interactions, similarly to LBP^36,37^. Consistently, GBP1 binding to LPS micelles was disrupted by incubation with cations (Ca^2+^), which neutralize the negative charges on LPS^35,38^, or by incubation with polymyxin B, which interacts with the LPS Lipid A and inner core region through ionic and hydrophobic forces, resulting in more monomeric GBP1 and reduced levels of GBP1-LPS association (Fig. 7a and Supplementary Fig. 13). Furthermore, dephosphorylating LPS with alkaline phosphatase reduced GBP1-LPS binding and cell death upon LPS transfection (Fig. 7a, b and Supplementary Fig. 13), indicating that the phosphate groups found on Lipid A and inner core sugars of LPS (Supplementary Fig. 12b) play an essential role in promoting GBP1-LPS interaction and subsequent activation of the non-canonical inflammasome pathway. Basic residues in the N-terminal domain of LBP mediate binding to LPS micelles^39^. We thus mutated several positively charged surface patches in GBP1 and tested the impact of the mutations on GBP1-LPS interaction by SEC (Fig. 7c, d). While most mutations resulted in no or minor effect on the binding of GBP1 to LPS micelles, mutation of the triple-lysines 61-63 to alanines (A patch) notably reduced the formation of the higher molecular weight GBP1 peaks (~40%) and increasing the levels of monomeric GBP1, thus suggesting that these residues are required for binding (Fig. 7d and Supplementary Fig. 14a). Furthermore, expression of GBP1^KKK61-63AAA^ (A patch) resulted in a significant reduction of targeting to cytosolic *Salmonella* compared with either GBP1^WT^ or GBP1^KK87-88AA^ (B patch) (Fig. 7e, f and Supplementary Fig. 14b). In summary, the results demonstrate that the GBP1-LPS interaction involves electrostatic forces and that disrupting the binding by dephosphorylating LPS or mutating GBP1 results in reduced caspase-4-induced pyroptosis upon LPS transfection or impaired GBP1 targeting of the bacterial surface.

## Discussion

Here we report that GBP1 functions as an LPS sensor that recognizes Gram-negative bacteria in the cytosol of human epithelial cells, and that GBP1-LPS interaction involves electrostatic forces (Supplementary Fig. 15). Given that GBP1 is necessary for LPS-induced caspase-4 activation in various human cell types and after various LPS delivery methods (electroporation, chemical transfection, Gram-negative bacteria infection), our data imply that GBP1 is the very first protein in the non-canonical inflammasome pathway that interacts with LPS. This places GBP1 upstream of caspase-4 in cytosolic LPS sensing, raising the question if it might act similarly to LBP which acts as a co-factor for extracellular LPS detection by CD14 and MD-2/TLR4. Indeed, the finding that GBP1-LPS requires negative charges on LPS Lipid A and the inner core sugars as well as a positively charged surface patch in GBP1 is reminiscent of the mode of LPS binding by LBP, which involves two positively charged patches at the tip of the LBP N-terminal domain. From a functional point of view LBP and GBP1 will most likely differ. LBP binds to LPS micelles and to CD14 protein, and catalyzes multiple rounds of LPS transfer to CD14, which will then transfer a single bound LPS molecule to MD-2/TLR4. GBP1 on the other hand, does not function alone, but as part of a GBP1-4 complex that assembles on LPS-containing membranes. It is possible that this complex recruits caspase-4 and then transfers LPS onto caspase-4 thus promoting its activation. Alternatively, it is also possible that the assembly of the GBP complex and insertion of the GBPs into the bacterial membranes via lipid anchors results in a partial weakening of membrane integrity, thus allowing caspase-4 to bind the Lipid A moiety of LPS. Additional studies aimed at determining the structure and composition of the GBP complex will be necessary to understand how it promotes caspase-4 recruitment and activation.

While it is not yet clear if the GBP1-LPS interaction also requires structural determinants in LPS, the ability of GBP1 to recognize negatively charged pathogen-derived molecules might extend its function beyond the recognition of Gram-negative bacteria. Indeed, GBP1 is known to be recruited to both the surface of cytosolic parasites, such as *T. gondii*, as well as to the membrane of the *T. gondii* parasitophorous vacuole, and to assemble a GBP coat in a similar manner^25,28^. Interestingly, in this case the GBP coat does not result in recruitment of caspase-4 (in line with the fact that parasites do not feature LPS), but in the induction of caspase-8-dependent apoptosis^40^. It is thus likely that parasites or their vacuoles feature molecules with similar chemical properties as the LPS Lipid A and core polysaccharides in their cell membrane or the membrane of the parasitophorous vacuole. Identification of additional ligands that bind GBP1 or other GBP family member will enhance our understanding of host innate immunity and establish new paradigms for pattern recognition.

The human-specific mechanism reported here is in contrast to previous models that proposed that, in mice, GBPs promote non-canonical inflammasome activation by facilitating vacuolar escape and inducing bacterial membrane destruction^10,41^. While it is unlikely that mouse GBPs function fundamentally differently from human GBPs in the mechanism by which they recognize pathogens, it is possible that the existence of IRGs in mouse enhances their downstream effector functions. The lysis of bacteria and vacuoles reported in mouse cells (but not detected in human cells), is most likely a consequence of GBP-mediated recruitment of IRGs, such as Irgb10 that is reported to have antimicrobial properties^11^. Thus, in addition to recruiting caspase-11 directly in analogy to human GBPs, mouse GBPs might also mediate access to LPS and LPS liberation through the membranolytic activity of IRGs.

To our knowledge, our study is the first to report a ligand of GBP1 and to characterize the mode of this interaction. While our findings still need to be validated in primary human cells, it nevertheless provides the first evidence that GBP1 and possibly other GBPs function as direct innate immune receptors for pathogen-associated molecular patterns, expanding the ever-increasing repertoire of cytosolic innate immune defense pathways.

## Methods

### Bacterial strains and mammalian cell culture

All bacteria were grown at 37 °C in an orbital shaker. *Salmonella enterica* serovar Typhimurium strain SL1344 was grown in lysogeny broth (LB) medium supplemented with 10 g/L NaCl and streptomycin (50 µg/mL). *Salmonella* expressing dsRed (*Salmonella*-dsRed) was grown by supplementing LB medium with ampicillin (50 µg/mL). *Salmonella enterica* serovar Typhimurium strain 4/74 and their isogenic Δ*waaG* or Δ*waaL* mutants were a kind gift from Jay Hinton (University of Liverpool, Liverpool). *Shigella flexneri* M90T expressing the adhesin AfaI was a kind gift from Jost Enninga (Institut Pasteur, Paris) and was grown in tryptic soy broth (TSB) supplemented with ampicillin (50 µg/mL). *Listeria monocytogenes* strain EGD was a kind gift from Pascale Cossart (Institut Pasteur, Paris) and was grown in brain-heart infusion (BHI) medium. Unless stated otherwise, the HeLa clone CCL-2 from ATCC was used. Human epithelial HT-29 and HeLa (CCL-2 or Kyoto clones) cells were cultured in DMEM (Gibco) supplemented with 10% Fetal Calf Serum (FCS). Caco-2/TC-7 were cultured in DMEM supplemented with 20% FCS. HT-29 and Caco-2 cells were a kind gift from Shaynoor Dramsi (Institut Pasteur, Paris). THP-1 and U937 cells were cultured in RPMI supplemented with 10% FCS. HBEC3-KT cells were obtained from ATCC and were grown in Bronchial/Tracheal Epithelial Cell Growth Medium (Cell Applications, Inc.). HaCaT cells were obtained from CLS Cell Lines Service GmbH, and were grown in DMEM supplemented 10% FCS. HIEC-6 cells were obtained from ATCC and grown in Opti-MEM (Gibco) supplemented with 4% FCS, 10 mM Glutamine and 10 ng/mL of epidermal growth factor (EGF). Human primary monocyte-derived macrophages (hMDMs) were purified from buffy-coat obtained from the Swiss Red-Cross and purified and cultured as described previously^42^. All cells were grown at 37 °C, 5% CO_2_.

### Generation of CRISPR/Cas9 knockout cell lines

Knock-out HeLa cells lines were generated using the Alt-R CRISPR-Cas9 System (Integrated DNA Technologies, IDT), by using a mix of a sequence-specific CRISPR RNA (crRNA), a conserved, transactivating crRNA (tracrRNA) and recombinant Alt-R *S. pyogenes* Cas9 (IDT). crRNA and tracrRNA were mixed to 1 µM, heated 5 min at 95 °C and cooled to room temperature. 1 µM Alt-R Cas9 was mixed and incubated at room temperature for 5 min. Lipofectamine RNAiMax transfection reagent (Invitrogen) was then added and the mixture was incubated for 20 min at room temperature. 40,000 cells/well were reversely transfected with the previous mixture in 96-well plates, to achieve a concentration of 10 nM ribonucleoprotein complex. After incubation for 2 days at 37 °C, 5% CO_2_, single clones were generated by serial dilutions and the desired gene knockouts were screened by performing the T7 endonuclease I assay, verified by sequencing of the PCR fragments and confirmed by western blotting. The following crRNAs were used: AGGGATTCCAACACCTTAAG (for *CASP4*), CCACGTACACGTTGTCCCCG (for *GSDMD*) and GAACACTAATGGGCGACTGA (for *GBP1*).

### Plasmids, siRNAs, and cell transfection

Plasmids expressing N-terminal fluorescently tagged GBPs were generated by inserting the GBPs coding sequences at the XhoI/HindIII sites of pEGFP-C1 and pmCherry-C1 (Clontech). pmiRFP703-GBP1 was generated by using the pEGFP-C1 plasmid and replacing eGFP by miRFP703 (addgene 80001^43^ was used as PCR amplification template) at the NheI/BglII sites, and then inserting the GBP1 coding sequence at the XhoI/HindIII sites. Caspase-4-eGFP was generated by fusing the amplified PCR products of caspase-4 and eGFP and inserting the coding sequence into the NheI/HindIII sites of pEGFP-C1. The pAIP vectors expressing HA-GBP1 or HA-GBP2 were a kind gift from T. Henry (CIRI, Lyon) and were used to generate HA-tagged GBPs, by replacing GBP1 by the GBP3 or −4 coding sequences at the EcoRI sites. The bicistronic plasmids encoding FLAG-GBP3 + HA-GBP4 were generated by inserting GBP3 or GBP4 at the NotI/PmeI sites of the pBud-EGFP vector (addgene 23027)^44^. Doxycycline-inducible eGFP-GBP1, −2, −3, −4 were generated by amplifying eGFP-GBPs generated above by PCR and inserting the coding sequences at the BamHI site of the pLVX-Puro vector (Clontech). All cloning was performed using In-Fusion cloning technology (Clontech) and plasmids were verified by sequencing. Plasmids encoding eGFP or mOrange tagged galectin-3 were a kind gift from Jost Enninga^30,45^. HeLa cells were either plated onto 8-well µ-slides (Ibidi) at a density of 1.5 × 10^4^ cells/well for live imaging, onto 24-well plates containing glass coverslips at a density of 1.0 × 10^5^ cells/well, onto 96-well glass bottom plates (Greiner) or onto 96-well plates (Eppendorf) at a density of 8.0 × 10^4^ cells/well 24 h before transfection. Cells were then transfected with one, two or three expression plasmids using X-tremeGENE 9 DNA transfection reagent (Roche) for 16–48 h, according to the manufacturer’s instructions. A list of the plasmids and primers used in this study is provided in Supplementary Table 1. For siRNA knock-down experiments, cells were seeded onto 96-well plates at a density of 9.0 × 10^4^ cells/well and on the following day transfected with 3 pmol (25 nM) Stealth RNAi^TM^ siRNAs (Thermo Fisher Scientific) using Lipofectamine RNAiMax (a list of the siRNAs used in this study is shown in Supplementary Table 2). After 8 h, cells were incubated with IFNγ for an additional 16 h and experiments were then performed.

### Infection assays and transfection of cells with LPS

When indicated, cells were primed with 10 ng/mL human IFNγ (Peprotech) for 16 hours. Overnight *Salmonella* cultures were sub-cultured 1/50 and grown until late exponential/early stationary phase (OD_600_ = 1.5–1.8). Overnight *Shigella* or *Listeria* cultures were sub-cultured 1/100 and grown until mid-exponential phase (OD_600_ = 0.5–0.7). Before infection, bacteria were collected by centrifugation, gently washed and resuspended in DMEM. *Salmonella* was added to HeLa cells in 96-well plates (approximately 50,000 cells per well) at a multiplicity of infection (MOI) of 50 and incubated for 30 min at 37 °C. For infection with *Shigella* or *Listeria*, bacteria were added to cells at a MOI of 20 and incubated for 30 min at 37 °C. Non-internalized bacteria were removed by three washes with warm DMEM and cells were incubated with DMEM containing 100 µg/mL gentamicin for 1 h to kill extracellular bacteria. Medium was then changed to DMEM containing 10 µg/mL gentamicin and 10% FCS for the remained of the experiment. At the desired time points p.i., cells were either processed for LDH release, enumeration of intracellular bacteria or fixed for immunofluorescence assays. To enumerate intracellular bacteria, infected cells were gently washed with PBS and lysed with water containing 0.2% Triton X-100. Bacteria were then serially diluted and plated onto LB agar. To quantify the percentage of cytosolic *Salmonella* in the total population, we used a CHQ resistance assay. Briefly, infected cells were incubated with 200 µg/mL CHQ (Sigma-Aldrich) and gentamicin for 1 h (CHQ-resistant bacteria) or with gentamicin only (total bacteria). Cells were washed, lysed and bacteria were plated as described above. The percentage of cytosolic bacteria was calculated by the ratio of (CHQ + gentamicin^resistant^/gentamicin^resistant^). U937 and THP-1 cells were seeded and differentiated with 100 ng/mL PMA for 48 h, followed by a 24 h resting period.

Transfection of cells with smooth LPS from *E. coli* O111:B4 (Invivogen) or *Salmonella* (Sigma, L6143) was done at a concentration of 2.5 µg/50,000 cells, or 2.5 µg/80,000 cells (for THP-1 or U937), using Lipofectamine 2000 (Invitrogen). Briefly, LPS was diluted in Opti-MEM and incubated with Lipofectamine 2000 (1.0 µl/50,000 cells) for 20 min at room temperature. 75 µl of Opti-MEM was added to cells on 96-well plates and then 75 µl of LPS mixture was added on top. Plates were centrifuged for 5 min at 211 × *g* and then incubated at 37 °C for the indicated time points. For electroporation of HeLa or HBEC3-KT cells, the Neon Transfection System (Life Technologies) was used. Briefly, naive or IFNγ-primed cells were harvested, resuspended in resuspension buffer T and electroporated with LPS from *E. coli* O111:B4 at a concentration of 300 ng/50,0000 cells, using electrolytic buffer E and 1 pulse of 1300 V for 20 ms. Cells were then added to 200 µl pre-warmed Opti-MEM in a 96-well plate, centrifuged for 5 min at 211 × *g* and then incubated at 37 °C. Mock electroporation and non-electroporated cells were used as controls.

### Microscopy, time-lapse imaging, and image analysis

Infected cells were washed once with PBS and fixed in 4% PFA for 20 min. Cells were then washed three times, permeabilized with 0.05% saponin and blocked with 1% BSA. Coverslips were incubated with antibodies when indicated and with Hoechst (1:1000) in PBS, and then mounted in ProLong Gold Antifade (Life Technologies) for confocal microscopy. Samples were imaged with a Zeiss LSM800 confocal laser scanning microscope using a 63×/1.4 NA oil objective, by acquiring Z-stacks of 300 nm step size. For live imaging, infection assays were performed in EM buffer (120 mM NaCl, 7 mM KCl, 1.8 mM CaCl_2_, 0.8 mM MgCl_2_, 5 mM glucose, 25 mM HEPES, pH 7.3). Cells were infected for 10 min as previously described, extracellular bacteria were removed by washing with warm EM buffer, and time-lapse microscopy of living cells was performed at 37 °C using a motorized xyz stage with autofocus. Super-resolution was performed using the Zeiss LSM800 Airyscan super-resolution system using the same objective and super-resolution images were calculated using the Zeiss ZEN software. Data were further analyzed and processed using FiJi software, and all fluorescence derived images shown here correspond to maximum 3D projections.

### LDH release, PI uptake, IL-18 release, and western blotting

Cell death was quantified by measuring LDH release to the supernatant, using the LDH cytotoxicity detection kit (Takara, Clontech). To normalize for spontaneous cell lysis, the percentage of cell death was calculated as follows: (LDH_sample_ – LDH_negative control_)/(LDH_positive control_ – LDH_negative control_) × 100. PI influx measurement was performed as previously described^46^. The levels of IL-18 were measured by ELISA (R&D Sytems), according to the manufacturer’s instructions. For western blotting analysis, cell lysates were prepared and supernatants were precipitated. Mouse anti-caspase-4 4B9 (ADI-AAM-114-E, Enzo Life Sciences, 1:750), rabbit anti-GSDMD (ab210070, abcam, 1:1000), rabbit anti-GBP1 (ab121039, abcam, 1:1000), mouse anti-GAPDH (AM4300, Thermo Scientific, 1:1000), mouse anti-V5 (R960-25, Thermo Scientific, 1:2000), mouse anti-GFP (632381, Clonetech, clone JL-8, 1:5000), mouse anti-HA (ENZ-ABS-118-0200, Enzo Life Sciences, 1:2000), mouse anti-tubulin (ab40742, Abcam, 1:2000) were used and detected with horseradish peroxidase-conjugated secondary antibodies (1:5000, Southern Biotech).

### Active caspase pull-down

HeLa cells were seeded onto 6-well plates and primed for 16 h with 10 ng/mL human IFNγ. Approximately 3 × 10^6^ cells were then treated with 10 µM of biotin-VAD-fmk and transfected with 20 µg of *E. coli* LPS for 3 hours. Cells were lysed and incubated overnight with 20 µl of pre-washed streptavidin magnetic beads (Thermo Scientific). The beads were washed as described elsewhere^47^ and streptavidin-bound and left-over fractions (unbound) were analyzed on a 12% acrylamide gel and blotted against caspase-4.

### Streptavidin pull-down assays

Approximately 2 × 10^6^ cells were collected and lysed in pull-down buffer (50 mM Tris-HCl (pH 7.5), 150 mM NaCl, 5 mM EDTA, 1% NP40, 0.05% Na-deoxycolate and complete protease inhibitors). 700 µg of protein (as determined by BCA assay (Thermo Scientific) from the total cell lysate was incubated with 2 µg biotinylated LPS or with biotinylated Pam_3_CSK_4_ (Invivogen) at room temperature for two hours with rocking. After incubation, 20 µl of pre-washed streptavidin magnetic beads (Thermo Scientific) were added and incubated for 1 h at room temperature with constant rocking. The beads were washed three times in PBS with 0.05% Tween-20 and once with PBS and the precipitates were eluted in equal volumes of SDS-PAGE reducing sample buffer followed by western blotting analysis. 5% the of initial cell lysate (input) and equal volumes of pull-down were analyzed. For GFP-GBP pulldown assay, cells were lysed at a concentration of 20 × 10^6^ cells/mL of lysis buffer (50 mM Tris pH 7.4, 150 mM NaCl, 10 mM MgCl2, 5 mM GTP, 300 μM AlF, 100 μg/mL digitonin (Sigma) and allowed to lyse on ice for 15 min. The cells were then spun 15 min at 6000 × *g*, 4 °C. The soluble extract was then incubated with 2 µg LPS/million cells equivalent and incubated at RT for 2 h with rotation. After incubation, streptavidin magnetic beads were added to the mix and incubated for an additional hour with rotation. The beads were than washed three times (30 min wash) with lysis buffer. The beads where than resuspended in reducing western blot loading buffer before being analyzed by immunoblotting.

### Quantitative PCR (qPCR)

Total mRNA was extracted from HeLa cells using the RNeasy Mini kit (Qiagen) and up to 400 ng were reversed transcribed into cDNA using the Verso cDNA Synthesis kit (Thermo Fisher Scientific). Gene expression levels were quantified by qPCR using a LightCycler 480 (Roche) and LightCycler 480 SYBR Green I Master (Roche), according to standard protocols, by normalizing each sample to the respective levels of the housekeeping mRNA *HPRT*. The list of primers used for qPCR is shown in Supplementary Table 3.

### Purification of recombinant proteins

Full-length human GBP1 was cloned in pET-28a to generate an N-terminally His-tagged hGBP1 construct. pET-28a-hGBP1 was transformed into CleanColi BL21 (Lucigen), and the bacteria were grown in 2xYT medium until an OD_600_ of 0.5-0.7. Protein expression was then induced at 30 °C for 5 h with 0.2 mM IPTG. The bacterial pellet was resuspended in resuspension buffer (50 mM Tris pH 7.4, 150 mM NaCl, 1% Tween 20) and frozen at −80 °C until purification. For most assay, protein was freshly purified on a Ni-NTA affinity column using standard protocols^48^. Protein yield was quantified using Beer-Lambert law. After purification on a Ni-NTA columns, GBP1 was further purified on a size exclusion chromatography column (Superdex 200 10/30 GL, GE Healthcare) in running buffer (50 mM Tris pH 7.4, 150 mM NaCl) and concentrated using Amicon Ultra4 10 kDa (Millipore).

### Size exclusion chromatography of GBP1 and LPS

Freshly purified GBP1 (1 µM) was incubated on ice alone or with a two-fold molar excess of LPS or LPS-derivatives for 5 h. Ultrapure O11:B4 *E.coli* LPS (Invivogen), *Salmonella* Typhimurium Smooth LPS (Enzo Life Science), *Rhodobacter sphaeroides* ultrapure LPS (Invivogen), *E. coli* F585 diphosphoryl Lipid A (Sigma-Aldrich), *Salmonella minnesota* 595 Lipid A (Invivogen), synthetic monophosphorylated Lipid A (Invivogen), *E.coli* EH100 LPS Ra mutant (Sigma-Aldrich), *E.coli* J5 LPS Rc mutant (Sigma-Aldrich), *E.coli* F583 LPS Rd mutant (Sigma-Aldrich), *E. coli* R515 LPS Re mutant (Enzo Life Science) was used. After incubation, GBP1 alone or GBP-LPS incubations were injected into a Superdex 200 10/30 GL column and run in running buffer for 1 column volume. Individual fractions (500 µL), were collected, precipitated with methanol and chloroform^49^, separated on a 12% acrylamide gel and analyzed by immunoblotting using an antibody against His_6_ tag. Experimental molecular weights of the peaks were approximated using a gel filtration standard (1511901; Bio-Rad). Where indicated, LPS was pre-incubated with 5 mM CaCl_2_ for 5 min on ice before being added to GBP1, as indicated above, or LPS was pre-incubated with polymyxin B (10 µg/mL) for 5 min at room temperature.

### Surface plasmon resonance (SPR)

SPR measurements were performed on the Biacore T200 (GE Healthcare Life Sciences). GPB1 was immobilized on a CM5 sensor chip (GE Healthcare) using the amine coupling procedure (immobilization response was 310 RU or 0.31 ng/mm^2^). Then it was equilibrated in PBS buffer (pH 7.2; Gibco, Life Sciences), followed by the injection of the increasing concentrations of LPS (47, 94, 188, 375, 750, 1500 nM) into the flow channels. In the reverse experiment, when immobilizing LPS on the CM5 sensor chip, increasing concentrations of GBP1 (21.5, 43, 86, 172, 343, 688, 1375, 2750 nM) were used. Data were analyzed using BiacoreT200 Evaluation software 3.0. An equilibrium analysis was done using Langmuir isotherm fit with one equilibrium dissociation constant (*K*_*D*_). The best fit for the Kinetic curves was obtained with the two-state-reaction model that assume a possible structural re-arrangement after the initial binding.

### Microscale thermophoresis (MST)

MTS was performed on 50 nM of FITC-labeled *E. coli* LPS (Sigma-Aldrich) using freshly purified hGBP1 expressed recombinantly (as described above) or BSA as a control. Serial dilutions of GBP1 or BSA were analyzed in assay buffer (50 mM Tris pH 7.4, 150 mM NaCl). Experiment was performed on a Nanotemper Monolith NT.115 microscale electrophoresis instrument with medium MST power. Data were fitted to a 1:1 binding model with the MO.Affinity Analysis software.

### GTPase activity assay

GTPase activity assay was performed using the GTPase-Glo^TM^ kit (Promega) according to the manufacturer use. Recombinant human GBP1 (500 nM) was incubated with 5 µM LPS  in GEF buffer (Promega) for 30 min at room temperature before assessing GTP hydrolysis. Luminescence values were normalized to a no-GBP1 control.

### Data analysis

Data analysis was performed using the following software: Gen5, GraphPad Prism v8 and Microsoft Excel. Statistical significances are referred as *, ** or *** for *P*-values <0.05, <0.01 or <0.001, respectively. For comparison of two groups, a two-tailed *t*-test was used, whereas for comparison of three or more groups *P*-values were determined using the two-way analysis of variance for multiple comparisons.

### Reporting summary

Further information on research design is available in the Nature Research Reporting Summary linked to this article.

## Supplementary information

Supplementary Information

Peer Review File

Description of Additional Supplementary Information

Supplementary Movie 1

Supplementary Movie 2

Supplementary Movie 3

Supplementary Movie 4

Supplementary Movie 5

Supplementary Movie 6

Supplementary Movie 7

Supplementary Movie 8

Supplementary Movie 9

Supplementary Movie 10

Supplementary Movie 11

Supplementary Movie 12

Supplementary Movie 13

Supplementary Movie 14

Supplementary Movie 15

Reporting Summary

## Data Availability

The source data corresponding to Figs. 1h–i; 2a, d, e; 6a, f and Supplementary Figs. 1g; 2c, f–h; 3q; 5b; 10d, f; 11e; 14b are provided as Source Data files. All other relevant data are available from the corresponding author upon reasonable request. Source data are provided with this paper.

## Source data

Source Data
