# Supplementary material for: Human GBP1 binds LPS to initiate assembly of a caspase-4 activating platform on cytosolic bacteria
Source: Nat Commun. 2020 Jun 24;11:3276. doi: 10.1038/s41467-020-16889-z (PMC7314798; doi:10.1038/s41467-020-16889-z)
Supplement: Supplementary file 1 — Supplementary Information [file 41467_2020_16889_MOESM1_ESM.pdf]

Supplementary Material for

Human GBP1 binds LPS to initiate assembly of a caspase-4 activating platform on cytosolic bacteria

Santos *et al*.

Supplementary Figure 1

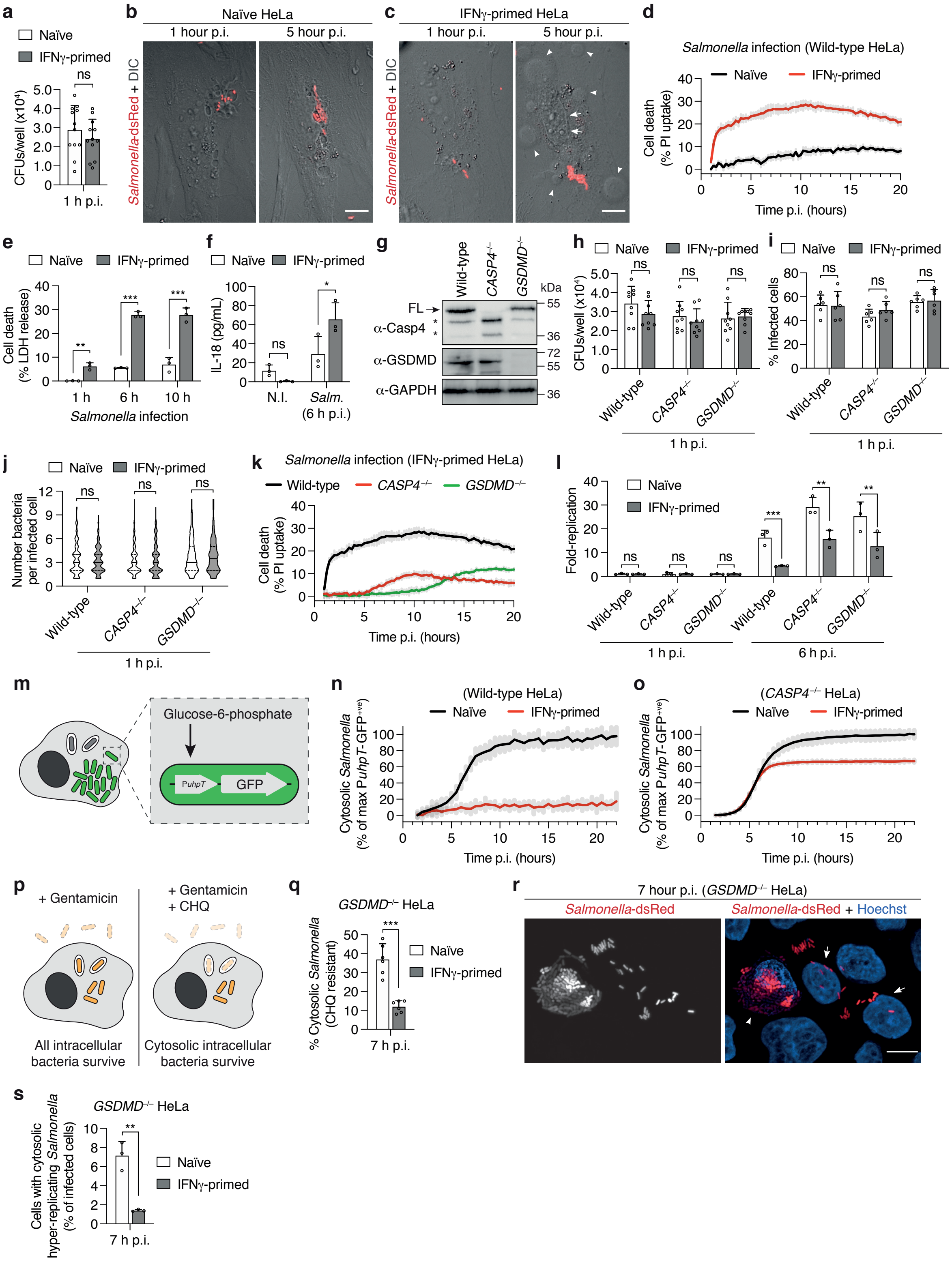

Supplementary Fig. 1. IFNγ priming of HeLa cells induces caspase-4 activation upon *Salmonella* infection and restricts cytosolic bacterial hyper-replication.

a. Assessment of *Salmonella* invasion at 1 h post-infection (p.i.), in naïve or IFNγ-primed HeLa. Cells were infected for 30 min, washed and gentamicin was added to kill extracellular bacteria. The cells were then lysed and the number of viable intracellular bacteria was counted by CFUs.

b, c. Confocal microscopy images of naïve (b) or IFNγ-primed HeLa (c) infected with *Salmonella*-dsRed, at 1 h and 5 h p.i.. Arrows point to nuclear condensation and arrowheads to plasma membrane swelling and blebbing. Scale bars correspond to 10 *μ*m. DIC, differential interference contrast.

d. Propidium iodide (PI) uptake in naïve or IFNγ-primed HeLa after infection with *Salmonella*.

e, f. Release of LDH (e) or IL-18 (f) in naïve or IFNγ-primed HeLa at the indicated time points after infection with *Salmonella*. N.I., non-infected.

g. Immunoblots for caspase-4, GSDMD and GAPDH (loading control) in cell lysates from wild-type, *CASP4*$^{-/-}$ or *GSDMD*$^{-/-}$ HeLa. FL points to full length caspase-4 (~45 kDa), and * inactive caspase-4 isoforms lacking the CARD domain resulting from CRISPR-Cas9 genome editing.

h-j. Assessment of *Salmonella* invasion in naïve or IFNγ-primed wild-type, *CASP4*$^{-/-}$ or *GSDMD*$^{-/-}$ HeLa cells, at 1 h p.i.. Cells were infected as described in (a) and then were lysed and the number of viable intracellular bacteria was counted by CFUs (h), In (i, j), cells were infected with *Salmonella*-dsRed, fixed and then the percentage of infected cells or the number of bacteria per infected cell were counted by fluorescence microscopy. At least 80 cells (i) or 30 infected cells (j) were counted, in triplicate conditions.

k. PI uptake in IFNγ-primed wild-type, *CASP4*$^{-/-}$ or *GSDMD*$^{-/-}$ HeLa after infection with *Salmonella*.

l. Intracellular bacterial fold-replication in naïve or IFNγ-primed wild-type, *CASP4*$^{-/-}$ or *GSDMD*$^{-/-}$ HeLa cells, at 1 or 6 h p.i. with *Salmonella*. Cells in 96-well plates were infected for 30 min, washed and gentamicin was added to kill extracellular bacteria. At the indicated time points cells were lysed and the number of viable intracellular bacteria was determined by counting colony forming units (CFUs). The bacterial fold-replication was calculated versus 1 h p.i.

m. Schematic representation of infection of cells with a *Salmonella* reporter strain for cytosolic replication. Bacteria expressing GFP under the control of the hexose phosphate transporter promoter (P*uhpT*) only turn fluorescent in response to exogenous glucose-6-phosphate found exclusively in the host cytosol.

n, o. Cytosolic replication of *Salmonella* expressing P*uhpT*-GFP in naïve or IFNγ-primed wild-type (n) or *CASP4*$^{-/-}$ (o) HeLa cells. Cells were infected and fluorescence was recorded every 30 minutes using a plate reader.

p. Schematic representation of the chloroquine (CHQ) resistance assay used to determine the percentage of cytosolic *Salmonella*. When gentamicin is added only the extracellular bacteria are

killed (left panel), whereas addition of gentamicin and CHQ kills extracellular and vacuolar bacteria (right panel).

q. Percentage of cytosolic *Salmonella* in naïve or IFNγ-primed *GSDMD*$^{-/-}$ HeLa cells at 7 h p.i., quantified by the CHQ resistance assay. Cells in triplicate wells were infected for 30 min and then treated with gentamicin to kill extracellular bacteria. In some wells CHQ was also added for 1 h before cells were lysed and intracellular *Salmonella* enumerated. The percentage of CHQ resistant bacteria was calculated as the ratio of (CHQ+gentamicin^{resistant} / gentamicin^{resistant}).

r, s. Fluorescence confocal microscopy of *GSDMD*$^{-/-}$ HeLa cells infected with *Salmonella*-dsRed for 7 h (r). Arrowhead points to an example of a cell containing cytosolic hyper-replicating bacteria and arrows point to cells with non-hyper-replicating *Salmonella*. Scale bar corresponds to 10 μm. The percentage of hyper-replicating bacteria was quantified (s) by counting infected cells containing large accumulation of bacteria as shown in the image.

Graphs show the mean ± SD, and data are representative of two (e, g, r, s) or three (b-d, f, k, l, n, o) independent experiments or pooled from two (i, j, q), three (h) or four (a) independent experiments performed in triplicate. $^{*}P < 0.05$; $^{**}P < 0.01$; $^{***}P < 0.001$; ns, not significant; two-tailed *t*-test.

Supplementary Figure 2

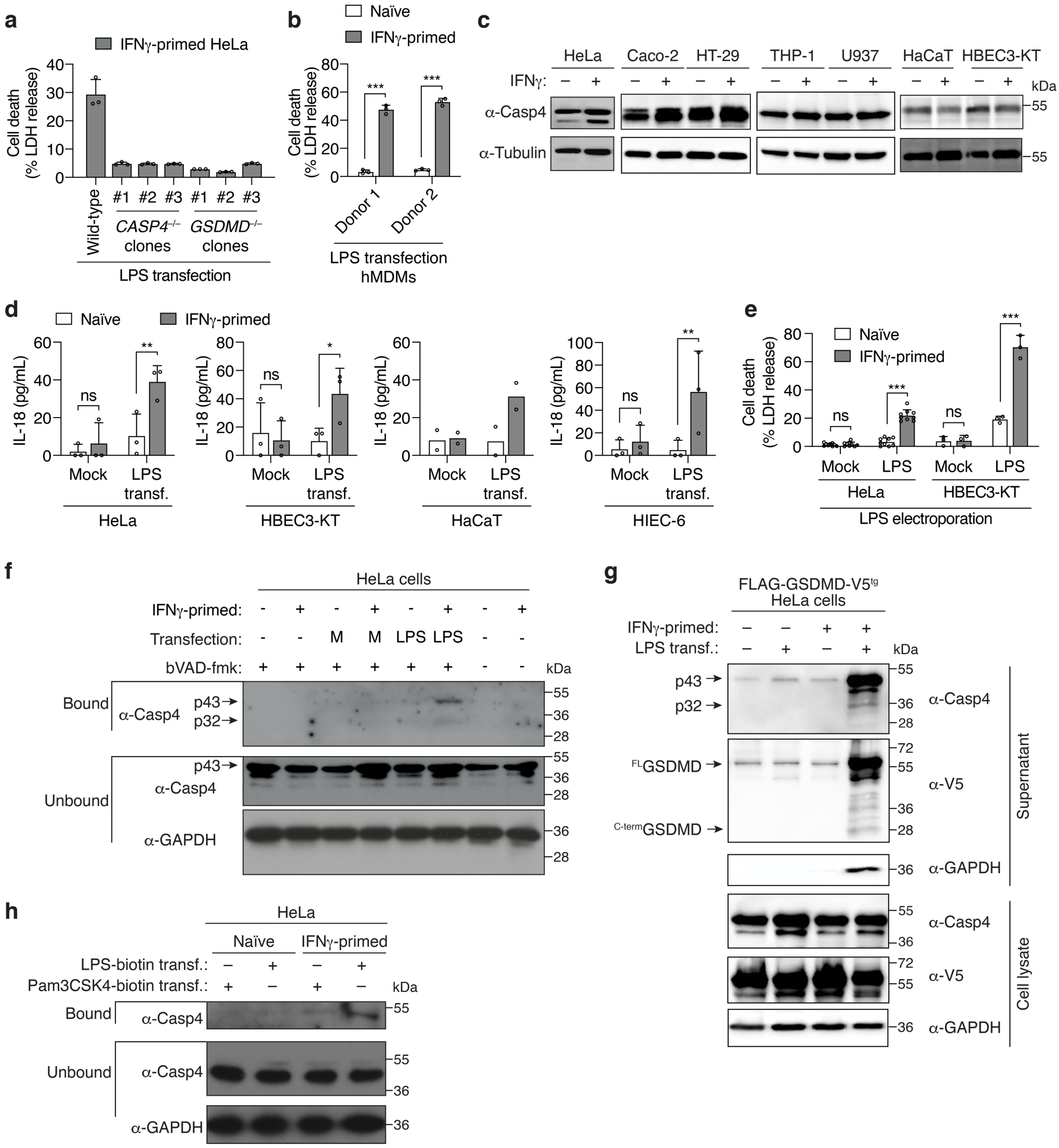

Supplementary Fig. 2. IFNγ priming induces LPS-induced caspase-4 activation.

a. Release of LDH from IFNγ-primed wild-type HeLa cells, or from different *CASP4*$^{-/-}$ or *GSDMD*$^{-/-}$ clones, 5 h after transfection with *E. coli*-derived LPS.

b. Release of LDH from naïve or IFNγ-primed primary human monocyte-derived macrophages (hMDMs), 5 h after transfection with *E. coli*-derived LPS.

c. Immunoblot analysis of caspase-4 expression in cell lysates from naïve or IFNγ-primed cells.

d. Release of IL-18 from naïve or IFNγ-primed cells, 5 h after transfection with *E. coli*-derived LPS.

e. Release of LDH from naïve or IFNγ-primed HeLa or HBEC3-KT cells, 3-4 h after electroporation with *E. coli* LPS.

f. Pull-down of active caspase-4 from HeLa, using bVAD-fmk caspase activity probe. Approximately 3 x 10^6 cells were left untreated or primed with IFNγ and then transfected with *E. coli* LPS (20 μg) for 3 h. Mock-transfected (M) cells were used as a control. Streptavidin-bound and -unbound fractions were analyzed by western blot using an antibody against caspase-4.

g. Western blot analysis of full length (FL) and cleaved (p32) caspase-4, FL and cleaved (C-term) GSDMD in the supernatants or cell lysates from naïve or IFNγ-primed HeLa stably expressing FLAG-GSDMD-V5, upon transfection with *E. coli* LPS for 6 h.

h. Streptavidin pull-down assay of the binding of biotin-conjugated LPS to endogenous caspase-4 from the lysates of naïve or IFNγ-primed HeLa. Cells in 6-well plates were transfected with LPS-biotin (10 μg) or Pam3CSK4-biotin (2 μg) and biotinylated substrate was pulled down using equal amounts of streptavidin magnetic beads, which were then eluted in equal volumes of SDS-PAGE reducing sample buffer. Streptavidin-bound and -unbound fractions were analyzed by western blot using an antibody against caspase-4.

Graphs show the mean ± SD, and data are representative of two (b) or three independent experiments (a, c, e (HBEC3-KT) f-h), or pooled from three independent experiments (e, HeLa) performed in triplicate. Graphs shown in panel (d) were obtained from two (HaCaT) or three independent experiments (HeLa; HBEC3-KT; HIEC-6) where triplicate wells from the same experiment were first pooled together. *$P < 0.05$; **$P < 0.01$; *** $P < 0.001$; ns, not significant; two-tailed *t*-test.

Supplementary Figure 3

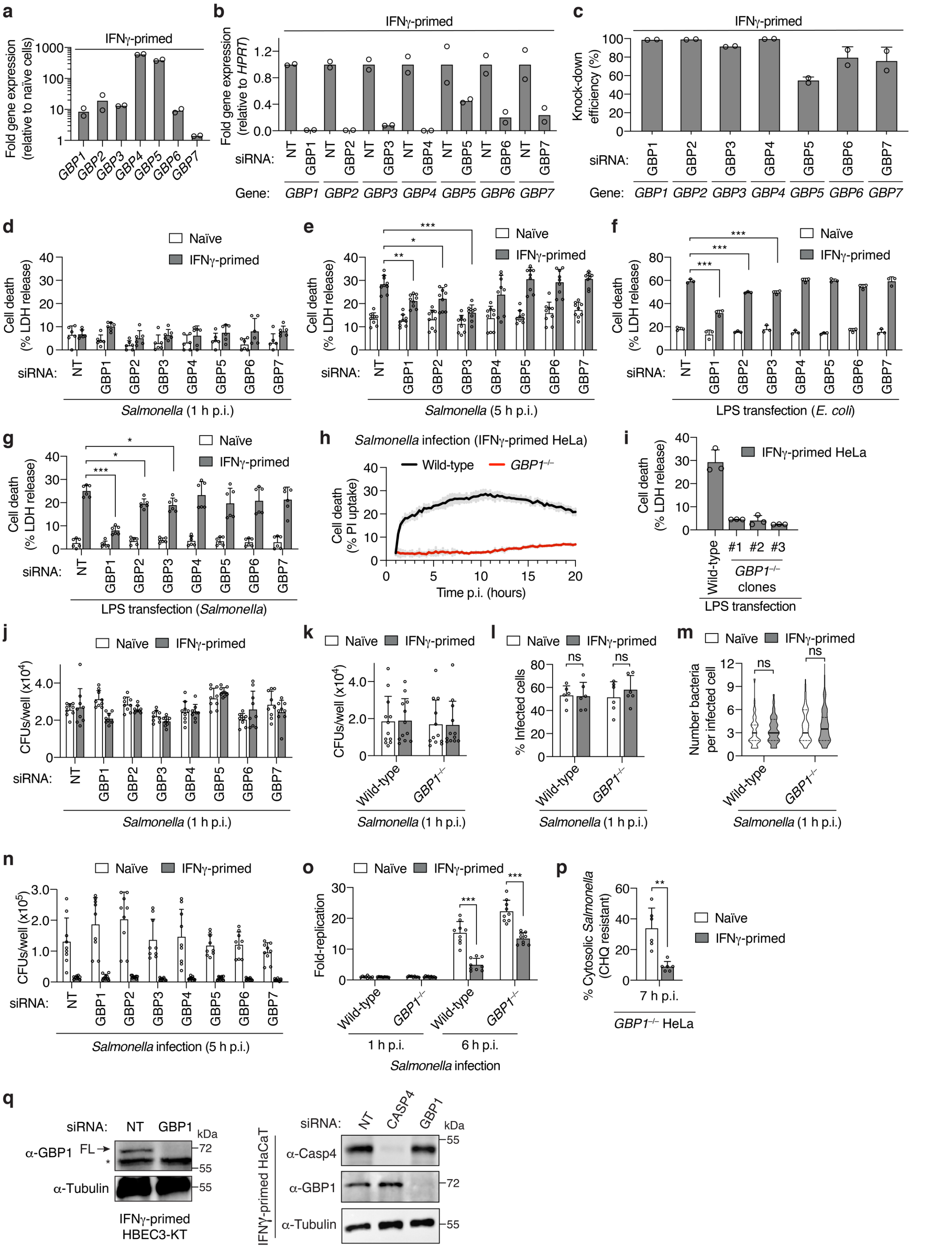

Supplementary Fig. 3. In epithelial cells, GBP1 controls non-canonical inflammasome activation in response to cytosolic LPS or *Salmonella*.

a. Expression levels of *GBP1-7* in IFNγ-primed HeLa, assessed by qPCR. Fold gene expression is shown relative to naïve control cells. *HPRT* was used as a housekeeping gene.

b. Expression levels of *GBP1-7* in IFNγ-primed HeLa upon treatment with non-targeting control siRNA (NT) or with siRNAs targeting *GBP1-7* for 24 h. Fold expression for each gene was assessed by qPCR, relative to control cells treated with NT siRNA. *HPRT* was used as a housekeeping gene.

c. Percentage of knock-down efficiency calculated as $(1 - 2^{\Delta\Delta Ct}) \times 100$.

d, e. Release of LDH from naïve or IFNγ-primed HeLa cells treated with siRNAs against GBPs, 1 h (d) or 5 h (e) after *Salmonella* infection. Cells were treated with non-targeting control siRNA (NT) or with siRNAs targeting *GBP1-7* for 24 h and infected for 30 min. Gentamicin was added to kill extracellular bacteria and cells were analyzed at the corresponding time points.

f, g. Release of LDH in naïve or IFNγ-primed HeLa treated with siRNAs against GBPs, 5 h after transfection with *E. coli*- (f) or *Salmonella*-derived LPS (g). Cells were treated with non-targeting control siRNA (NT) or with siRNAs targeting *GBP1-7* for 24 h and then transfected with LPS (2.5 μg/50,000 cells).

h. PI uptake in IFNγ-primed wild-type, or *GBP1*$^{-/-}$ HeLa after infection with *Salmonella*.

i. Release of LDH from IFNγ-primed wild-type HeLa, or from different *GBP1*$^{-/-}$ clones, 5 h after transfection with *E. coli*-derived LPS (2.5 μg/50,000 cells).

j-m. Assessment of *Salmonella* invasion at 1 h p.i., in naïve or IFNγ-primed HeLa treated with siRNAs against GBPs (i) or in wild-type and *GBP1*$^{-/-}$ cells (k-m). In (j), cells were treated with non-targeting control siRNA (NT) or with siRNAs targeting *GBP1-7* for 24 h and infected for 30 min. Cells were then washed and gentamicin was added to kill extracellular bacteria. The cells were lysed and the number of viable intracellular bacteria was counted by CFUs (j,k). In (l, m) cells were infected with *Salmonella*-dsRed, fixed and then the percentage of infected cells or the number of bacteria per infected cell were counted by fluorescence microscopy.

n, o. Intracellular bacterial replication in naïve or IFNγ-primed HeLa treated with siRNAs against GBPs (n) or in wild-type and *GBP1*$^{-/-}$ cells (o) after infection with *Salmonella*, as determined by counting CFUs. The bacterial fold-replication was calculated relative to 1 h p.i..

p. Percentage of cytosolic *Salmonella* in naïve or IFNγ-primed *GBP1*$^{-/-}$ HeLa at 7 h p.i., quantified by the CHQ resistance assay. Cells in triplicate wells were infected for 30 min and then treated with gentamicin to kill extracellular bacteria. In some wells CHQ was also added for 1 h before cells were lysed and intracellular *Salmonella* enumerated.

q. Immunoblot for GBP1 and caspase-4 expression following treatment with non-targeting siRNA (NT) or siRNA against GBP1 or CASP4 in IFNγ-primed HBEC3-KT or HaCaT cells, as used in Fig. 2g. (*denotes a cross-reactive band).

Graphs show the mean ± SD, and data are pooled from at least two independent experiments performed in triplicate (d-e, g, j-p), or representative of at least two independent experiments performed in duplicate (a-c) or in triplicate (f, h, i, q). *$P < 0.05$; **$P < 0.01$; *** $P < 0.001$; ns, not significant; two-way ANOVA (e-g) or two-tailed *t*-test (l, m, o, p).

Supplementary Figure 4

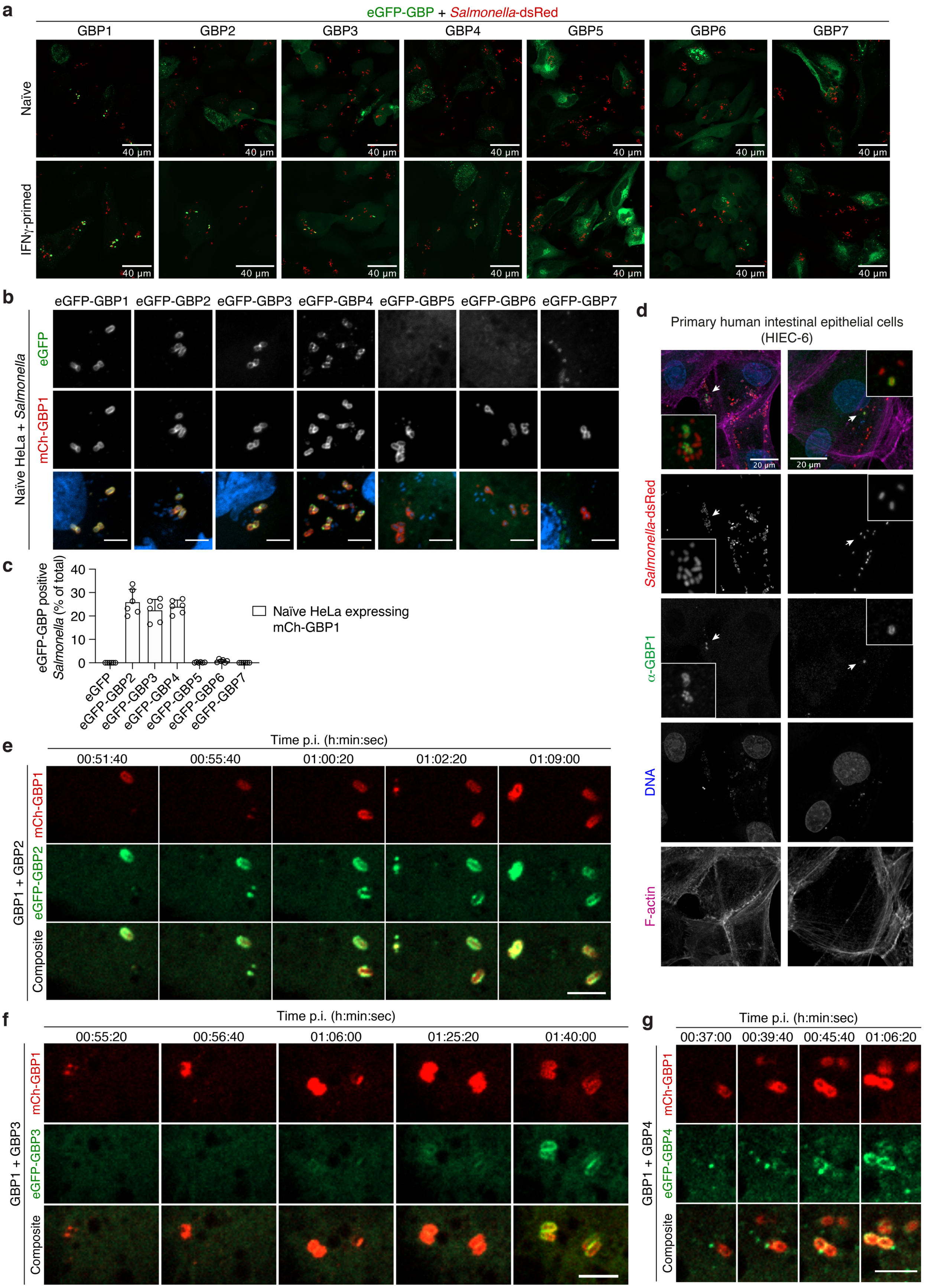

Supplementary Fig. 4. GBP1 regulates hierarchical recruitment of GBP2-4 to intracellular *Salmonella*.

a. Fluorescence confocal microscopy of naïve or IFNγ-primed HeLa expressing N-terminal tagged eGFP-GBP1-7 (green) and infected with *Salmonella*-dsRed (red) for 1h. Representative confocal images are shown and scale bars correspond to 40 μm.

b. Fluorescence confocal microscopy of naïve HeLa co-expressing mCherry-GBP1 (red) and eGFP-GBP1-7 (green) and infected with *Salmonella* for 1 h. DNA was stained with Hoechst (blue). Representative confocal images are shown and scale bars correspond to 5 μm.

c. Percentage of intracellular *Salmonella* positive for eGFP-GBP2-7 in naïve HeLa co-expressing mCherry-GBP1, at 1 h p.i.. At least 100 bacteria were counted per coverslip.

d. Fluorescence confocal microscopy of IFNγ-primed HIEC-6 cells infected with *Salmonella*-dsRed for 1.5 h. GBP1 (green) was visualized by immunostaining with a rabbit anti-GBP1 antibody (ab121039, abcam), DNA and F-actin were stained with Hoechst (blue) or Phalloidin-647 (purple), respectively. Representative confocal images are shown. Arrows point to enlarged images.

e-g. Time-lapse fluorescence confocal microscopy of HeLa co-expressing mCherry-GBP1 (red) and eGFP-GBP2 (e), eGFP-GBP3 (f) or eGFP-GBP4 (g) and infected with *Salmonella.* Scale bars correspond to 5 μm.

Graph shown the mean ± SD, and data are pooled from three independent experiments performed in duplicate (c) or representative of two (a, g) or three (e, f) independent experiments.

Supplementary Figure 5

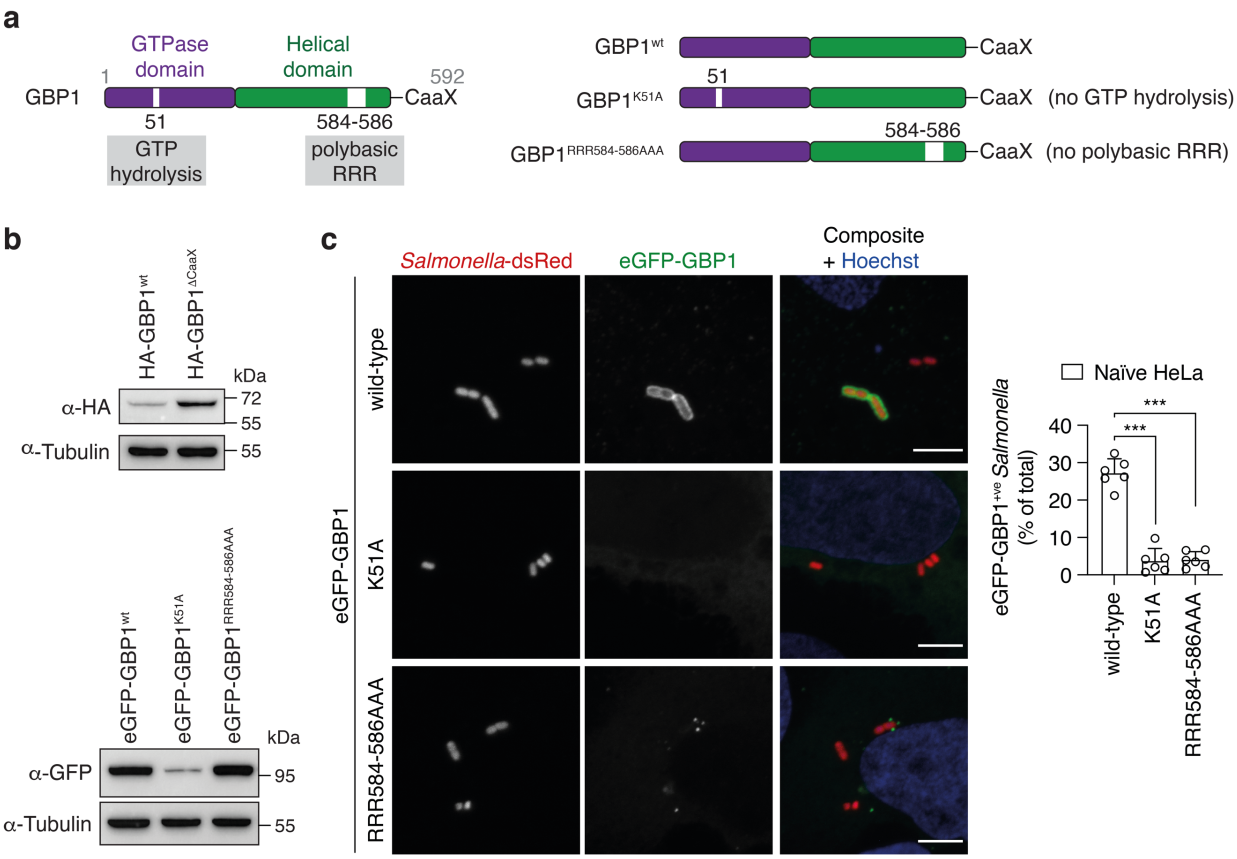

Supplementary Fig. 5. GBP1 recruitment to intracellular *Salmonella* is dependent on its GTP hydrolysis and polybasic C-terminal motives.

a. Schematic representation of wild-type GBP1 and two different mutants.

b. Western blot analysis for expression of HA-GBP1^{wt} and HA-GBP1$^{\Delta CaaX}$, or eGFP-GBP1^{wt}, eGFP-GBP1^{K51A} and eGFP-GBP1$^{RRR584\text{-}586AAA}$ in naïve HeLa cells.

c. Fluorescence confocal microscopy of naïve HeLa expressing eGFP-GBP1^{wt}, eGFP-GBP1^{K51A} or eGFP-GBP1$^{RRR584\text{-}586AAA}$ (green) and infected with *Salmonella*-dsRed (red) for 1 h. DNA was stained with Hoechst (blue). Representative confocal images are shown and scale bars correspond to 5 μm. The percentage of eGFP-GBP1 positive *Salmonella* was quantified by counting around 100 bacteria per coverslip and the graph shows the mean ± SD from three independent experiment performed in triplicate. *** $P < 0.001$; two-tailed *t*-test.

Data are representative from three independent experiments (b, c).

Supplementary Figure 6

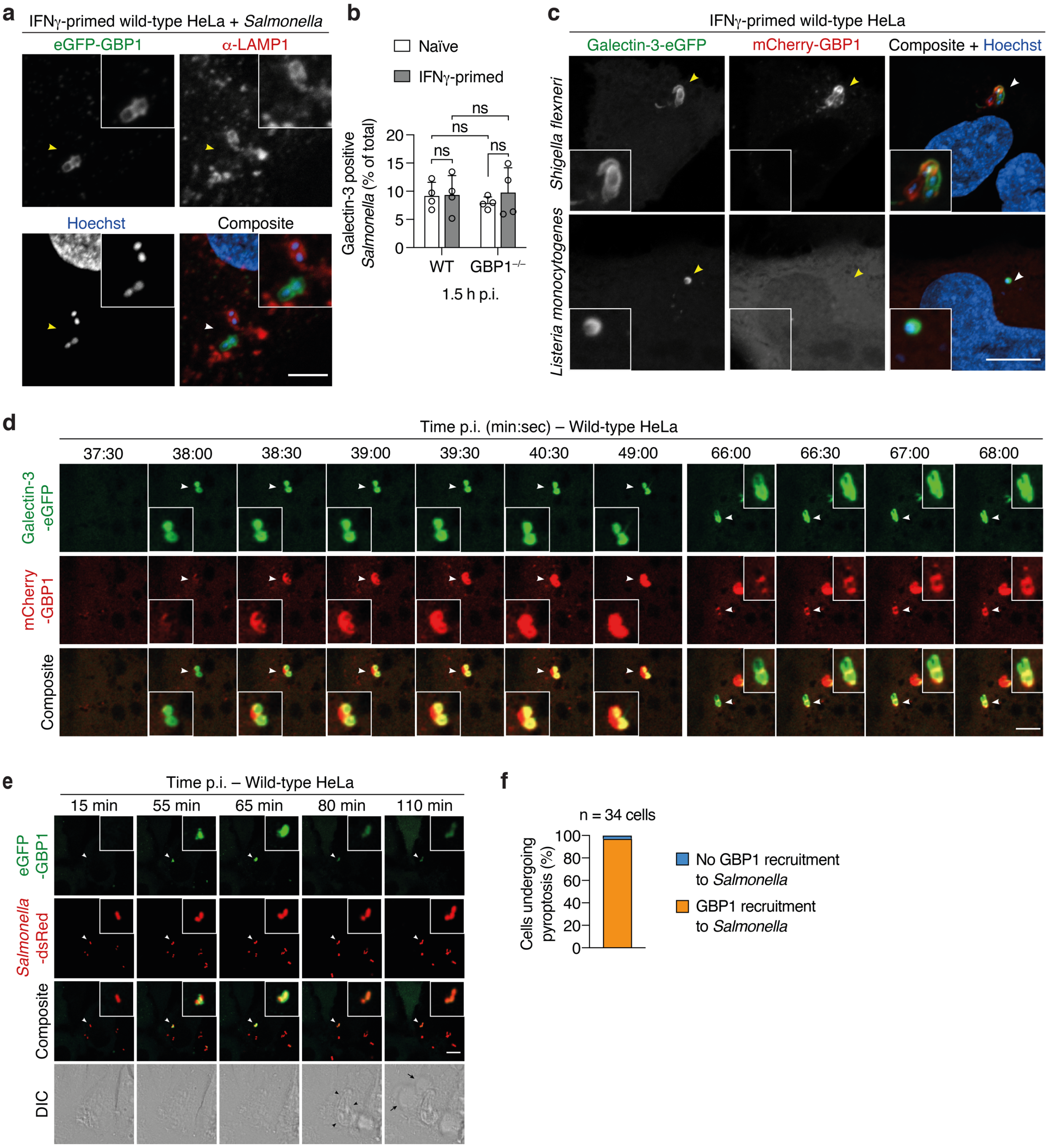

Supplementary Fig. 6. Human GBP1 does not induce SCV rupture and is recruited to cytosolic *Salmonella* immediately after SCV membrane rupture.

a. Fluorescence confocal microscopy of IFNγ-primed HeLa cells expressing eGFP-GBP1 (green) and infected with *Salmonella* for 1 hour. LAMP1 (red) was visualized by immunostaining with a rabbit anti-Lamp1 antibody (abcam 24170) and DNA was stained with Hoechst (blue). Representative confocal images are shown. Arrowheads point to enlarged images and scale bar corresponds to 5 μm.

b. Percentage of galectin-3 positive *Salmonella* in naïve or IFNγ-primed wild-type or *GBP1*$^{-/-}$ HeLa at 1.5 h p.i.. Cells expressing galectin-3-eGFP were infected with *Salmonella*-dsRed, fixed and analyzed by fluorescence confocal microscopy.

c. Fluorescence confocal microscopy of IFNγ-primed HeLa cells co-expressing galectin-3-eGFP (green) and mCherry-GBP1 (red) and infected with *S. flexneri* or *L. monocytogenes* for 30 min. DNA was stained with Hoechst (blue) and representative confocal images are shown. Arrowheads point to enlarged images and scale bar corresponds to 10 μm.

d. Time-lapse fluorescence confocal microscopy of IFNγ-primed wild-type HeLa co-expressing galectin-3-eGFP (green) and mCherry-GBP1 (red) and infected with *Salmonella*. Images were acquired every 30 seconds and scale bar corresponds to 5 μm.

e. Time-lapse fluorescence confocal microscopy of IFNγ-primed wild-type HeLa expressing eGFP-GBP1 (green) and infected with *Salmonella*-dsRed (red). Black arrowheads point to nuclear condensation and black arrows point to plasma membrane blebbing of a pyroptotic cell. Scale bar corresponds to 10 μm. DIC, differential interference contrast.

f. Percentage of cells undergoing pyroptosis in which there was eGFP-GBP1 recruitment to *Salmonella* (orange, 33 out of 34 cells) or no detectable eGFP-GBP1 recruitment (blue, 1 out of 34 cells), as determined by time-lapse fluorescence microscopy.

Graph shows the mean ± SD, and data are pooled from two (b) independent experiments performed in duplicate or representative of two (a, c) or at least three independent experiments (d-f). ns, not significant, two-tailed *t*-test.

Supplementary Figure 7

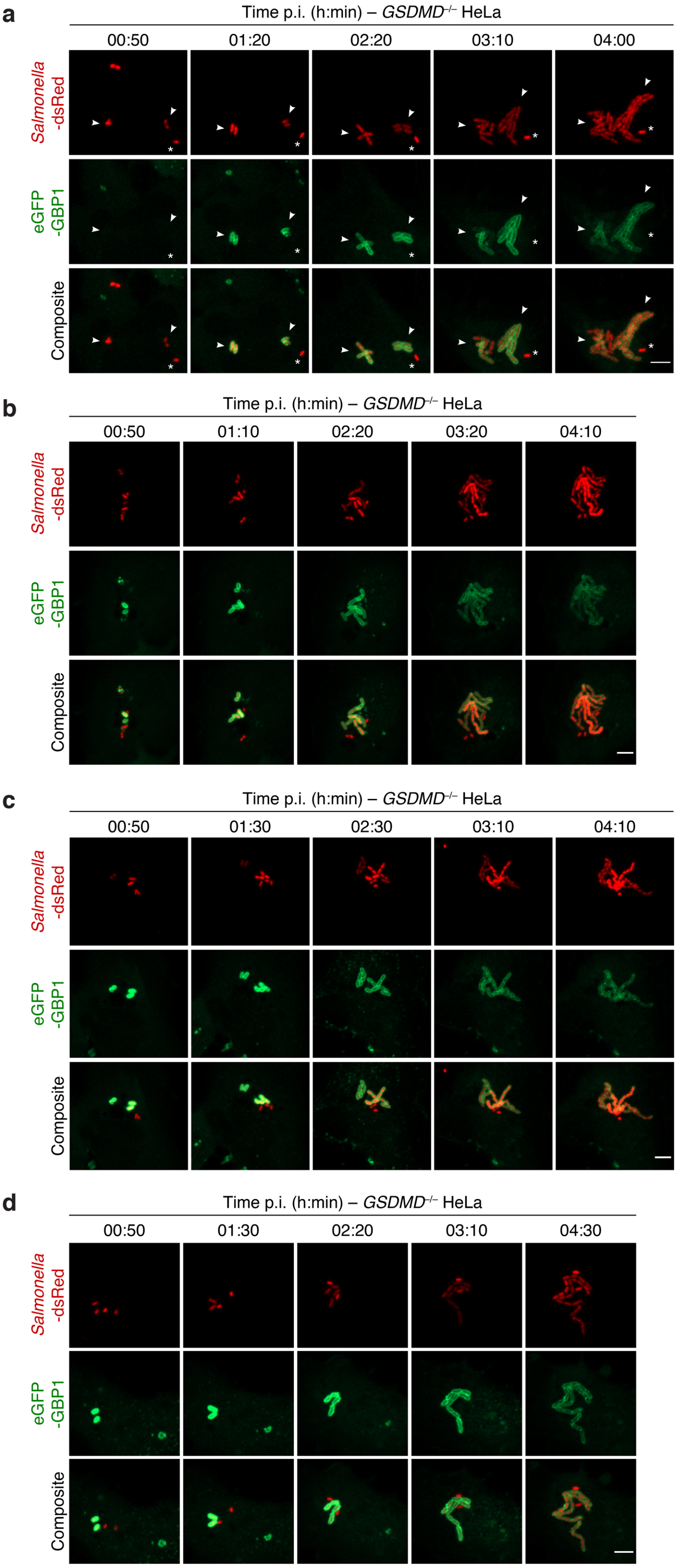

Supplementary Fig. 7. Recruitment of GBP1 to intracellular *Salmonella* does not promote bacteriolysis or restrict replication in *GSDMD*-deficient cells

a-d. Time-lapse fluorescence confocal microscopy of IFNγ-primed *GSDMD*$^{-/-}$ HeLa expressing eGFP-GBP1 (green) and infected with *Salmonella*-dsRed (red). Cytosolic bacteria are targeted by GBP1 and undergo hyper-replication. In (a), arrowheads point to cytosolic bacteria that are targeted by GBP1 and undergo hyper-replication, whereas asterisk points to a GBP1-negative bacterium that does not replicate, and most likely remains within the SCV. Scale bars corresponds to 5 μm.

Data are representative three independent experiments.

Supplementary Figure 8

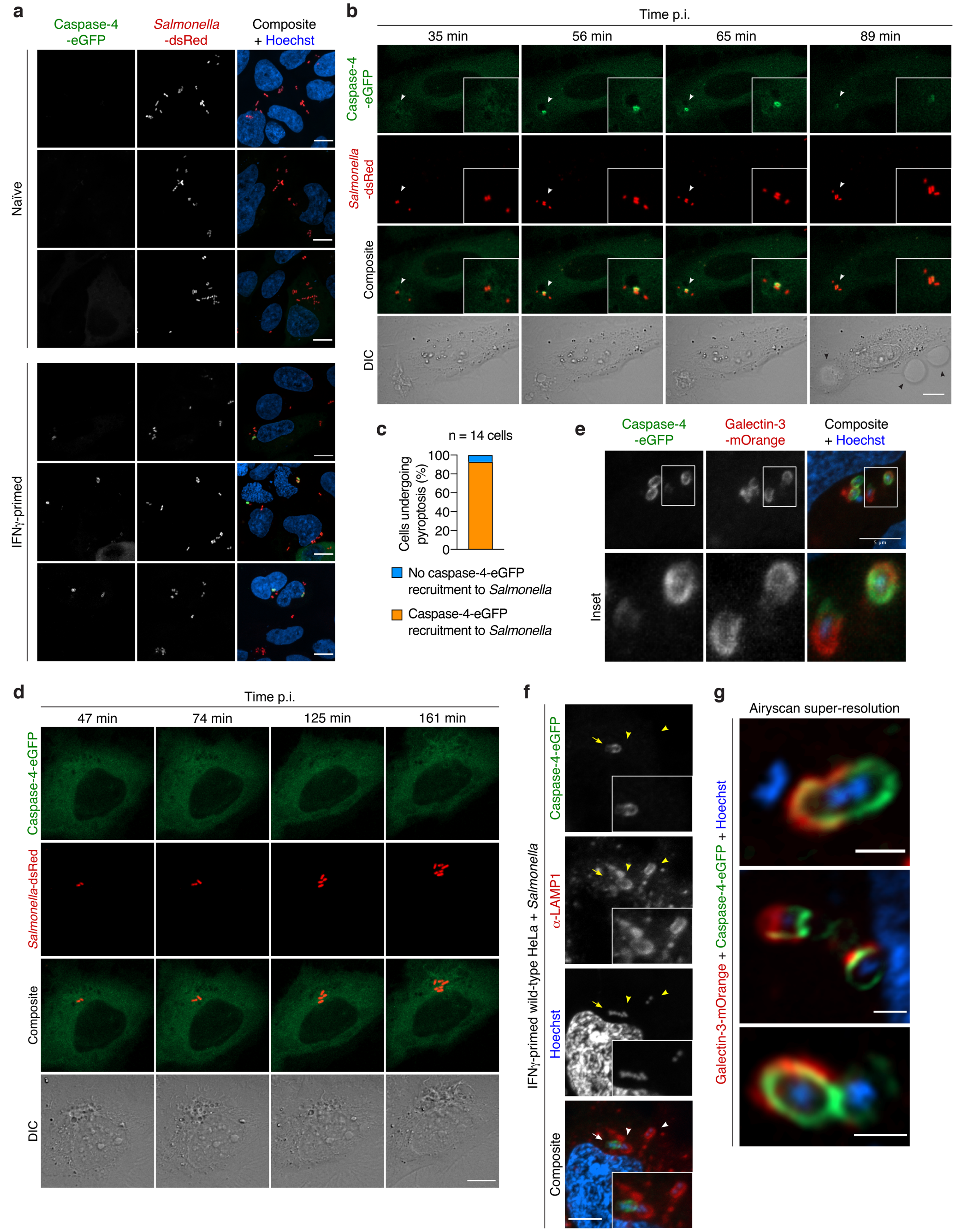

Supplementary Fig. 8. Caspase-4 is recruited to cytosolic *Salmonella* after escape from the SCV to induce pyroptosis in epithelial cells.

a. Fluorescence confocal microscopy of naïve or IFNγ-primed HeLa expressing caspase-4-eGFP (green) and infected with *Salmonella*-dsRed for 1 h. DNA was stained with Hoechst (blue) and representative confocal images are shown. Scale bars correspond to 10 μm.

b, d. Time-lapse fluorescence confocal microscopy of IFNγ-primed HeLa expressing caspase-4-eGFP (green) and infected with *Salmonella*-dsRed. Black arrowheads, in (b), point to plasma membrane blebbing of a pyroptotic cell. Scale bars correspond to 10 μm. DIC, differential interference contrast.

c. Percentage of cells undergoing pyroptosis in which there was caspase-4-eGFP recruitment to *Salmonella* (orange, 13 out of 14 cells) or no caspase-4-eGFP recruitment (blue, 1 out of 14 cells).

e, g. Fluorescence confocal microscopy of IFNγ-primed HeLa cells co-expressing caspase-4-eGFP (green) and galectin-3-mOrange (red) and infected with *Salmonella* for 1 h. DNA was stained with Hoechst (blue) and representative confocal images are shown. Scale bars correspond to 5 μm (E) or 1 μm (G).

f. Fluorescence confocal microscopy of IFNγ-primed HeLa cells expressing caspase-4-eGFP (green) and infected with *Salmonella* for 1 hour. LAMP1 (red) was visualized by immunostaining with a rabbit anti-Lamp1 antibody (abcam 24170) and DNA was stained with Hoechst (blue). Representative confocal images are shown. Arrowheads point to enlarged images and scale bar corresponds to 5 μm.

Data are representative of three independent experiments.

Supplementary Figure 9

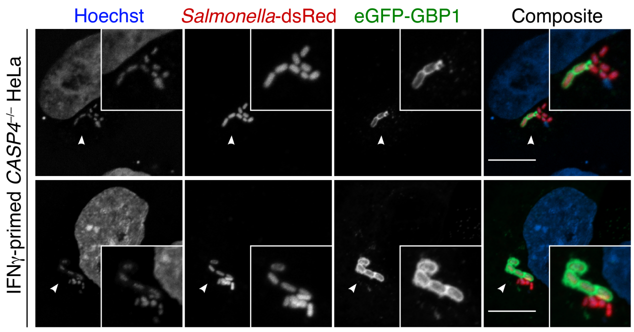

Supplementary Fig. 9. GBP1 recruitment to *Salmonella* is independent of caspase-4. Fluorescence confocal microscopy in IFNγ-primed *CASP4*$^{-/-}$ HeLa expressing eGFP-GBP1 (green) and infected with *Salmonella*-dsRed (red) for 1 h. DNA was stained with Hoechst (blue) and two sets of representative confocal images are shown. Scale bar correspond to 10 μm. Images are representative of two independent experiments.

Supplementary Figure 10

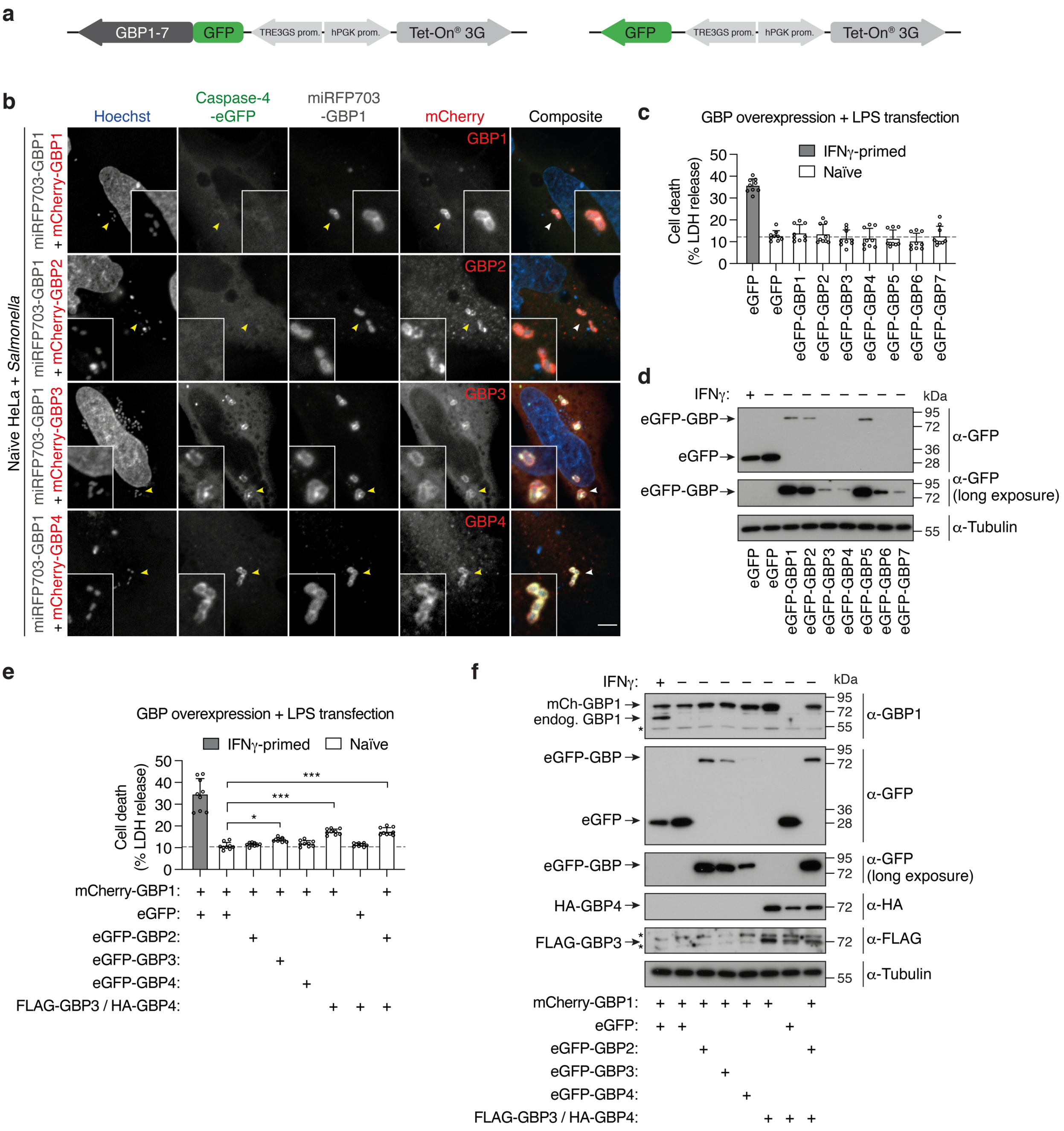

Supplementary Fig. 10. GBP1/3/4 overexpression is sufficient to recruit caspase-4 on *Salmonella* and induce pyroptosis in human epithelial cells.

a. Schematic representation of the pLVX-Tet-On 3G plasmids used to express doxycycline (Dox)-inducible GFP or GFP-GBP1-7.

b. Fluorescence confocal microscopy of naïve HeLa cells co-expressing caspase-4-eGFP (green), miRFP703-GBP1 (grey) and mCherry-GBP1, -2, -3 or -4 (red), and infected with *Salmonella* for 1 h. DNA was stained with Hoechst (blue). Representative confocal images are shown and scale bar corresponds to 5 μm.

c. LDH release from naïve HeLa cells expressing Dox-inducible eGFP-GBP1-7 or eGFP. Cells were transfected with the indicated plasmids for 24 h. eGFP-GBPs were induced for 16 h with 1 μg/ml Dox, whereas eGFP was induced for 3 h. IFNγ-primed cells expressing eGFP were used as a positive control. Cells were then transfected with *E. coli*-derived LPS (2.5 μg / 50,000 cells) for 6 h.

d. Western blot analysis of Dox-inducible eGFP-GBP1-7 or eGFP expression in HeLa cells.

e. LDH release from naïve HeLa cells co-expressing constitutive mCherry-GBP1 and Dox-inducible eGFP or eGFP-GBP1, -2, -3 or -4. FLAG-GBP3 and HA-GBP4 were constitutively expressed together using a bicistronic plasmid. Cells were transfected with the indicated plasmids for 24 h. eGFP-GBPs were induced for 16 h with 1 μg/ml Dox, whereas eGFP was induced for 3 h. Cells were then transfected with *E. coli*-derived LPS (2.5 μg / 50,000 cells) for 6 h.

f. Western blot analysis of mCherry-GBP1 and endogenous (endog.) GBP1 expression (α-GBP1), Dox-inducible eGFP-GBP2-4 or eGFP expression (α-GFP), HA-GBP4 (α-HA) or FLAG-GBP3 (α-FLAG) expression, in HeLa cells. Asterisks point to non-specific bands.

Graphs show the mean ± SD, and data are pooled from three independent experiments performed in triplicate (c, e) or are representative of two (b) or three (d, f) independent experiments. *$P < 0.05$; *** $P < 0.001$; one-way ANOVA.

Supplementary Figure 11

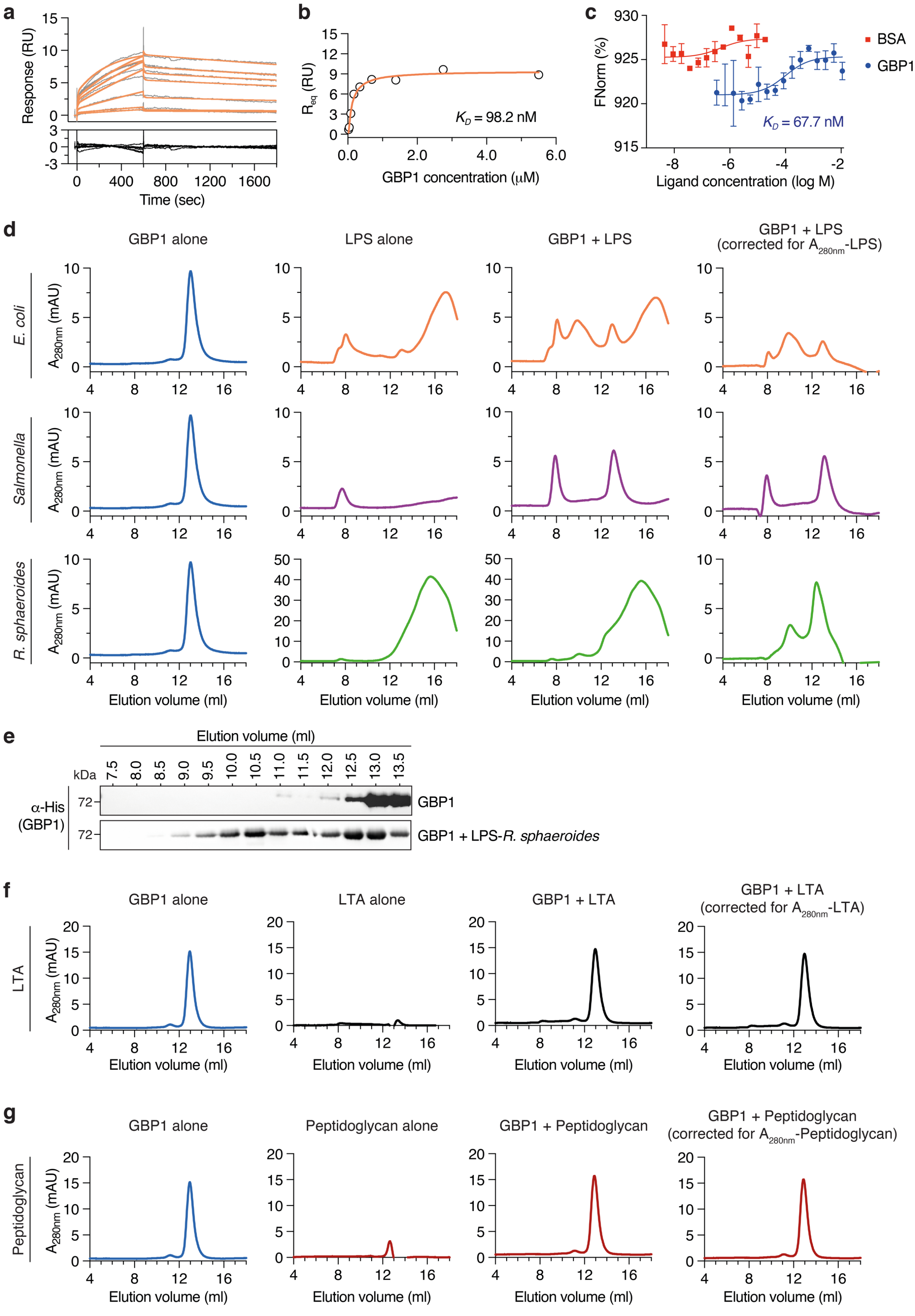

Supplementary Fig. 11. GBP1 interacts with LPS

a. SPR sensorgram of human GBP1 binding to *E. coli*-LPS (O111:B4) immobilized on a CM5 chip surface. Sensorgram was obtained by using different GBP1 concentrations (21.5, 43, 86, 172, 344, 688, 1375, 2750 nM). Grey lines correspond to SPR data and orange lines to model fits using a two-state-reaction model.

b. Saturation curve of the titration of GBP1 on LPS immobilized on a CM5 chip.

c. MST curves representing the interaction of GBP1 with FITC-LPS. BSA is used as a control.

d. SEC elution profiles of GBP1 alone, LPS from *E. coli*, *Salmonella* Typhimurium or *R. sphaeroides* or GBP1 incubated with the indicated LPS. Right panels show the SEC elution profiles after correction by subtracting the LPS-specific absorbance at 280 nm. Elution profiles are representative of 5-8 independent experiments.

e. Western blot analysis of His_6 after running individual fractions on a 12% acrylamide gel to confirm the presence of GBP1 in elution peaks.

f, g. SEC elution profiles of GBP1 alone, lipoteichoic acid (LTA) or GBP1 incubated with LTA (f), or GBP1 alone, peptidoglycan or GBP1 incubated with peptidoglycan (g). Right panels show the SEC elution profiles after correction by subtracting the LTA- or peptidoglycan-specific absorbance at 280 nm.

Graphs show the mean ± SD, and data are representative from three (a-c) or five (d-g) independent experiments performed with at least three independently expressed and purified batches of recombinant His-GBP1.

Supplementary Figure 12

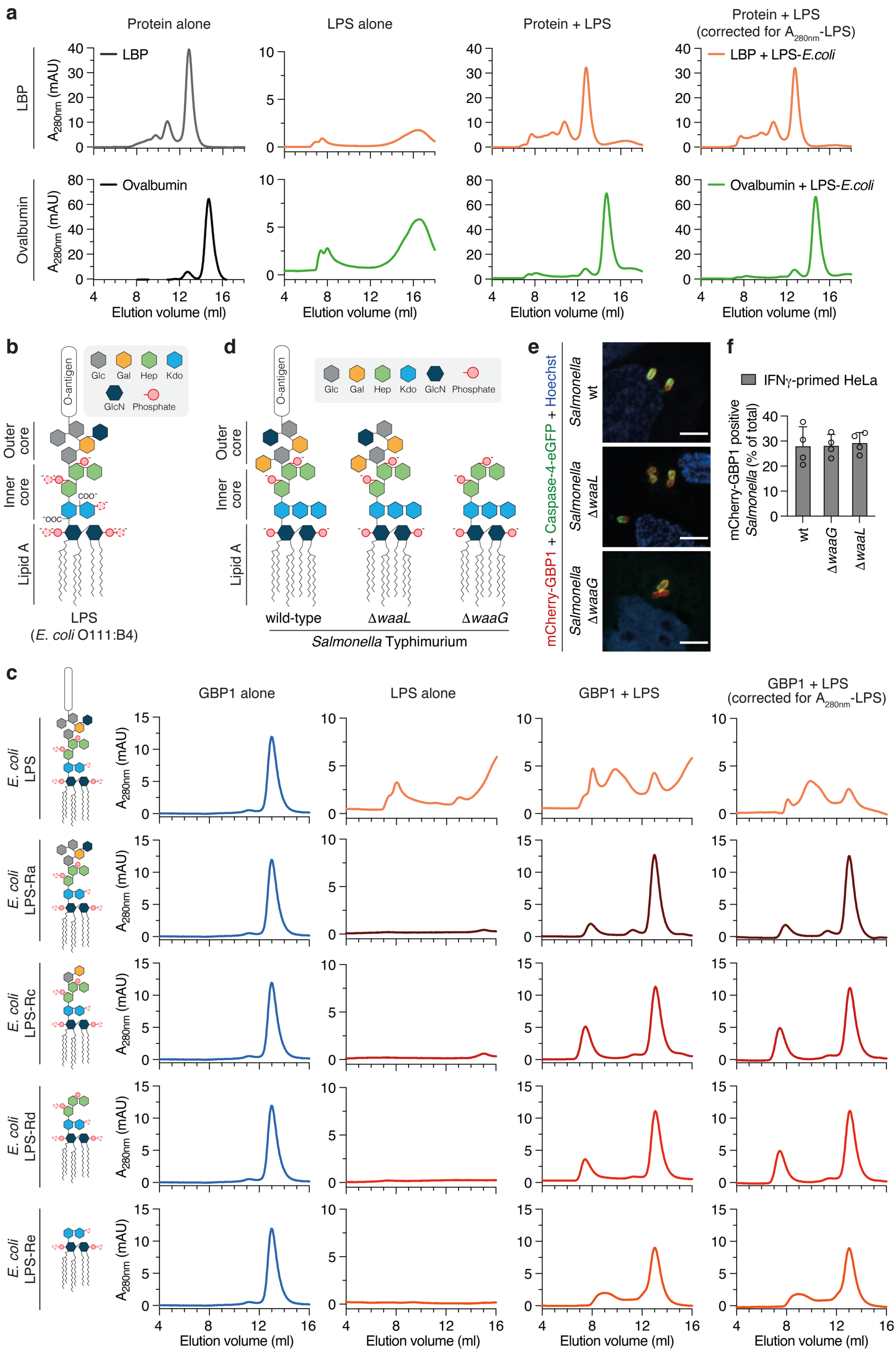

Supplementary Fig. 12. GBP1 interacts with the inner core and Lipid A region of LPS.

a. SEC elution profiles of LBP or ovalbumin alone, LPS from *E. coli*, or LBP or ovalbumin incubated with LPS. Right panels show the SEC elution profiles after correction by subtracting the LPS-specific absorbance at 280 nm.

b. Schematic representation of the LPS structure from *E. coli* serotype O111:B4, containing 6-8 negatively charged groups. Partial modifications that may result in additional phosphate groups are shown (dotted circles) and are dependent on growth conditions and other factors.

c. SEC elution profiles of GBP1, different *E. coli* LPS variants, or GBP1 incubated with indicated LPS variants. Right panel show corrected profiles after subtracting absorbance at 280 nm for each correspondent LPS variant.

d. Schematic representation of the LPS structure from *Salmonella* Typhimurium and different mutants.

e. Fluorescence confocal microscopy of IFNγ-primed HeLa cells co-expressing mCherry-GBP1 (red) and caspase-4-eGFP (green) and infected with wild-type (wt) *Salmonella* or with its isogenic Δ*waaL* or Δ*waaG* strains for 1h. DNA was stained with Hoechst (blue). Representative confocal images are shown and scale bar corresponds to 5 μm.

f. Percentage of mCherry-GBP1 positive *Salmonella* at 1 h p.i., as quantified by counting at least 100 bacteria per coverslip. Graph shows the mean ± SD.

Data are representative of two (e), or at least four (a, c) independent experiments, or pooled from two independent experiments performed in duplicate (f).

Supplementary Figure 13

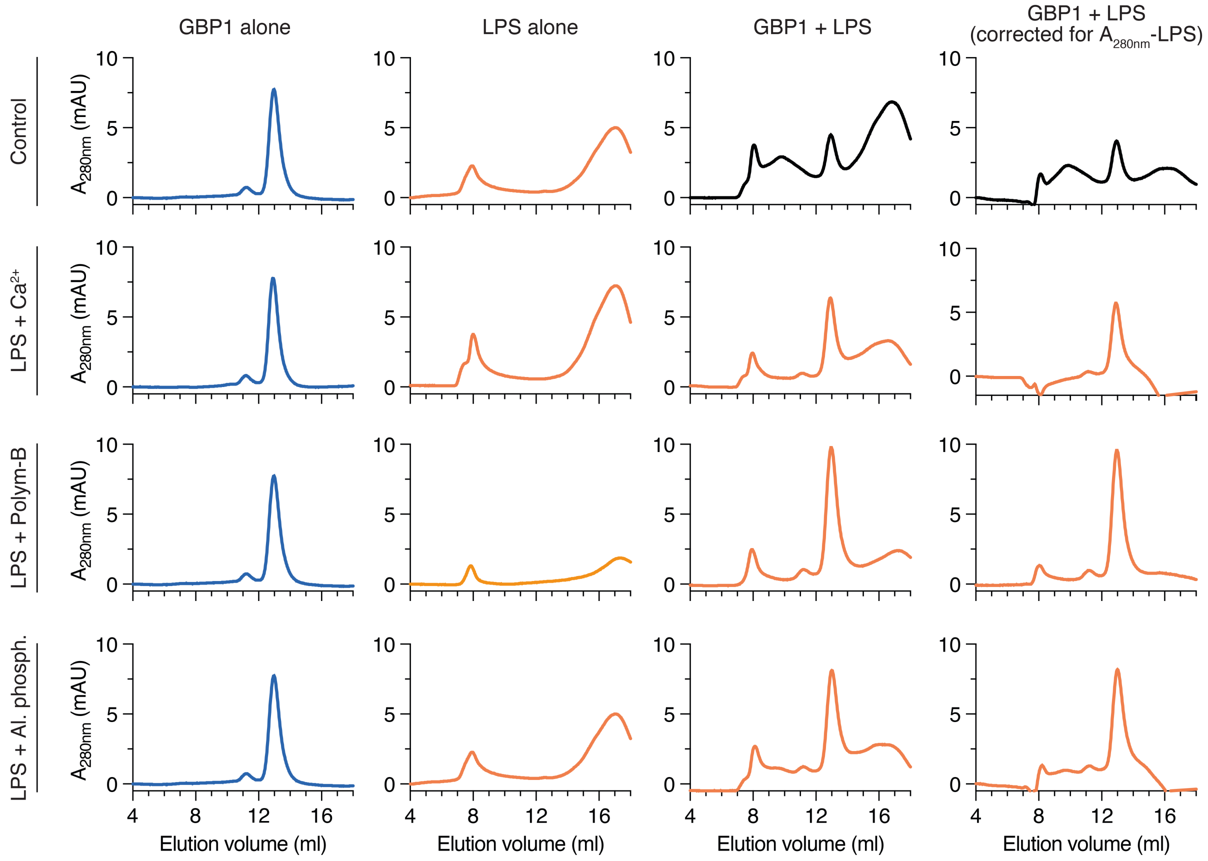

Supplementary Fig. 13. Negative charges on LPS are important for interaction with GBP1. SEC elution profiles of recombinant His-tagged GBP1 alone, *E. coli* LPS untreated or pre-treated with $CaCl_2$ (5 mM), Polymyxin B (10 µg/mL) or with alkaline phosphatase, or GBP1 incubated with the indicated LPS. Right panels show the SEC elution profiles after correction by subtracting the LPS-specific absorbance at 280 nm. Data are representative of at least three independent experiments.

Supplementary Figure 14

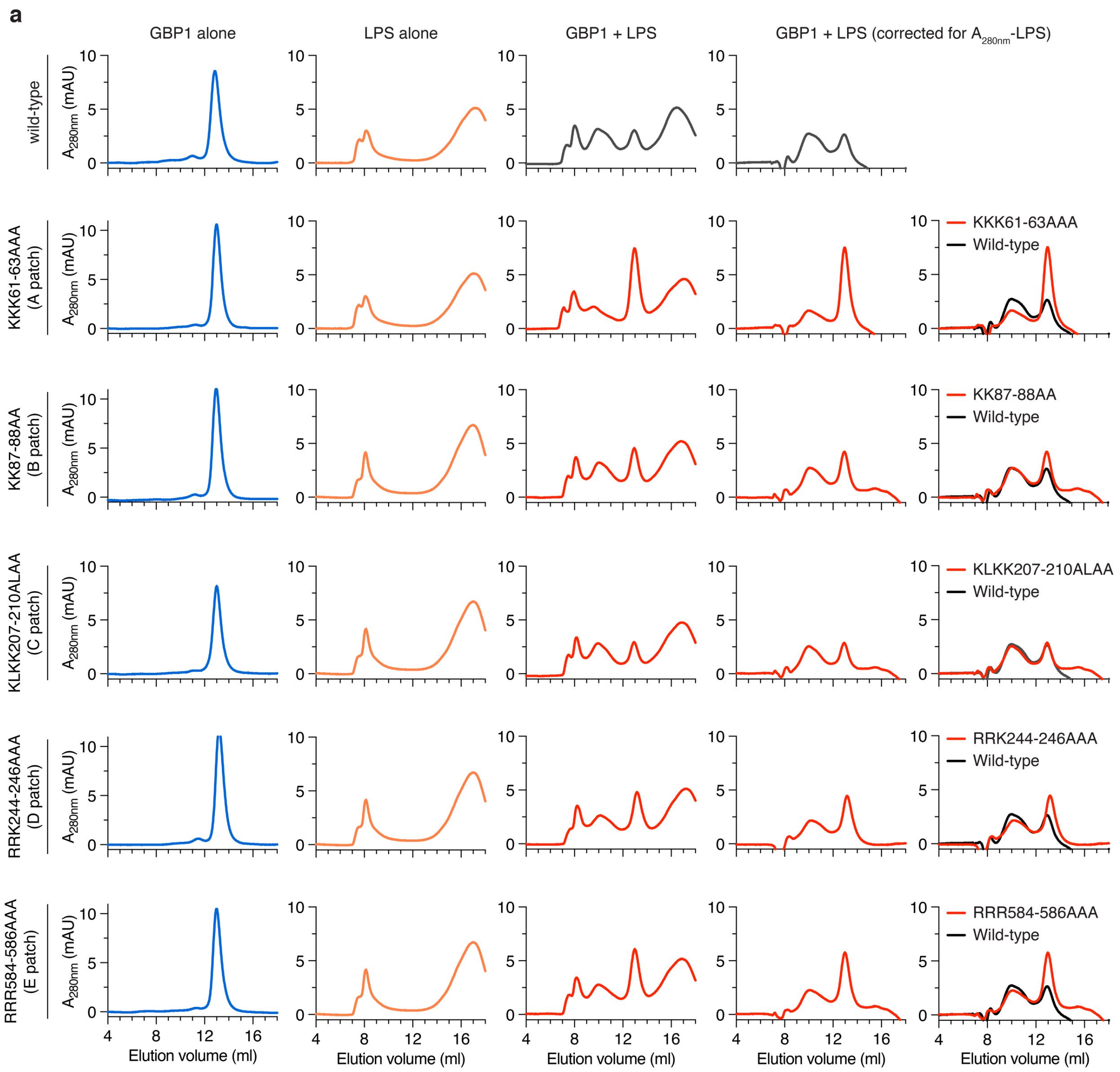

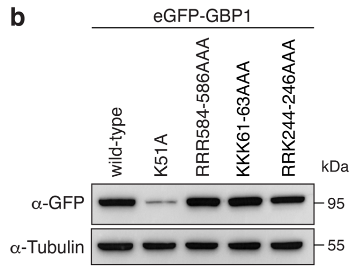

Supplementary Fig. 14. GBP1 binding to LPS involves electrostatic interactions.

a. SEC elution profiles of recombinant His-tagged GBP1 or of different GBP1 mutants, *E. coli* LPS, or the different GBP1 mutants after incubation with LPS. Right panels show the SEC elution profiles after correction by subtracting the LPS-specific absorbance at 280 nm. Data are representative of at least three independent experiments.

b. Western blot analysis for expression of different eGFP-tagged GBP1 mutants in naïve HeLa cells. Data are representative of two independent experiments.

Supplementary Figure 15

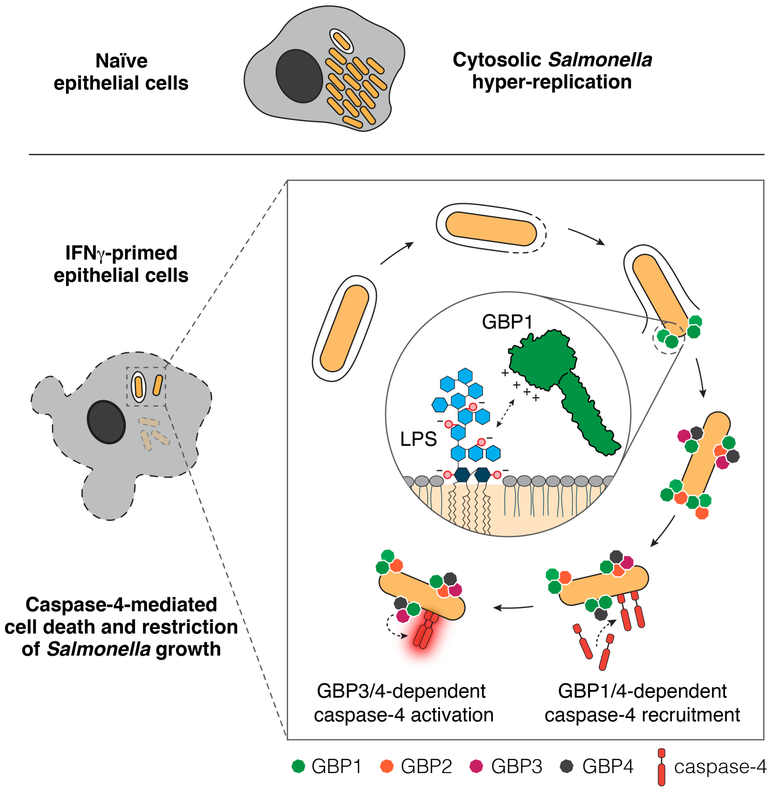

Supplementary Fig. 15. Model for GBP1 sensing of LPS and GBP-dependent recruitment of caspase-4 to cytosolic *Salmonella* for activation of non-canonical inflammasome in human epithelial cells.

In naïve epithelial cells (upper panel) *Salmonella* escape from the SCV to the cytosol, where they hyper-replicate. In IFNγ-primed epithelial cells (lower panel), GBP1 is recruited to the *Salmonella* surface immediately after SCV membrane rupture and bacterial escape to the host cytosol. GBP1 senses and binds to the LPS inner core and Lipid A, though electrostatic interactions, undergoing formation of a high-molecular weight complex and driving hierarchical recruitment of GBP2, -3 and -4 to the cytosolic bacteria. GBP1/4 additionally control recruitment of caspase-4 to the surface of cytosolic *Salmonella* and, together with GBP3, LPS-dependent activation of the caspase.

Supplementary Table 1 – Plasmids and primers used in this study.

Plasmid	Expressed protein	Primers used	Reference
pEGFP-GBP1	eGFP-GBP1	5'-GGACTCAGATCTCGAGCGATGGCATCAGAGATCCACA-3'<br>5'-GCAGAATTCGAAGCTTTTAGCTTATGGTACATGCCT-3'	This study
pEGFP-GBP1 K51A	eGBP-K51A	5'- CACAGGCGCATCCTACCTGATGAACAAGCTGGC-3'<br>5'- TAGGATGCGCCTGTGCGGTAGAGGCC-3'	This study
pEGFP-GBP2	eGFP-GBP2	5'-GGACTCAGATCTCGAGCGATGGCTCCAGAGATCAACT-3'<br>5'-GCAGAATTCGAAGCTTTTAGAGTATGTTACATATTGGC-3'	This study
pEGFP-GBP3	eGFP-GBP3	5'-GGACTCAGATCTCGAGCGATGGCTCCAGAGATCCAC-3'<br>5'-GCAGAATTCGAAGCTTTTAGATCTTTAGCTTATGCGA-3'	This study
pEGFP-GBP4	eGFP-GBP4	5'-GGACTCAGATCTCGAGCGATGGGTGAGAGAACTCTTC-3'<br>5'-GCAGAATTCGAAGCTTTAAATACGTGAGCCAAGATA-3'	This study
pEGFP-GBP5	eGFP-GBP5	5'-GGACTCAGATCTCGAGCGATGGCTTTAGAGATCCACAT-3'<br>5'-GCAGAATTCGAAGCTTTTAGAGTAAAACACATGGATC-3'	This study
pEGFP-GBP6	eGFP-GBP6	5'-GGACTCAGATCTCGAGCGATGGAATCTGGACCCAAAAT-3'<br>5'-GCAGAATTCGAAGCTTTTAAAAGGGGAGCTTATGCT-3'	This study
pEGFP-GBP7	eGFP-GBP7	5'-GGACTCAGATCTCGAGCGATGGCATCAGAGATCCACA-3'<br>5'-GCAGAATTCGAAGCTTTCAGCTTATAATTTTCTTACCA-3'	This study
pmCherry-GBP1	mCherry-GBP1	5'-GGACTCAGATCTCGAGCGATGGCATCAGAGATCCACA-3'<br>5'-GCAGAATTCGAAGCTTTTAGCTTATGGTACATGCCT-3'	This study
pmCherry-GBP2	mCherry-GBP2	5'-GGACTCAGATCTCGAGCGATGGCTCCAGAGATCAACT-3'<br>5'-GCAGAATTCGAAGCTTTTAGAGTATGTTACATATTGGC-3'	This study
pmCherry-GBP3	mCherry-GBP3	5'-GGACTCAGATCTCGAGCGATGGCTCCAGAGATCCAC-3'<br>5'-GCAGAATTCGAAGCTTTTAGATCTTTAGCTTATGCGA-3'	This study
pmCherry-GBP4	mCherry-GBP4	5'-GGACTCAGATCTCGAGCGATGGGTGAGAGAACTCTTC-3'<br>5'-GCAGAATTCGAAGCTTTAAATACGTGAGCCAAGATA-3'	This study
pmCherry-GBP5	mCherry-GBP5	5'-GGACTCAGATCTCGAGCGATGGCTTTAGAGATCCACAT-3'<br>5'-GCAGAATTCGAAGCTTTTAGAGTAAAACACATGGATC-3'	This study

pmCherry-GBP6	mCherry-GBP6	5’-GGACTCAGATCTCGAGCGATGGAATCTGGACCCAAAAT-3’<br>5’-GCAGAATTCGAAGCTTTTAAAAGGGGAGCTTATGCT-3’	This study
pmCherry-GBP7	mCherry-GBP7	5’-GGACTCAGATCTCGAGCGATGGCATCAGAGATCCACA-3’<br>5’-GCAGAATTCGAAGCTTTCAGCTTATAATTTTCTTACCA-3’	This study
piRFP703	iRFP703	5’-CGTCAGATCCGCTAGCGCCACCATGGTAGCAGGT-3’<br>5’-CTTGAGCTCGAGATCTTCGAGATCTGAGTCCGGAGCTCTCAAGCGCGGTGAT-3’	This study
piRFP703-GBP1	iRFP703-GBP1	5’-TCTCGAAGATCTCGAGCGATGGCATCAGAGATCCACA-3’<br>5’-GCAGAATTCGAAGCTTTTAGCTTATGGTACATGCCT-3’	This study
pAIP-HA-GBP1	HA-GBP1	————	Gift from T. Henry
pAIP-HA-GBP1$^{\Delta CAAX}$	HA-GBP1$^{\Delta CAAX}$	5’-TGCCTTTCGTCGTCTCATT-3’<br>5’-TAAAGACCAGAGCCTTCCTG-3’	This study
pAIP-HA-GBP2	HA-GBP2	————	Gift from T. Henry
pAIP-HA-GBP3	HA-GBP3	5’-TGCCTCTCCCGAATTCTGCAGATATCCATCACACTGGGACATGGCTCCAGAGATCCACATG-3’<br>5’-GAGAGGGGCGGAATTCACGGTCGATGTTAGATCTTTAGCTTATGCGACATATATCTCTTGG-3’	This study
pAIP-HA-GBP4	HA-GBP4	5’-TGCCTCTCCCGAATTCTGCAGATATCCATCACACTGGGACATGGGTGAGAGAACTCTTCACGC-3’<br>5’-GAGAGGGGCGGAATTCACGGTCGATGTTAAATACGTGAGCCAAGATATTTTGTCCCT-3’	This study
pLVX-eGFP-GBP1	eGFP-GBP1	5’-ACCGGTGCCGGCGGATCGCCACCATGGTGAGCAA-3’<br>5’-GAGGTGGTCTGGATCTTAGCTTATGGTACATGCCTTTCGT-3’	This study
pLVX-eGFP-GBP2	eGFP-GBP2	5’-ACCGGTGCCGGCGGATCGCCACCATGGTGAGCAA-3’<br>5’-GAGGTGGTCTGGATCTTAGAGTATGTTACATATTGGCTCCAATGA-3’	This study
pLVX-eGFP-GBP3	eGFP-GBP3	5’-ACCGGTGCCGGCGGATCGCCACCATGGTGAGCAA-3’<br>5’-GAGGTGGTCTGGATCTTAGATCTTTAGCTTATGCGACATATATCTCT-3’	This study
pLVX-eGFP-GBP4	eGFP-GBP4	5’-ACCGGTGCCGGCGGATCGCCACCATGGTGAGCAA-3’<br>5’-GAGGTGGTCTGGATCTTAAATACGTGAGCCAAGATATTTTGTCC-3’	This study
pEGFP-caspase-4	Caspase-4-eGFP	5’-TGGCAATGGTACCGAGCTCGGCT-3’<br>5’-GCAGAATTCGAAGCTTTTACTTGTACAGCTCGTCCATGCC-3’<br>5’-CGTCAGATCCGCTAGCCCCGCCACCATGGCAG-3’<br>5’-TCGGTACCATTGCCAGGAAAGAGGTAGAAATATCTTG-3’	This study
pCaspase-4-V5	Caspase-4-V5	5’- CGCGGGCCCGGGATCCACCATGGCAGAAGGCAACCAC-3’<br>5’- TCTAGAGTCGCGGCCTCACGT AGA ATC GAG ACC GAG GAG AGG GTT AGG GAT AGG CTT ACCGCCGCCATTGCCAGGAAAGAGGTAGAAATATC-3’	This study

pEGFP-Galectin3	Galectin3-eGFP	________	1
pmOrange-Galectin3	Galectin3-mOrange	________	2
pMyc-GBP1	Myc-GBP1	5'-AGCAGTACTTCTAGAGGATCGCCACCATGGAGCAGAAACTCATCTCTGAAGAGGATCTGGGATCCATGGCATCAGAGATCCACATGAC-3'<br>5'-TATCATGTCTGAATTCTTAGCTTAGCTTATGGTACATGCCTTTCGT-3'	This study
pFLAG-GBP3	FLAG-GBP3	5'-AGCAGTACTTCTAGAGGATCGCCACCATGGATTACAAGGATGACGACGATAAGGGATCCATGGCTCCAGAGATCCACATG-3'<br>5'-TATCATGTCTGAATTCTTAGCTTAGATCTTTAGCTTATGCGACATATATCTCTT-3'	This study
pFLAG-GBP3/HA-GBP4	FLAG-GBP3 + HA-GBP4	5'-TGAACACGTGGTCGCGGCCGGCCACCATGGGTTACCCTTATGATGTGCCAGATTATGCCAGCGGCCGCATGGGTGAGAGAACTCTTCACGC-3'<br>5'-CTGATCAGCGGGTTTAAACTTAAATACGTGAGCCAAGATATTTTGTCCCT-3'	This study
Pet28a-GBP1	His-GBP1	5'- CGCGCGGCAGCCATACACATCATATGGCATCAGAGATCCA-3'<br>5'- GGTGGTGGTGCTCGATTAGCTTATGGTACATGCCTTTCGT-3'	This study
Pet28a-GBP1m1	His-GBP1 K61-63A	5'- TGGAGCTGCTGCTGGCTTCTCTCTGGGCTCC-3'<br>5'- CCAGCAGCAGCTCCAGCCAGCTTGTTCATCAGG-3'	This study
eGFP-GBP1m1	GFP-GBP1 K61-63A	5'- TGGAGCTGCTGCTGGCTTCTCTCTGGGCTCC-3'<br>5'- CCAGCAGCAGCTCCAGCCAGCTTGTTCATCAGG-3'	This study
Pet28a-GBP1m2	His-GBP1 K87-88A	5'- ACCCCGCTGCTCCAGGCCACATCCTAGTTCTGC-3'<br>5'- CTGGAGCAGCGGGGTGGGGCACACACCACATCC-3'	This study
eGFP-GBP1m2	GFP-GBP1 K87-88A	5'- ACCCCGCTGCTCCAGGCCACATCCTAGTTCTGC-3'<br>5'- CTGGAGCAGCGGGGTGGGGCACACACCACATCC-3'	This study
Pet28a-GBP1m3	His-GBP1 KLKK207-210ALAA	5'- TGGCTCTGGCTGCTGGTACCAGTCAAAAAGATGAAACT-3'<br>5'- CAGCAGCCAGAGCCAGGGAGTATGTCAGGTACTCA-3'	This study
Pet28a-GBP1m4	His-GBP1 K	5'- TCACGCCGCCGCCCTTGCCCAGCTCGAGAAAC -3'<br>5'- AGGGCGGCGGCGTGAACGGGCCGATCAAAGAC -3'	This study
Pet28a-GBP1m5	His-GBP1 R584-586A	5' AATGGCTGCTGCTAAGGCATGTACCATAAGCTAAATG-3'<br>5'- TTAGCAGCAGCCATTTTCGTCTGGAGATCCTG-3'	This study

Supplementary Table 2 – siRNAs used for knock-down.

Oligonucleotides	Source and catalogue number
Stealth RNAi™ siRNA negative control, Med GC	Thermo Fisher Scientific (12935300)
siCASP4	Thermo Fisher Scientific (HSS141457)
siGSDMD	Thermo Fisher Scientific (HSS149278)
siGBP1	Thermo Fisher Scientific (HSS104021)
siGBP2	Thermo Fisher Scientific (HSS104025)
siGBP3	Thermo Fisher Scientific (HSS104027)
siGBP4	Thermo Fisher Scientific (HSS133000)
siGBP5	Thermo Fisher Scientific (HSS133003)
siGBP6	Thermo Fisher Scientific (HSS136383)
siGBP7	Thermo Fisher Scientific (HSS139886)

Supplementary Table 3 – Primers used for qPCR.

Target gene	Primer pairs (5' → 3')
GBP1	TCAATGAGGAAATCCCAGCCC<br>AGGCTGTTCCCTTGTCTGTTC
GBP2	ATCTCTGATCTGGGGAACAACAC<br>GATAGAGGCCCACAATCGCC
GBP3	AGCACAGACAAGAGAACAATGCC<br>TCTGGATTCGCCACCAGTTC
GBP4	CAGTGCCCACACCAGGTTATC<br>TTCCTGTGCGGTATAGCCCT
GBP5	CGGCGATTCAAAGGCAGAAC<br>AGCCTGTTCCTGCATCTGTTG
GBP6	ACTGCACCATCCCATTTGTGG<br>TGCCAACCTAGAAGAGCCTGC
GBP7	ACTCTGGACAGAGGAACGCC<br>TAGAGGCCCACAATTGCCAC
HPRT	GAACCTCTCGGCTTTCCCG<br>TCACTAATCACGACGCCAGGG

Supplementary References

1. Paz, I. *et al.* Galectin-3, a marker for vacuole lysis by invasive pathogens. *Cell Microbiol* **12,** 530–544 (2010).
2. Ray, K. *et al.* Tracking the dynamic interplay between bacterial and host factors during pathogen-induced vacuole rupture in real time. *Cell Microbiol* **12,** 545–556 (2010).
