# Supplementary material for: Human GBP1 binds LPS to initiate assembly of a caspase-4 activating platform on cytosolic bacteria
Source: Nat Commun. 2020 Jun 24;11:3276. doi: 10.1038/s41467-020-16889-z (PMC7314798; doi:10.1038/s41467-020-16889-z)
Supplement: Supplementary file 3 — Description of Additional Supplementary Information [file 41467_2020_16889_MOESM3_ESM.pdf]

Description of Additional Supplementary Files

Supplementary Movie 1. Naïve HeLa infected with *Salmonella* do not undergo cell death by pyroptosis. Time-lapse fluorescence confocal microscopy of naïve HeLa infected with *Salmonella*-dsRed. Merge of DIC and bacteria (red) is shown. After 3 h of infection it is possible to observe replicating cytosolic bacteria (hyper-replication), which display decreased dsRed signal.

Supplementary Movie 2. IFNγ-primed HeLa infected with *Salmonella* undergo pyroptosis. Time-lapse fluorescence confocal microscopy of IFNγ-primed HeLa infected with *Salmonella*-dsRed. Merge of DIC and bacteria (red) is shown. Cell pyroptosis is seen by plasma membrane ballooning and nuclear condensation. After 2 h of infection it is possible to observe replicating cytosolic bacteria (hyper-replication), which display decreased dsRed signal.

Supplementary Movie 3. GBP1 and GBP2 are recruited simultaneously to intracellular *Salmonella*. Time-lapse fluorescence confocal microscopy of IFNγ-primed HeLa co-expressing mCherry-GBP1 (red) and eGFP-GBP2 (green) and infected with *Salmonella*.

Supplementary Movie 4. GBP3 is recruited to intracellular *Salmonella* after GBP1. Time-lapse fluorescence confocal microscopy of IFNγ-primed HeLa co-expressing mCherry-GBP1 (red) and eGFP-GBP3 (green) and infected with *Salmonella*.

Supplementary Movie 5. GBP4 is recruited to intracellular *Salmonella* after GBP1. Time-lapse fluorescence confocal microscopy of IFNγ-primed HeLa co-expressing mCherry-GBP1 (red) and eGFP-GBP4 (green) and infected with *Salmonella*.

Supplementary Movie 6. GBP1 is recruited to cytosolic *Salmonella* seconds after SCV rupture. Time-lapse fluorescence confocal microscopy of IFNγ-primed HeLa co-expressing galectin-3-eGFP (green) and mCherry-GBP1 (red) and infected with *Salmonella*.

Supplementary Movie 7. GBP1 recruitment to cytosolic *Salmonella* is followed by pyroptosis. Time-lapse fluorescence confocal microscopy of IFNγ-primed HeLa expressing eGFP-GBP1 (green) and infected with *Salmonella*-dsRed (red). Middle panel: DIC shows a

cell undergoing pyroptosis as seen by plasma membrane ballooning and nuclear condensation. Right panel: composite image.

Supplementary Movie 8. GBPs do not restrict *Salmonella* growth in the cytosol of epithelial cells. Time-lapse fluorescence confocal microscopy of IFNγ-primed *GSDMD*$^{-/-}$ HeLa expressing eGFP-GBP1 (green) and infected with *Salmonella*-dsRed (red).

Supplementary Movie 9. GBPs do not restrict *Salmonella* growth in the cytosol of epithelial cells. Time-lapse fluorescence confocal microscopy of IFNγ-primed *GSDMD*$^{-/-}$ HeLa expressing eGFP-GBP1 (green) and infected with *Salmonella*-dsRed (red).

Supplementary Movie 10. GBPs do not restrict *Salmonella* growth in the cytosol of epithelial cells. Time-lapse fluorescence confocal microscopy of IFNγ-primed *GSDMD*$^{-/-}$ HeLa expressing eGFP-GBP1 (green) and infected with *Salmonella*-dsRed (red).

Supplementary Movie 11. GBPs do not restrict *Salmonella* growth in the cytosol of epithelial cells. Time-lapse fluorescence confocal microscopy of IFNγ-primed *GSDMD*$^{-/-}$ HeLa expressing eGFP-GBP1 (green) and infected with *Salmonella*-dsRed (red).

Supplementary Movie 12. Caspase-4 recruitment to intracellular *Salmonella* is followed by pyroptosis. Time-lapse fluorescence confocal microscopy of IFNγ-primed HeLa expressing caspase-4-eGFP (green) and infected with *Salmonella*-dsRed (red). Right panel: DIC shows a cell undergoing pyroptosis as seen by plasma membrane ballooning and nuclear condensation.

Supplementary Movie 13. When caspase-4 is not recruited to *Salmonella*, cells do not undergo pyroptosis. Time-lapse fluorescence confocal microscopy of IFNγ-primed HeLa expressing caspase-4-eGFP (green) and infected with *Salmonella*-dsRed (red). Right panel: DIC.

Supplementary Movie 14. Caspase-4 is recruited to cytosolic *Salmonella* after SCV rupture. Time-lapse fluorescence confocal microscopy of IFNγ-primed HeLa co-expressing galectin-3-mOrange (red) and caspase-4-eGFP (green) and infected with *Salmonella*.

Supplementary Movie 15. GBP1 recruitment to cytosolic *Salmonella* precedes caspase-4 recruitment. Time-lapse fluorescence confocal microscopy of IFNγ-primed HeLa

co-expressing mCherry-GBP1 (red) and caspase-4-eGFP (green) and infected with *Salmonella*. Right panel: DIC shows a cell undergoing pyroptosis as seen by plasma membrane ballooning and nuclear condensation.
