# Supplementary material for: Human GBP1 binds LPS to initiate assembly of a caspase-4 activating platform on cytosolic bacteria
Source: Nat Commun. 2020 Jun 24;11:3276. doi: 10.1038/s41467-020-16889-z (PMC7314798; doi:10.1038/s41467-020-16889-z)
Supplement: Supplementary file 19 — Reporting Summary [file 41467_2020_16889_MOESM19_ESM.pdf]

nature research

Corresponding author(s): Petr Broz

Last updated by author(s): Apr 14, 2020

Reporting Summary

Nature Research wishes to improve the reproducibility of the work that we publish. This form provides structure for consistency and transparency in reporting. For further information on Nature Research policies, see Authors & Referees and the Editorial Policy Checklist .

Statistics

For all statistical analyses, confirm that the following items are present in the figure legend, table legend, main text, or Methods section.

n/a	Confirmed	
☐	☒	The exact sample size (n) for each experimental group/condition, given as a discrete number and unit of measurement
☐	☒	A statement on whether measurements were taken from distinct samples or whether the same sample was measured repeatedly
☐	☒	The statistical test(s) used AND whether they are one- or two-sided<br>*Only common tests should be described solely by name; describe more complex techniques in the Methods section.*
☒	☐	A description of all covariates tested
☒	☐	A description of any assumptions or corrections, such as tests of normality and adjustment for multiple comparisons
☐	☒	A full description of the statistical parameters including central tendency (e.g. means) or other basic estimates (e.g. regression coefficient) AND variation (e.g. standard deviation) or associated estimates of uncertainty (e.g. confidence intervals)
☐	☒	For null hypothesis testing, the test statistic (e.g. F, t, r) with confidence intervals, effect sizes, degrees of freedom and P value noted<br>*Give P values as exact values whenever suitable.*
☒	☐	For Bayesian analysis, information on the choice of priors and Markov chain Monte Carlo settings
☒	☐	For hierarchical and complex designs, identification of the appropriate level for tests and full reporting of outcomes
☒	☐	Estimates of effect sizes (e.g. Cohen's d, Pearson's r), indicating how they were calculated

Our web collection on statistics for biologists contains articles on many of the points above.

Software and code

Policy information about availability of computer code

Data collection

The following commercial software was used to collect data:
GE Healthcare AKTA, Fusion Solo S, Gen5, Zen Blue 2.3 Imaging Software, LightCycler 480 Software, Biacore T200 software, MO.Affinity Analysis software (version number not available)

Data analysis

The following commercial software was used to analyze data:
Microsoft Excel for Mac v16, Prism Graphpad 8.0, Gen5, GE Healthcare Unicorn V7.3, FiJi, Zen Blue 2.3 Imaging Software, BiacoreT200 Evaluation software 3.0. MO.Affinity Analysis software

For manuscripts utilizing custom algorithms or software that are central to the research but not yet described in published literature, software must be made available to editors/reviewers. We strongly encourage code deposition in a community repository (e.g. GitHub). See the Nature Research guidelines for submitting code & software for further information.

Data

Policy information about availability of data

All manuscripts must include a data availability statement. This statement should provide the following information, where applicable:

- Accession codes, unique identifiers, or web links for publicly available datasets
- A list of figures that have associated raw data
- A description of any restrictions on data availability

The source data corresponding to Figs. 1h-i; 2a, 2d, 2e; 6a, 6f and Supplementary Figs. 1g; 2c, 2f-h; 3q; 5b; 10d, 10f; 11e; 14b are provided as Source Data files.
The source data for all other figures is available upon request to the corresponding author.

Field-specific reporting

Please select the one below that is the best fit for your research. If you are not sure, read the appropriate sections before making your selection.

- [x] Life sciences
- [ ] Behavioural & social sciences
- [ ] Ecological, evolutionary & environmental sciences

For a reference copy of the document with all sections, see nature.com/documents/nr-reporting-summary-flat.pdf

Life sciences study design

All studies must disclose on these points even when the disclosure is negative.

Sample size	No statistical method was used to determine sample sizes and no sample size calculation was performed.<br>Sample sizes were chosen according to our previous experience in similar experimental setups to generate statistically significant, publication quality values.
Data exclusions	No data were excluded from the analysis.
Replication	Experiments were repeated, when possible, by different experimenters to ensure the reproducibility of the experiment. Each experiment was repeated at least 3 time, unless stated otherwise in the figure legend. The number of replicate performed for each figure is clearly stated in the figure legends.
Randomization	There was no randomization for these experiments. This study is not a randomised control trial and randomisation is not conventionally used in in vitro/in cellulo studies such as this one. All groups of experiments were performed using the same experimental conditions and protocols.
Blinding	Assays used straightforward quantification methods that are not susceptible to bias, so samples were not blinded.

Reporting for specific materials, systems and methods

We require information from authors about some types of materials, experimental systems and methods used in many studies. Here, indicate whether each material, system or method listed is relevant to your study. If you are not sure if a list item applies to your research, read the appropriate section before selecting a response.

Materials & experimental systems

n/a	Involved in the study	
	x	Antibodies
	x	Eukaryotic cell lines
x		Palaeontology
x		Animals and other organisms
	x	Human research participants
x		Clinical data

Methods

n/a	Involved in the study	
x		ChIP-seq
x		Flow cytometry
x		MRI-based neuroimaging

Antibodies

Antibodies used	Mouse monoclonal Anti caspase-4 clone 4B9 (Enzo life Sciences, cat #ADI-AAH-114-5, lot 10091704)<br>Rabbit polyclonal anti-GBP1 (Abcam, cat# ab121039)<br>Mouse monoclonal anti-GFP clone JL-8 (Clonetech, cat # 632381, lot A8034133)<br>Mouse monoclonal anti-GAPDH clone 6C5 (ThermoFischer Scientitic, cat# AM4300)<br>Mouse monoclonal anti-V5 (ThermoFischer Scientific, cat# R960-25)<br>Rabbit polyclonal anti-Lamp1 (Abcam, cat# ab24170)<br>Mouse monoclonal anti HA, clone 16B12 (Enzo Life Sciences, cat ENZ-ABS-118-0200)<br>Rabbit monoclonal anti-GSDMD (Abcam, cat# 210070, lot GR3199122-8)<br>Mouse monoclonal anti-Tubulin clone DM1A (Abcam, cat# ab40742)
Validation	Anti-Caspase-4 antibody (Enzo life Sciences, cat #ADI-AAH-114-5, lot 10091704), anti-GSDMD (Abcam, cat# 210070, lot GR3199122-8) and anti-GBP1 (Abcam, cat# ab121039) were validated using knockout cell lines, validated by the suppliers and are extensively used in the scientific community.<br>Anti-GFP clone JL-8 (632375, Takara), anti-V5 (ThermoFischer Scientific, cat# R960-25) and anti-HA (ENZ-ABS-118-0200,Enzo Life Sciences) have been validated by overexpressing protein tagged with those epitopes and have been validated by their respective manufacturers. Anti-GAPDH (ThermoFischer Scientitic, cat# AM4300), anti-Lamp1 (Abcam, cat # ab24170) and anti-tubulin (Abcam, cat# b40742) have been validated by their manufacturer and are extensively used in the scientific community.

Eukaryotic cell lines

Policy information about cell lines

Cell line source(s)	HeLa clone CCL-2 (obtained from ATCC)<br>HeLa Kyoto (a gift from Prof Jean Pieters, Biozentrum Basel)<br>HBEC3-KT clone CRL-4051 (obtained from ATCC)<br>HT-29 (from ATCC, a kind gift from Dr. Shaynoor Dramsi, Institut Pasteur, Paris)<br>Caco2-TC7 (from ATCC, a kind gift from Dr. Shaynoor Dramsi, Institut Pasteur, Paris)<br>U937 clone CRL-1593.2 (obtained from ATCC)<br>THP-1 clone TIB-202 (obtained from ATCC)<br>HIEC-6 (obtained from ATCC)<br>HaCaT (obtained from CLS Cell Lines Service GmbH)
Authentication	The identity of cell lines was frequently checked by their morphological features and did not show any signs of cross-contamination. However they have not been authenticated by short tandem repeat (STR) profiling.
Mycoplasma contamination	Cell lines are regularly tested in the lab for mycoplasma contamination and are mycoplasma free.
Commonly misidentified lines<br>(See ICLAC register)	No commonly misidentified cell lines were used in this study

Human research participants

Policy information about studies involving human research participants

Population characteristics	The experiments performed using Human primary monocyte-derived macrophages did not involve volunteers. Rather, blood from healthy donors was supplied by the Swiss Red Cross. Except for the blood type, no information on the participants was provided by the Swiss Red Cross.
Recruitment	Participants were not recruited for collecting blood. Instead, blood from healthy donors was supplied by the Swiss Red Cross. Except for the blood type, no information on the participants was provided by the Swiss Red Cross.
Ethics oversight	Blood collection from healthy donors was approved by the Swiss Red Cross.

Note that full information on the approval of the study protocol must also be provided in the manuscript.
