# Supplementary material for: Human GBP1 binds LPS to initiate assembly of a caspase-4 activating platform on cytosolic bacteria
Source: Nat Commun. 2020 Jun 24;11:3276. doi: 10.1038/s41467-020-16889-z (PMC7314798; doi:10.1038/s41467-020-16889-z)
Supplement: Supplementary file 20 — Source Data [file 41467_2020_16889_MOESM20_ESM.pdf]

Fig. 1h

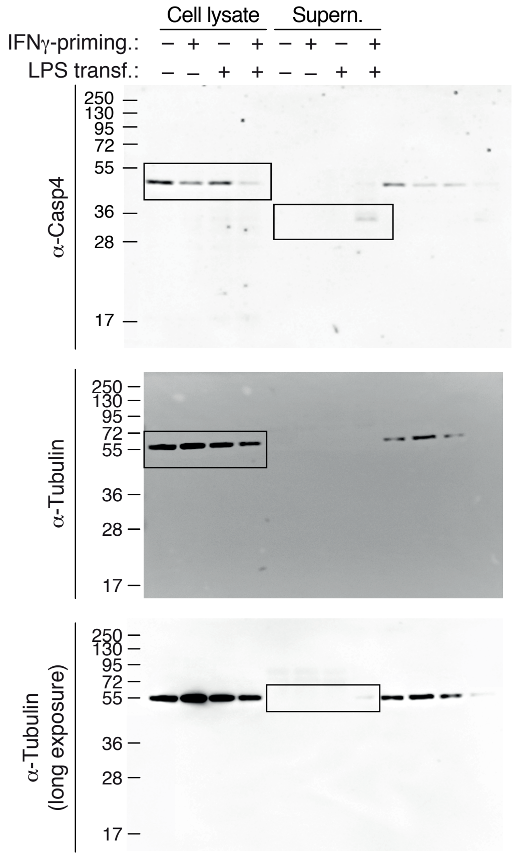

Fig. 1i

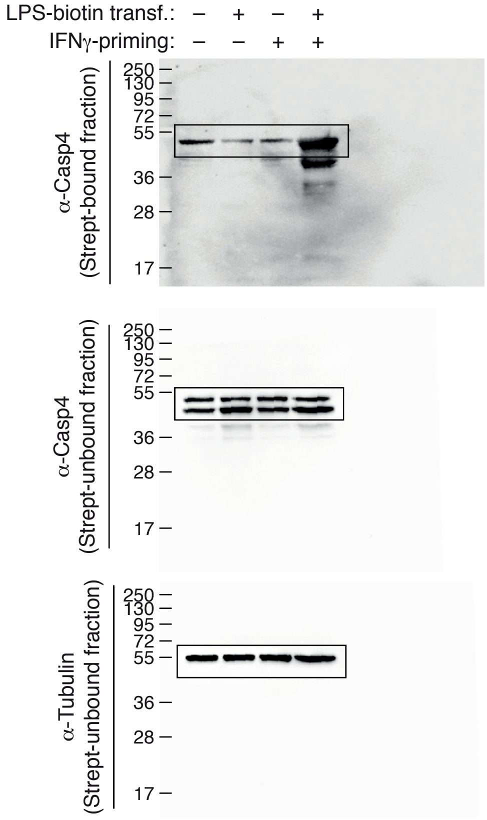

Suppl. Fig. 1g

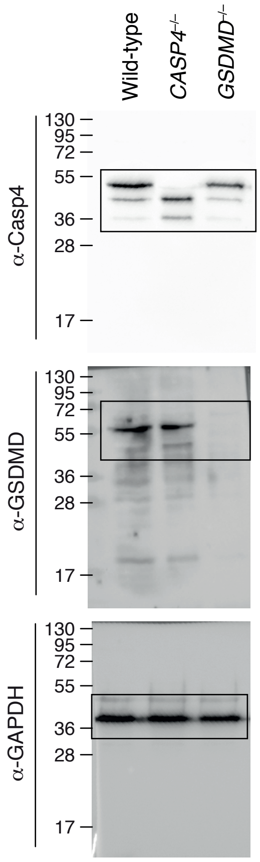

Fig. 2a

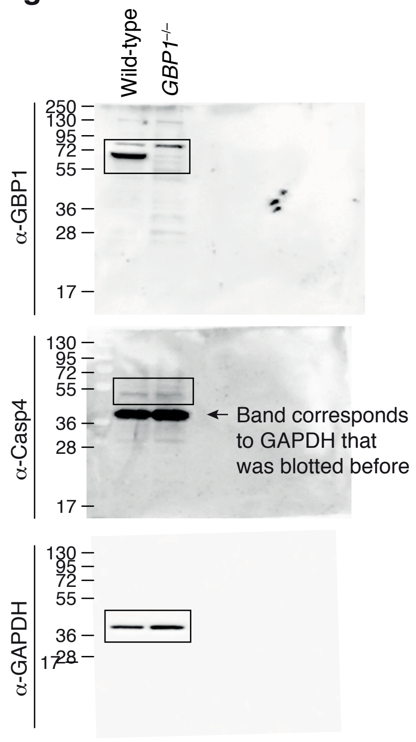

Fig. 2d

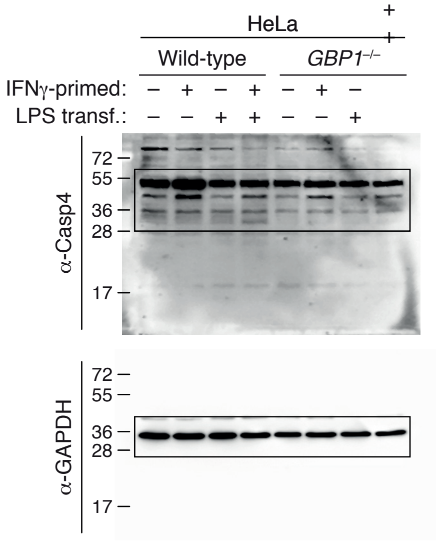

Fig. 2e

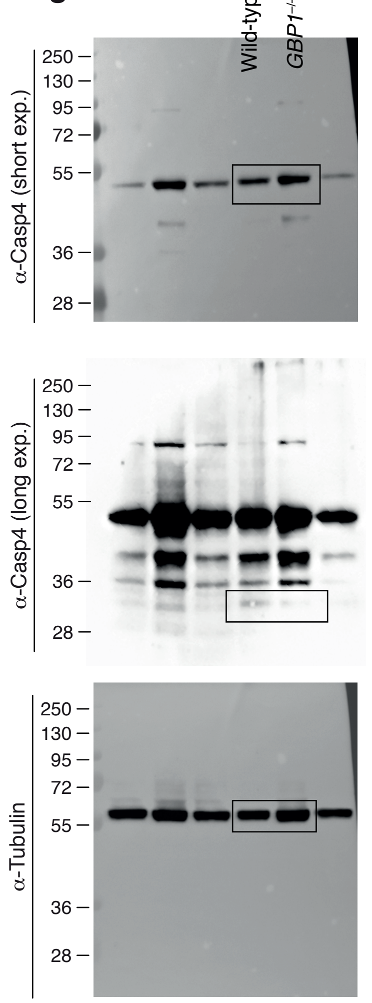

Suppl. Fig. 2c

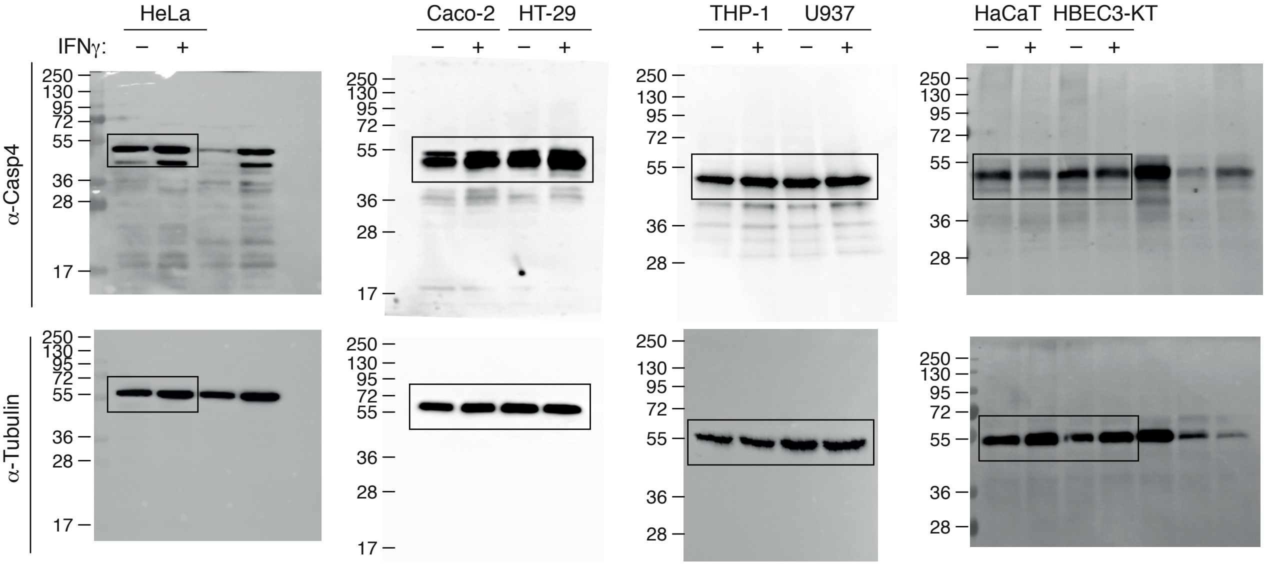

Suppl. Fig. 2f

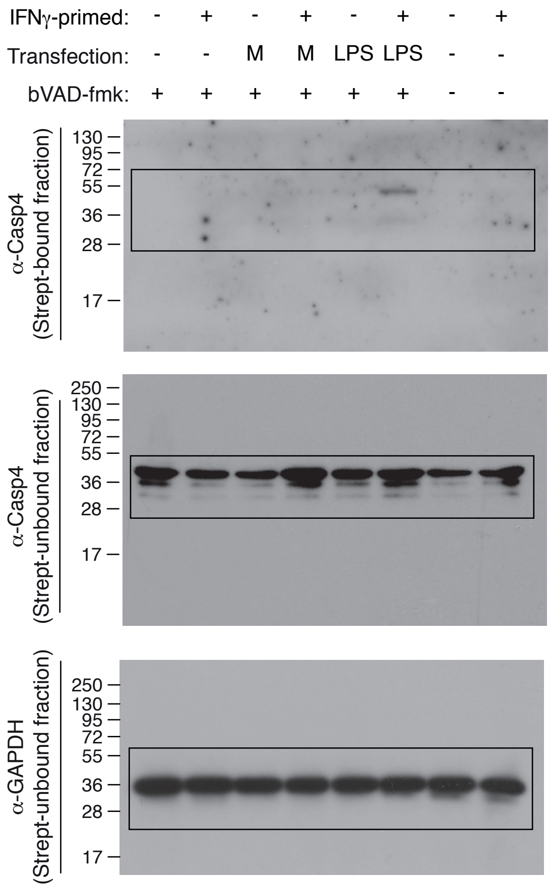

Suppl. Fig. 2g

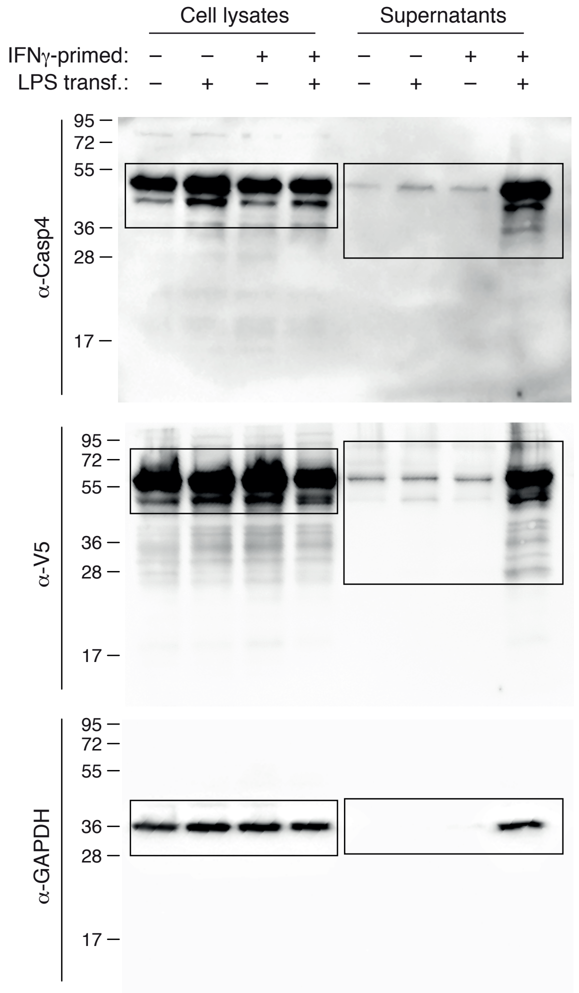

Suppl. Fig. 2h

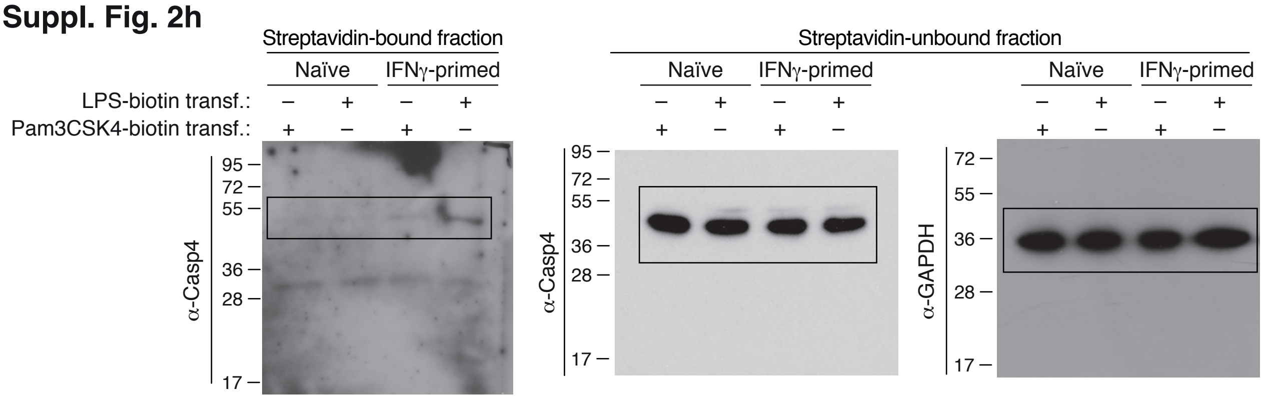

Suppl. Fig. 3q

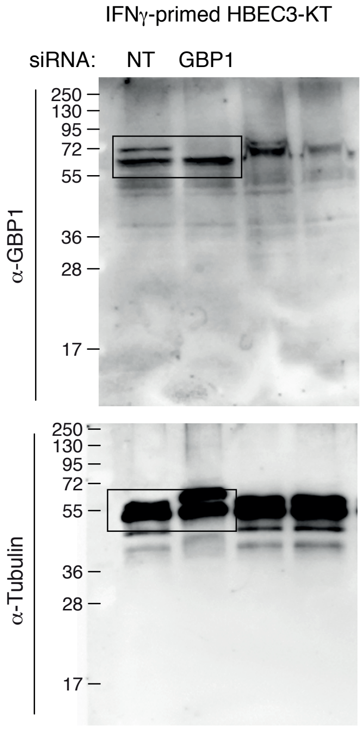

Suppl. Fig. 3q

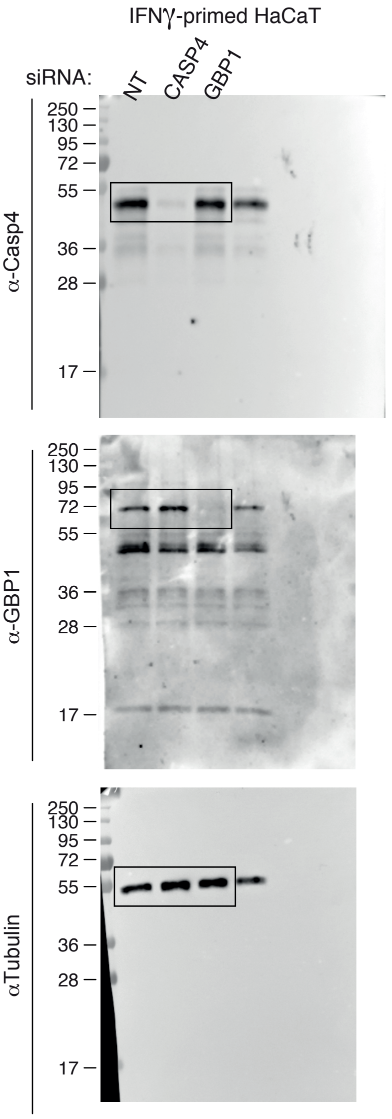

Suppl. Fig. 5b

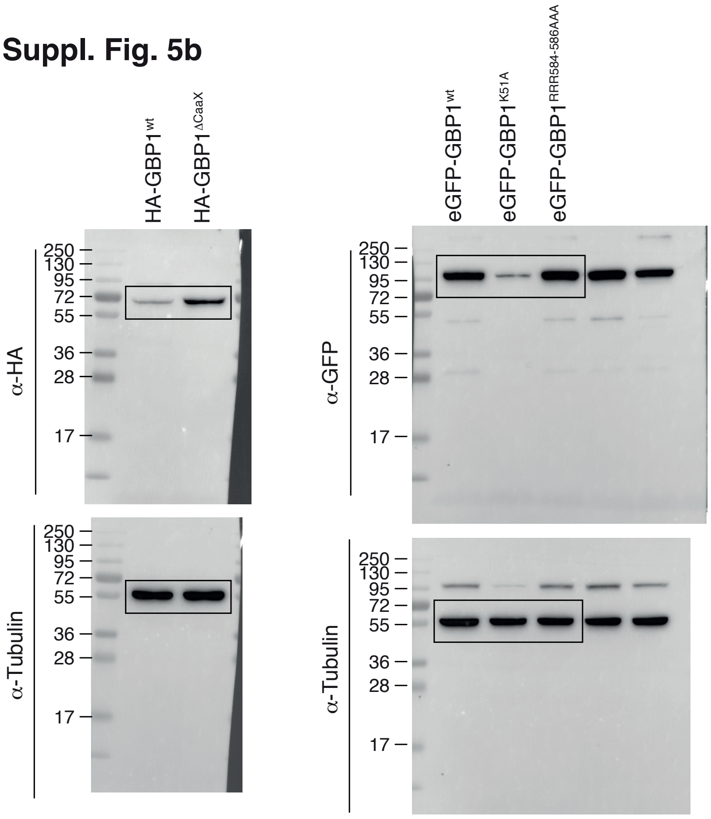

Suppl. Fig. 10d

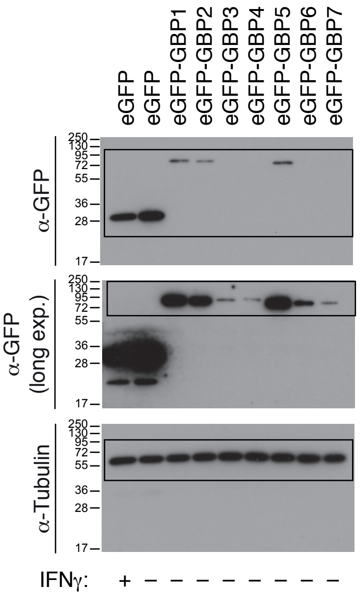

Suppl. Fig. 10f

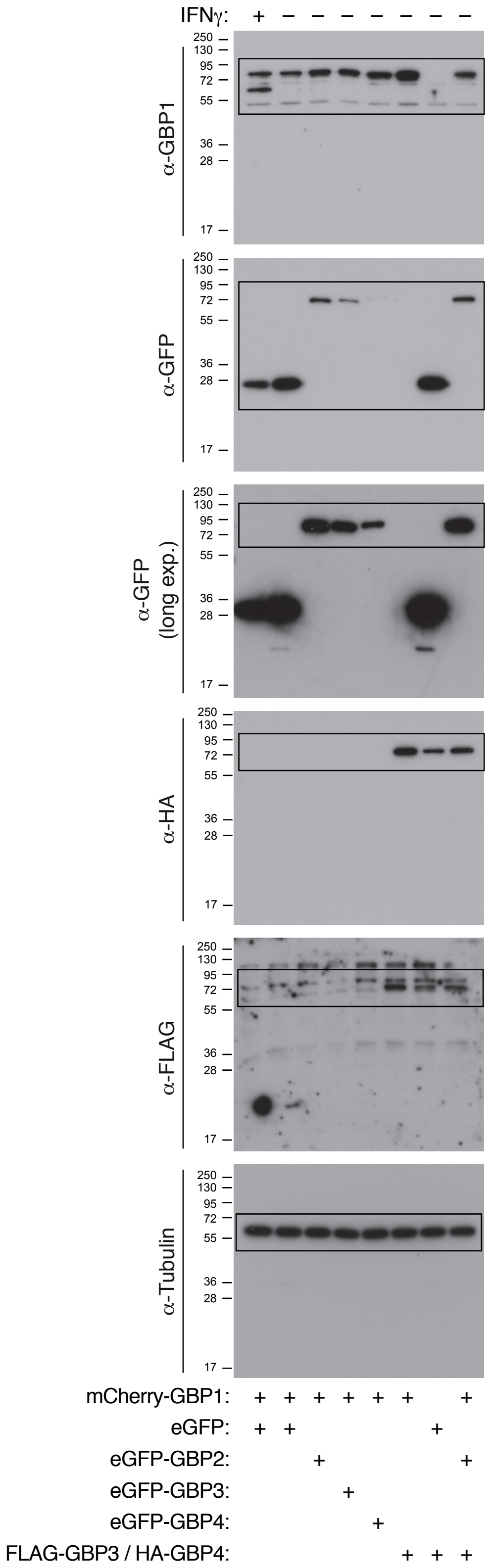

Fig. 6a

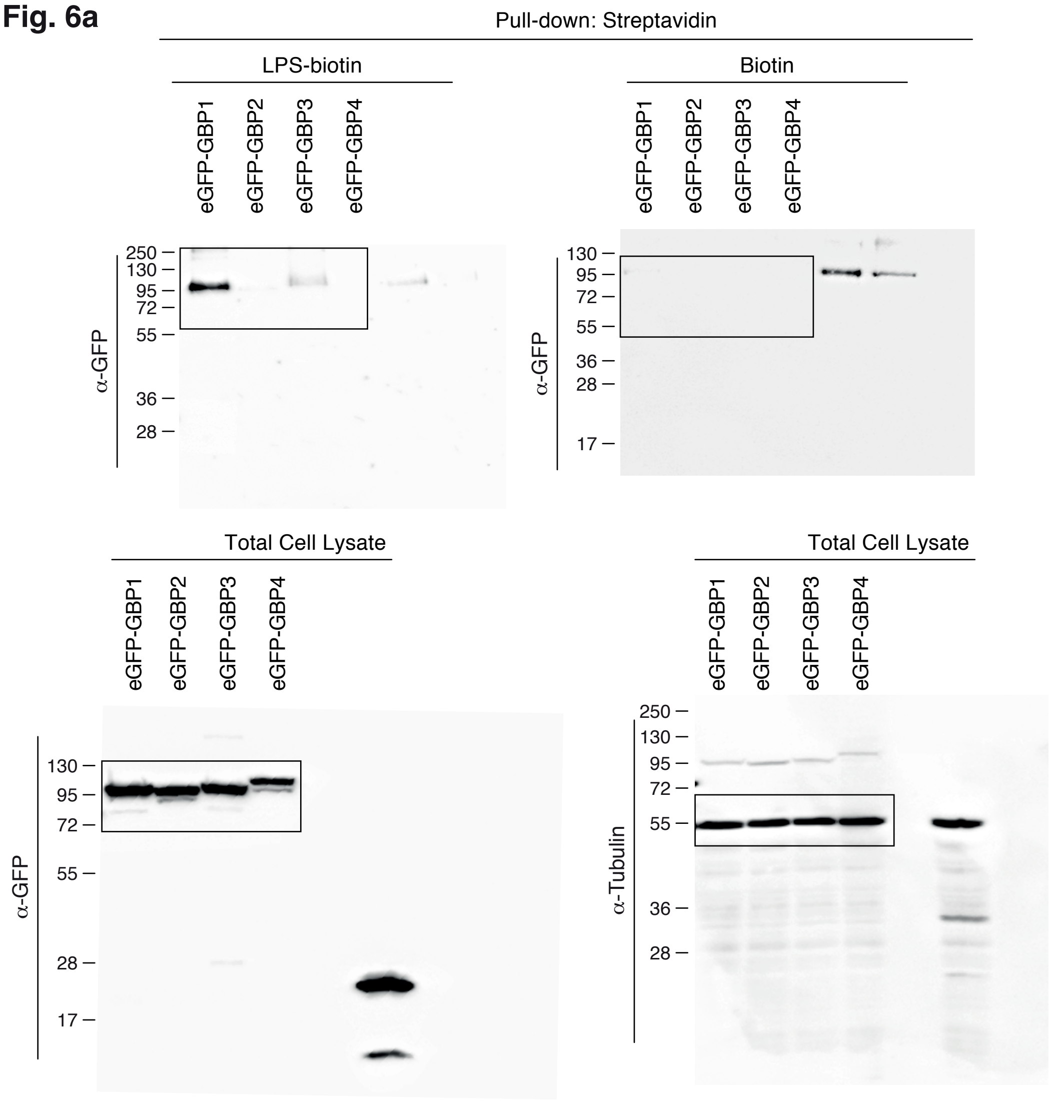
Pull-down: Streptavidin
LPS-biotin
Biotin
eGFP-GBP1
eGFP-GBP2
eGFP-GBP3
eGFP-GBP4
α-GFP
250
130
95
72
55
36
28
17
Total Cell Lysate
α-Tubulin

Fig. 6f

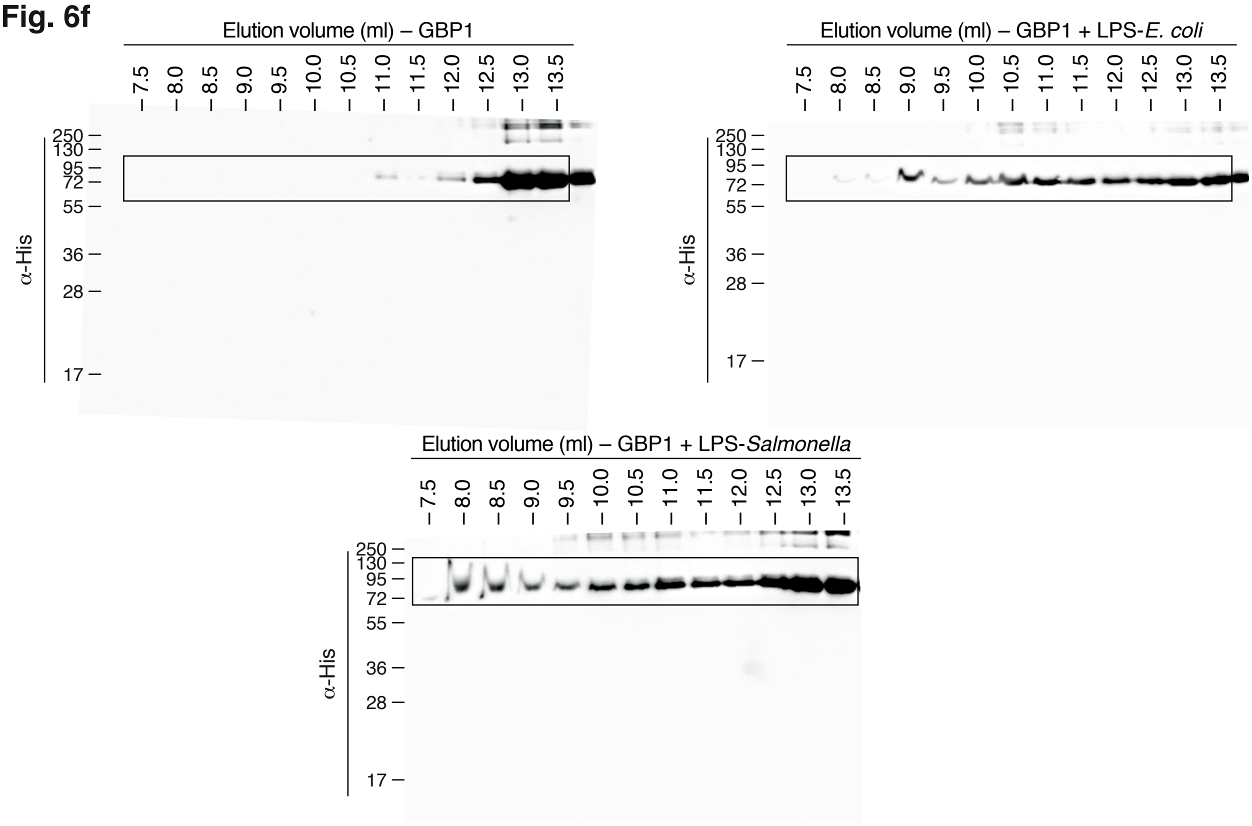
Elution volume (ml) – GBP1
Elution volume (ml) – GBP1 + LPS-*E. coli*
Elution volume (ml) – GBP1 + LPS-*Salmonella*
7.5
8.0
8.5
9.0
9.5
10.0
10.5
11.0
11.5
12.0
12.5
13.0
13.5
α-His
250
130
95
72
55
36
28
17

Suppl. Fig. 11e

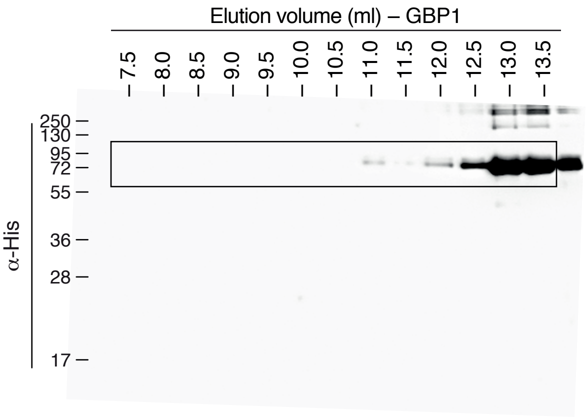

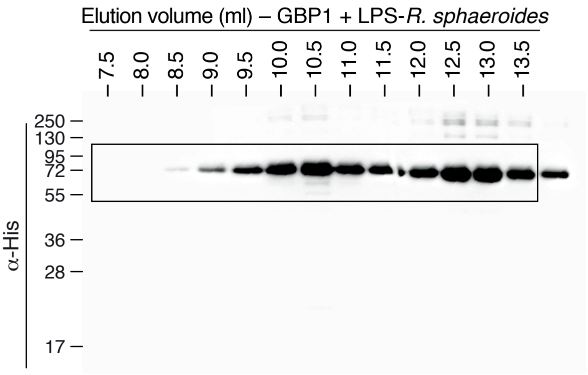

Suppl. Fig. 14b

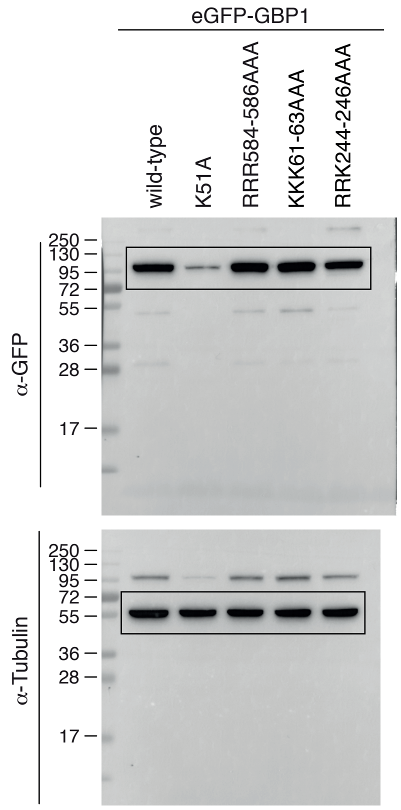

FIGURE	CONDITIONS COMPARED	P-value
Fig 1a	6 hrs: naïve vs IFNg	1.23E-06
Fig 1b	1 hr: naïve vs IFNg	2.55E-10
	6 hr: naïve vs IFNg	1.28E-14
Fig 1c	1 hr: wild-type; Naïve vs IFNg	1.55E-07
	6 hr: wild-type; Naïve vs IFNg	2.91E-10
Fig 1e	1 hr: wild-type; Naïve vs IFNg	1.74E-07
	6 hr: wild-type; Naïve vs IFNg	3.28E-04
Fig 1f	HeLa CCL2: naïve vs IFNg	4.10E-16
	HeLa Kyoto: naïve vs IFNg	3.07E-10
	HBEC: naïve vs IFNg	5.07E-06
	HaCaT: naïve vs IFNg	2.20E-08
	HIEC-6: naïve vs IFNg	1.65E-06
	Caco2: naïve vs IFNg	1.29E-06
	HT29: naïve vs IFNg	4.81E-09
	THP1: naïve vs IFNg	1.60E-05
	U937: naïve vs IFNg	3.87E-08
Fig 1g	Wild-type LPS: naïve vs IFNg	7.54E-06
Fig 2b	Wild-type: naïve vs IFNg	1.87E-11
	IFNg: wild-type vs GBP1-/-	2.78E-08
Fig 2c	Wild-type: naïve vs IFNg	1.12E-07
	IFNg: wild-type vs GBP1-/-	2.72E-11
Fig 2f	LPS: wild-type vs GBP1-/-	3.52E-04
Fig 2g	HBEC: NT vs CASP4	1.25E-10
	HBEC: NT vs GSDMD	3.05E-08
	HBEC: NT vs GBP1	3.32E-07
	HaCaT: NT vs CASP4	4.61E-09
	HaCaT: NT vs GSDMD	1.57E-07
	HaCaT: NT vs GBP1	8.21E-06
Fig 4j	wild-type vs GBP1-/-	8.67E-20
Fig 5c	GBP1 vs GBP2	0.13
	GBP1 vs GBP3	0.04
	GBP1 vs GBP4	9.65E-07
Fig 5d	GBP1/GFP vs GBP1/GBP4	0.002
	GBP1/GFP vs GBP1/3/4	< 0.001
	GBP1/GFP vs GBP1/2/3/4	< 0.001
Fig 7b		2.10E-06
Fig 7f	wild-type vs KKK61-63AAA	5.40E-08
	wild-type vs KK87-88AA	0.050

FIGURE	CONDITIONS COMPARED	P-value
Sup Fig 1e	1 hr: naïve vs IFNg	0.002
	6 hr: naïve vs IFNg	7.90E-06
	10 hr: naïve vs IFNg	9.25E-04
Sup Fig 1f	Salmonella 6h p.i.	0.02
Sup Fig 1l	6 hrs: wild-type; naïve vs IFNg	0.001
	6 hrs: CASP4-/-; naïve vs IFNg	0.01
	6 hrs: GSDMD-/-; naïve vs IFNg	0.04
Sup Fig 1q	naïve vs IFNg	4.03E-05
Sup Fig 1s	naïve vs IFNg	0.002
Sup Fig 2b	Donor 1	2.09E-05
	Donor 2	5.67E-06
Sup Fig 2d	HeLa LPS transf.	0.005
	HBEC3 LPS transf.	0.045
	HIEC-6: LPS transf.	0.01
Sup Fig 2e	HeLa: LPS electroporation	3.96E-09
	HBEC: LPS electroporation	4.65E-04
Sup Fig 3e	NT vs GBP1	0.004
	NT vs GBP2	0.02
	NT vs GBP3	<0.001
Sup Fig 3f	NT vs GBP1	<0.001
	NT vs GBP2	<0.001
	NT vs GBP3	<0.001
Sup Fig 3g	NT vs GBP1	<0.001
	NT vs GBP2	0.04
	NT vs GBP3	0.02
Sup Fig 3o	6 hr: Wild-type ; naïve vs IFNg	1.23E-06
	6 hr: GBP1-/- ; naïve vs IFNg	5.89E-06
Sup Fig 3p	naïve vs IFNg	0.001
Sup Fig 5c	wt vs R48A	4.91188E-07
	wt vs RRR584-586AAA	1.50726E-07
Sup Fig 10e	GBP1/GFP vs GBP1/GBP4	0.04
	GBP1/GFP vs GBP1/3/4	<0.001
	GBP1/GFP vs GBP1/2/3/4	<0.001
