# Supplementary material for: Human GBP1 binds LPS to initiate assembly of a caspase-4 activating platform on cytosolic bacteria
Source: Nat Commun. 2020 Jun 24;11:3276. doi: 10.1038/s41467-020-16889-z (PMC7314798; doi:10.1038/s41467-020-16889-z)
Supplement: Supplementary file 2 — Peer Review File [file 41467_2020_16889_MOESM2_ESM.pdf]

Editorial Note: This manuscript has only been reviewed at another journal that is not operating a transparent peer review scheme.
